# Dynamic transcriptomic landscape of myogenesis in Muscovy ducks (*Cairina moschata*): integrative analysis of hub genes post-hatching

**DOI:** 10.5713/ab.25.0159

**Published:** 2025-08-12

**Authors:** Xiaofeng Li, Kewei Fan, Bing Yang

**Affiliations:** 1 College of Animal Science and Technology, Ningxia University, Yinchuan, China; 2 Longyan University & Fujian Provincial Key Laboratory for Prevention and Control of Animal Infectious Diseases and Biotechnology, Longyan University, Longyan, China; 3 Anhui Province Key Laboratory of Embryo Development and Reproductive Regulation, Fuyang Normal University, Fuyang, China

**Keywords:** Hub Genes, Muscovy Duck, Myogenesis, Post-hatching

## Abstract

**Objective:**

Post-hatching myogenesis is a critical determinant of meat yield and quality, with potential regulatory roles of specific genes remaining underexplored in Muscovy ducks (*Cairina moschata*). This study aimed to identify hub genes governing post-hatching myogenesis through transcriptomic profiling.

**Methods:**

Three white-feathered male Muscovy ducks at 1-day-old (1D) and 80-day-old (80D) were selected, respectively. Following humane euthanasia, the left leg muscles were collected for subsequent RNA extraction, library construction, genome alignment, and transcriptome sequencing. Differential gene expression analysis was performed using DESeq2 (ver. 1.16.1), with significant differentially expressed genes (DEGs) selected under the thresholds of |log2FC|>1 and p-value<0.05. Subsequent functional characterization included enrichment analyses (Gene Ontology, KEGG, and PANTHER pathways) implemented through DAVID and KOBAS, followed by protein-protein interaction network modeling via STRING. Topological analysis with CytoHubba further pinpointed hub genes functionally linked to myogenesis.

**Results:**

Totally 1,683 DEGs were identified between 80D and 1D muscles, including 865 upregulated and 818 downregulated genes. Totally ten hub genes, such as *CD3E*, *ITK*, *COL4A6*, and *IVD*, were prioritized as key regulators of myogenesis. Functional analysis revealed their enrichment in L-leucine catabolic processes and fatty acid β-oxidation. Pathway mapping further associated these genes with glycine/serine/threonine metabolism, and branched-chain amino acid degradation.

**Conclusion:**

This study delineates a molecular framework for post-hatching myogenesis in Muscovy ducks, highlighting ten hub genes that orchestrate myogenesis through metabolic reprogramming. These findings advance genetic strategies for improving poultry meat production and quality.

## INTRODUCTION

Post-hatching skeletal myogenesis serves as a fundamental determinant of both meat yield and quality in poultry production systems, with significant implications for economic viability and consumer acceptance [1,2]. In avian species, this developmental process encompasses precisely regulated phases of myoblast proliferation, differentiation, and hypertrophic expansion - all orchestrated through complex transcriptional cascades and metabolic reprogramming. Notably, Muscovy ducks (*Cairina moschata*) exhibit exceptional muscular development characteristics, achieving 28%–32% leg muscle yield coupled with remarkably low fat deposition (10%–15%), thereby outperforming conventional poultry species (chickens, ducks, and geese) in leanness metrics. The functional importance of leg muscles in Muscovy ducks extends beyond meat production, as these muscles are biomechanically crucial for locomotion and weight-bearing, further underscoring their significance as key targets for genetic and nutritional optimization strategies [3]. Muscovy duck muscle development progresses through four key phases: 1) neonatal (0–7 days): hyperplasia establishes basic structures; 2) rapid growth (1–4 weeks): hypertrophy begins with 3–5×protein deposition; 3) maturation (4–8 weeks): hypertrophy dominates as slow-twitch fibers increase; 4) stabilization (>8 weeks): growth slows, fibers stabilize, reaching slaughter age at ~80 days. The development of key meat quality attributes, including tenderness and intramuscular fat deposition, is intrinsically associated with the coordinated regulation of myogenic processes and metabolic pathways. Notably, branched-chain amino acid catabolism coupled with fatty acid β-oxidation serves dual roles: providing energetic substrates for muscle growth while simultaneously regulating satellite cell dynamics and proteosynthetic efficiency [4,5].

Despite advancements in mammalian models, the molecular mechanisms underlying post-hatching myogenesis in Muscovy ducks remain poorly understood, limiting the development of precision breeding strategies to enhance meat traits. Addressing this gap is urgent, as global demand for high-quality poultry protein continues to escalate amid challenges of sustainability and resource efficiency. Recent transcriptomic studies in poultry have revealed both conserved regulators of myogenesis (e.g., *MYOD1*, *MSTN*) and species-specific metabolic adaptors [6]. In Muscovy ducks, rapid post-hatching muscle growth is hypothesized to reflect evolutionary adaptations to high-energy demands and environmental stressors. Emerging evidence highlights the critical roles of genes involved in amino acid degradation (e.g., *IVD*, *MCCC2*) and lipid metabolism (e.g., *HADHB*, *ACAA2*) in maintaining energy homeostasis during muscle hypertrophy [7–10]. Intriguingly, immune-related gene *CD3E* was associated with meat quality by regulating meat color, pH and muscle fat content [11,12]. Furthermore, *GNMT* and *SARDH*, implicated in folate-mediated one-carbon metabolism [13,14], could modulate epigenetic landscapes to influence myogenic transcription factor expression. Despite these insights, the functional hierarchy of hub genes coordinating metabolic reprogramming during duck myogenesis remains unresolved.

Through an integrative approach combining transcriptomic profiling (RNA-seq) and protein interactome mapping, this investigation delineates key regulatory hubs governing leg muscle development during the post-hatching phase of White Muscovy ducks. Through comparative transcriptomic profiling of male ducks at two critical developmental stages - initial post-hatching (1-day-old, ID) and mature growth phase (80-day-old, 80D) - we identify stage-specific transcriptional regulators and delineate their functional contributions to metabolic adaptation during muscle development. The findings provide dual scientific value: (1) advancing fundamental knowledge of molecular mechanisms underlying avian muscle growth, and (2) offering practical biomarkers for optimizing meat production efficiency in commercial duck farming. By establishing connections between transcriptomic dynamics and metabolic control networks, this research contributes to sustainable solutions for meeting global protein demands through precision poultry breeding approaches.

## MATERIALS AND METHODS

### Animal and muscle sample collection

Six white-feathered male Muscovy ducks at 80D and 1D were purchased from Anqing Yongqiang Agricultural Technology. respectively. All animals accessed to water and feed *ad libitum*. Prior to muscle sample collection, the ducks were euthanized humanely via intravenous injection of pentobarbital sodium at a dose of 150 mg/kg body weight. Approximately 4 grams of muscle tissue were harvested from the left leg of each duck. The samples were then rinsed with RNase-free water and promptly frozen in liquid nitrogen for subsequent analysis.

### RNA isolation and quality assessment

Muscle tissue-derived total RNA was isolated utilizing Trizol reagent (Invitrogen) according to the manufacturer’s protocol. RNA integrity assessment was performed on a Bioanalyzer 2100 system (Agilent Technologies) equipped with RNA Nano 6000 assay kit, while purity evaluation was conducted via NanoPhotometer spectrophotometry (IMPLEN). Three representative RNA samples per group meeting stringent quality criteria (OD260/280 ratio>1.8, RIN>7.0) were subsequently processed for transcriptomic analysis.

### Library preparation and sequencing

RNA sequencing libraries were prepared from 1 μg qualified RNA per sample using NEBNext UltraTM RNA Library Prep Kit for Illumina (NEB), with unique barcodes assigned for sample multiplexing. Cluster generation was achieved on cBot platform (TruSeq PE Cluster Kit v3-cBotHS; Illumina), followed by 150 bp paired-end sequencing on Illumina HiSeq 2000 system (BGI).

### Primary data processing

Sequencing raw data underwent initial quality control through CASAVA pipeline (ver. 1.8.2; Illumina) for FASTQ conversion. Adapter sequences, poly-N reads, and low-quality bases (Q<20) were filtered using custom Perl scripts. Data quality metrics including Q20/Q30 scores, error rates, and GC content were systematically evaluated prior to downstream analysis.

### Genome alignment

Clean reads were aligned to the Muscovy duck reference genome (NCBI Assembly ID: 1498951) using HISAT2 (ver. 2.0.5) with default parameters. The reference genome index was pre-built to ensure efficient and accurate mapping of sequencing reads.

### Differential expression profiling

Gene expression quantification was performed via FeatureCounts (ver. 1.5.0) with FPKM normalization. Statistical analysis of differentially expressed genes (DEGs) between developmental stages was conducted using DESeq2 (ver. 1.16.1), applying negative binomial distribution modeling with Benjamini-Hochberg correction (|log2FC|>1, p<0.05).

### Functional annotation

DEGs were subjected to comprehensive functional annotation through DAVID platform (ver. 6.8), including Gene Ontology (GO) term enrichment and KEGG pathway analysis. Complementary classification was performed using PANTHER database ( http://pantherdb.org) for protein categorization.

### Interaction network construction

Protein-protein interaction networks were generated from DEG sets using STRING (ver. 12.0) with high-confidence interaction scores (>0.7). Network visualization and topological analysis were implemented in Cytoscape environment (ver. 3.8.0).

### Hub gene identification and functional annotation

The identification of myogenesis-related hub genes was achieved through comprehensive topological analysis of the protein-protein interaction network using CytoHubba (ver. 3.8.0), a Cytoscape plugin implementing three distinct centrality algorithms: maximal clique centrality (MCC) for identifying pivotal nodes within maximal fully-connected subnetworks, maximum neighborhood component (MNC) for detecting locally dense connectivity modules, and density of maximum neighborhood component (DMNC) quantifying network compactness through edge density ratios. A consensus set of high-confidence hub genes was derived from the intersection of these algorithmically independent predictions, ensuring methodological robustness. Functional characterization of these candidate regulators involved systematic literature mining complemented by cross-referencing authoritative databases including NCBI (https://www.ncbi.nlm.nih.gov/) and GeneCards (https://www.genecards.org/), with particular emphasis on their documented roles in skeletal muscle development processes.

## RESULTS

### Transcriptomic profiling of muscovy duck muscle development

**Figure 1:**
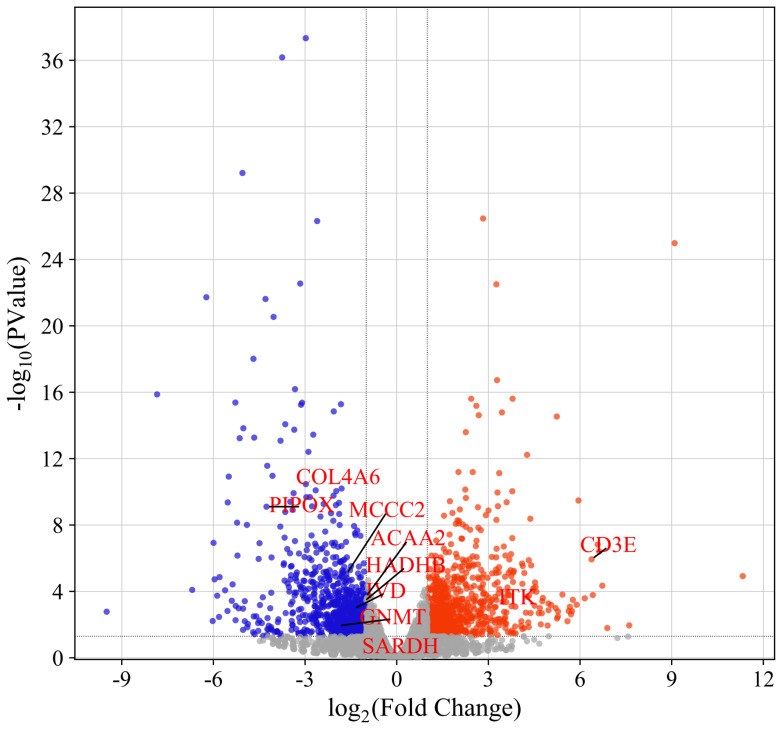
Volcano plot of differentially expressed genes (DEGs) in leg muscles of Muscovy duck at 1D and 80D post-hatching. Red dots revealed significantly upregulated genes, blue dots denote downregulated genes, and gray dots represent non-DEGs. Genes exhibiting specially annotated symbols denote potential hub candidates.

High-throughput sequencing of leg muscle tissues from 80D and 1D Muscovy ducks yielded comprehensive transcriptomic data, with 15,271 transcripts and 12,202 protein-coding genes successfully annotated. Comparative transcriptome analysis revealed 1,683 DEGs between developmental stages, comprising 865 upregulated and 818 downregulated genes in 80D samples (Supplement 1 and Figure 1). Notably, the top 30 most significantly upregulated genes (e.g., *JCHAIN*, *METTL21C*, and *ALDH1A1*) and downregulated genes (e.g., *BD2*, *CHRM2*, and *DBX2*) were systematically cataloged in Tables 1 and 2 respectively, providing crucial candidates for subsequent functional investigations into avian muscle development.

### Functional characterization of differentially expressed genes in Muscovy duck myogenesis

**Figure 2:**
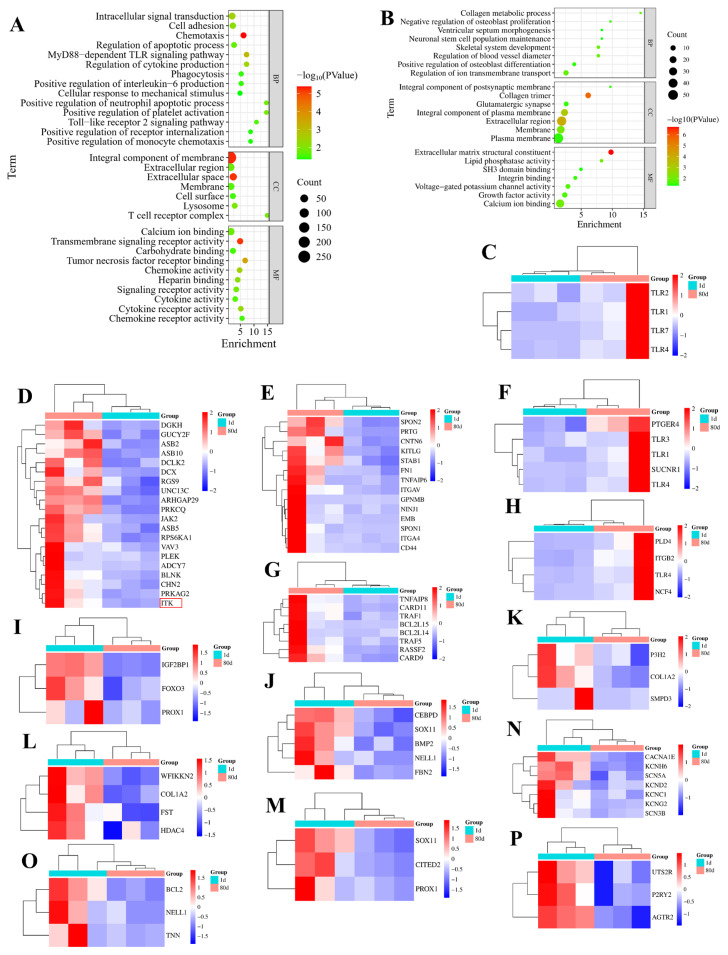
GO enrichment analysis for the DEGs. (A, B) illustrated the GO enrichment analysis for the up- and down-regulated DEGs in 80D muscle, respectively. Upregulated DEGs were predominantly involved in intracellular signal transduction, cell adhesion, chemotaxis, phagocytosis, and in regulating apoptosis and cytokine production. Conversely, downregulated genes were significantly enriched in developmental processes such as skeletogenesis, extracellular matrix remodeling, and neural stem cell maintenance, along with regulating ion homeostasis and vascular tone. They also inhibited osteoblast proliferation while stimulating osteogenic differentiation. (C–P) indicated the expression outline for the DEGs between 80D and 1D in MyD88-dependent TLR, intracellular signal transduction, cell adhesion; the positive regulation of inflammatory response; apoptotic process regulation, phagocytosis, neuronal stem cell population maintenance, the positive regulation of osteoblast differentiation; collagen metabolic process, skeletal system development, ventricular septum morphogenesis, ion transmembrane transport regulation, the negative regulation of osteoblast proliferation; and blood vessel diameter regulation, respectively. Furthermore, the genes highlighted with red borders represent potential key candidates. GO, Gene Ontology; DEG, differentially expressed gene.

Based on GO terms meeting statistical significance (p<0.05) and prior published evidence, we visualized GO enrichment for significantly up- and down-regulated genes in 80D muscle tissue. Upregulated DEGs showed predominant involvement in intracellular signal transduction, cell adhesion, chemotaxis, phagocytosis, apoptotic process regulation, and cytokine production regulation (Figure 2A and Supplement 2). In contrast, downregulated genes were significantly enriched in developmental processes including skeletogenesis, extracellular matrix remodeling, and neural stem cell maintenance, along with regulatory functions in ion homeostasis and vascular tone modulation, while demonstrating inhibitory effects on osteoblast proliferation and stimulatory roles in osteogenic differentiation (Figure 2B and Supplement 3).

Additionally, Figures 2C–2P reveals the expression outline for the DEGs between 80D and 1D in MyD88-dependent TLR, intracellular signal transduction, cell adhesion; the positive regulation of inflammatory response; apoptotic process regulation, phagocytosis, neuronal stem cell population maintenance, the positive regulation of osteoblast differentiation; collagen metabolic process, skeletal system development, ventricular septum morphogenesis, ion transmembrane transport regulation, the negative regulation of osteoblast proliferation; and blood vessel diameter regulation, respectively.

### KEGG enrichment of the differentially expressed genes

**Figure 3:**
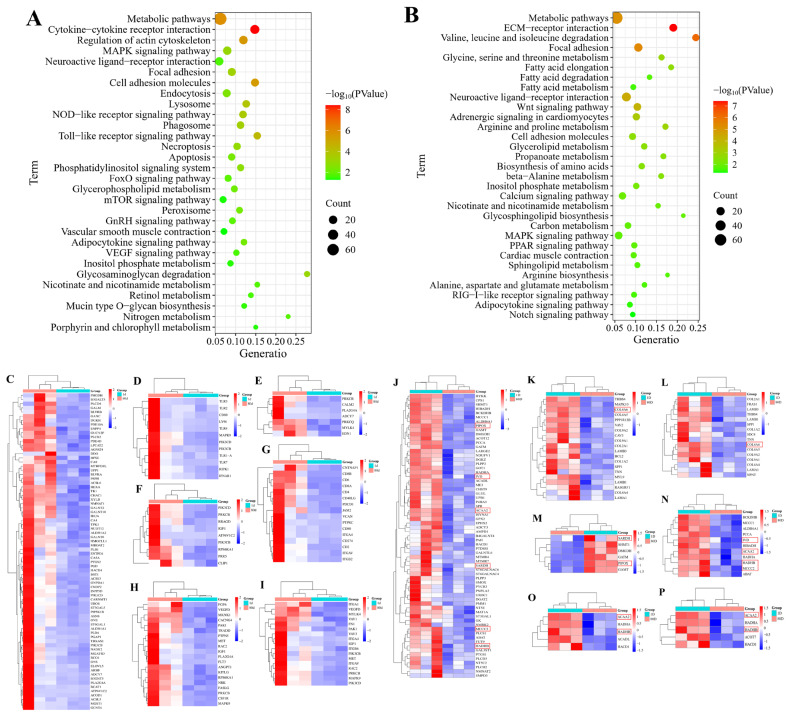
KEGG enrichment for the DEGs. (A, B) illustrated the KEGG enrichments for the up- and down-regulated DEGs in 80D muscle. Upregulated DEGs in 80D leg muscle participated in key pathways like MAPK, VEGF, mTOR, FoxO, lysosomal function, and metabolic regulation (including adipocytokine and nitrogen metabolism), alongside cell adhesion and vascular smooth muscle contraction. Conversely, downregulated genes were associated with MAPK, PPAR, Wnt/Notch signaling, calcium homeostasis, and metabolic processes (e.g., amino acid/fatty acid metabolism and arginine biosynthesis). (C–I) revealed the upregulated genes in metabolic, Toll-like receptor, vascular smooth muscle contraction, mTOR, cell adhesion molecules, MAPK, focal adhesion signaling pathways. (J–P) indicated the downregulated genes in metabolic; focal adhesion; ECM-receptor interaction; glycine, serine and threonine metabolism; fatty acid metabolism; and fatty acid metabolism, respectively. In addition, genes marked with red borders are potential hub genes. DEG, differentially expressed gene.

Utilizing KEGG pathways demonstrating statistical significance (p<0.05) and supported by existing literature evidence, we conducted pathway enrichment visualization of DEGs (up- and down-regulated) in 80D muscle tissue samples. As shown in Figure 3A and Supplement 4, the upregulated DEGs in 80D leg muscle were involved in many signaling pathways, including MAPK, VEGF, mTOR, lysosome, FoxO, endocytosis, peroxisome, adipocytokine, nitrogen metabolism, metabolic pathways, cell adhesion molecules, and vascular smooth muscle contraction signaling pathways. Moreover, the downregulated genes in 80D muscle were involved in signaling pathways, such as MAPK, PPAR, Notch, Wnt, calcium, adipocytokine, metabolic pathways; amino acids biosynthesis; fatty acid degradation; and arginine biosynthesis signaling pathways (Figure 3B and Supplement 5). In addition, the upregulated DEGs in metabolic, Toll-like receptor, vascular smooth muscle contraction, mTOR, cell adhesion molecules, MAPK, focal adhesion signaling pathways were visualized in Figures 3C–3I using the heatmaps, respectively. Also, the downregulated DEGs in metabolic; focal adhesion; ECM-receptor interaction; glycine, serine and threonine metabolism; valine, leucine and isoleucine degradation; fatty acid metabolism; and fatty acid metabolism were showed in Figures 3J–3P, respectively.

### PANTHER pathway analysis of the differentially expressed genes

**Figure 4:**
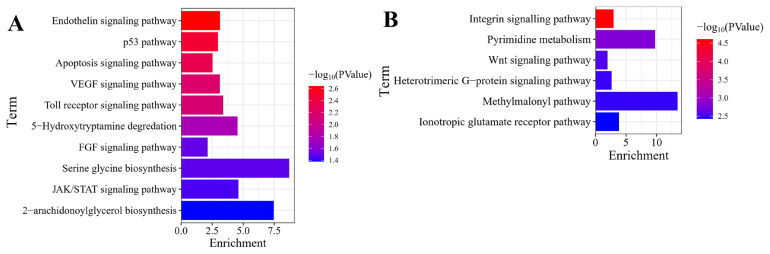
PANTHER pathway enrichment profiles of the DEGs. (A) and (B) depicted PANTHER pathway enrichments for the up- and down-regulated genes in 80D muscle, respectively. Upregulated genes predominantly mediated angiogenesis (VEGF), immune regulation (Jak-STAT/Toll/IL), and growth signaling (FGF/PDGF), while downregulated genes were enriched in cell adhesion (integrin), nucleotide metabolism (pyrimidine), and neural transmission (glutamate receptor) pathways. DEG, differentially expressed gene.

As illustrated in Figure 4A, the upregulated in 80D muscle were related to diverse pathways, including VEGF, Jak/STAT, FGF, Toll receptor, PDGF, p53, endothelin, apoptosis, interleukin, inflammation, T cell activation, B cell activation, and 2-arachidonoylglycerol biosynthesis signaling pathways. Furthermore, the downregulated in 80D muscle were associated with multiple pathways, such as integrin, pyrimidine metabolism, Wnt, Heterotrimeric G-protein, and ionotropic glutamate receptor signaling pathways. (Figure 4B).

### Protein classification for the differentially expressed genes

**Figure 5:**
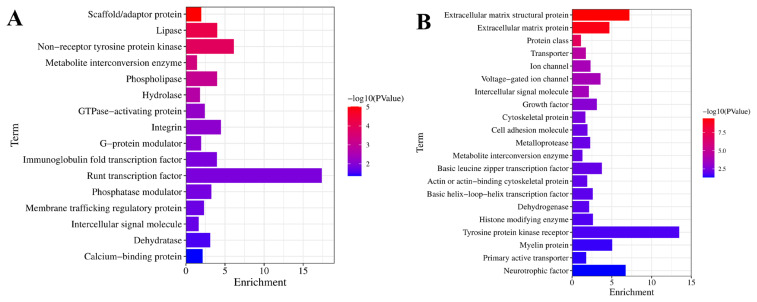
Protein class annotation of the DEGs. (A) and (B) represent protein classification for the up- and down-regulated genes in 80D muscle, respectively. Protein classification analysis of 80D muscle revealed upregulated enzymes (hydrolases/lipases) and signaling modulators (G-protein/integrin), while downregulated proteins comprised membrane transporters, structural components (ECM/cytoskeleton), and regulatory factors (transcription/kinases). DEG, differentially expressed gene.

As illustrated in Figure 5A, the upregulated in 80D muscle were related to diverse proteins, such as hydrolase, phospholipase, integrin, lipase, dehydratase, phosphatase modulator, scaffold/adaptor protein, G-protein modulator, and metabolite interconversion enzyme. Moreover, the downregulated in 80D muscle were related to a massive number of proteins, including transporter, ion channel, growth factor, cytoskeletal protein, intercellular signal molecule, extracellular matrix structural protein, cell adhesion molecule, metabolite interconversion enzyme, extracellular matrix protein, histone modifying enzyme, basic leucine zipper transcription factor, and tyrosine protein kinase receptor (Figure 5B).

### PPI network analysis

**Figure 6:**
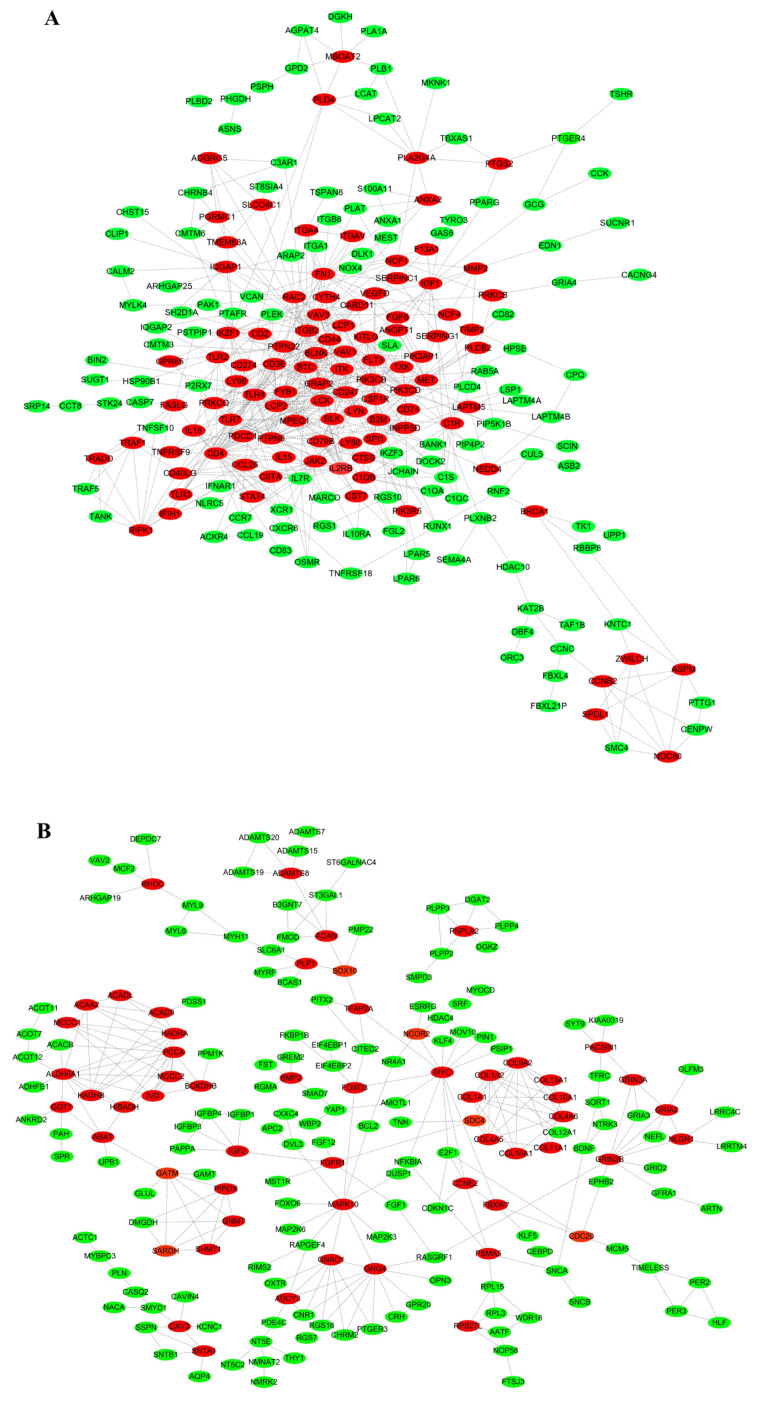
PPI network analysis for the DEGs. (A) and (B) indicated PPI network analysis for the up- and down-regulated genes in 80D muscle, respectively. Network analysis revealed key myogenesis genes: immunoregulators (*CD247/LCP2/LCK/CD3E*), lysosomal *CTSS*, and *CSF1R* were upregulated in 80D muscle, while ECM components (*COL9A2/COL1A2*) and metabolic enzymes (*ALDH6A1/ACADL*) were downregulated, indicating functional transitions in late muscle development. Genes labeled in red indicate potentially important genes, while those marked in green represent general genes. DEG, differentially expressed gene.

To elucidate key regulatory elements in Muscovy duck myogenesis, PPI networks were constructed for DEGs. Network topology analysis identified many critical myogenesis-related genes, with upregulated candidates in 80-day-old (80D) muscle tissue including immunoregulatory components (*CD247*, *LCP2*, *LCK*, *CD3E*), lysosomal protease (*CTSS*), and macrophage colony-stimulating factor receptor (*CSF1R*) (Figure 6A). Conversely, downregulated genes predominantly encoded extracellular matrix components (*COL9A2*, *COL1A2*) and metabolic enzymes (*ALDH6A1*, *ACADL*) (Figure 6B), suggesting distinct functional shifts during late-stage muscle development.

### Identification and functional characterization of hub genes

**Figure 7:**
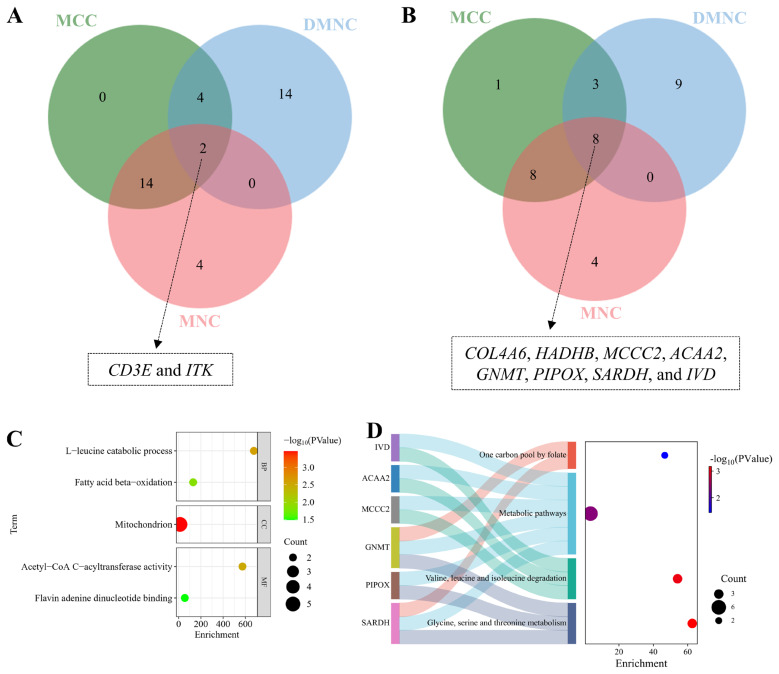
Identification and functional annotation of myogenesis-related hub genes in Muscovy duck. (A) and (B) depicted hub genes up- and down-regulated in 80D muscle, respectively. (C) and (D) reveal GO and KEGG enrichments for all hub genes, respectively. Consensus analysis identified immune-related hub genes (*CD3E, ITK*) among upregulated candidates, while downregulated hubs encoded core metabolic machinery including collagen IV (*COL4A6*), mitochondrial enzymes (*HADHB, MCCC2*), and amino acid regulators (*GNMT, PIPOX*). Functional enrichment demonstrated these hubs activate leucine catabolism, fatty acid β-oxidation, and key metabolic pathways: amino acid degradation, one-carbon metabolism, and folate cycling. GO, Gene Ontology.

Hub gene analysis was performed using CytoHubba with three topological algorithms (MCC, DMNC, and MNC). The consensus analysis revealed immune-related hub genes (*CD3E*, *ITK*) among upregulated candidates, while downregulated hubs comprised metabolic regulators including collagen type IV (*COL4A6*), mitochondrial enzymes (*HADHB*, *MCCC2*), and amino acid metabolism components (*GNMT*, *PIPOX*) (Figures 7A, 7B). Functional enrichment analyses demonstrated these hub genes’ involvement in leucine catabolism and fatty acid β-oxidation, along with participation in essential metabolic pathways: amino acid degradation, one-carbon metabolism, and folate cycle (Figure 7C). Notably, ITK was functionally annotated as a critical modulator of adaptive immunity, orchestrating T-cell and NKT-cell development and differentiation (Table 3), highlighting the intricate interplay between immune regulation and muscle development.

## DISCUSSION

Muscle development of Muscovy ducks represents a complex and biologically significant phenomenon, influenced by multiple determinants including but not limited to genetic breed, ontogenetic stage, sexual dimorphism, and nutritional parameters [15–18]. Females develop significantly larger but less dense myofibers than males by postnatal day 42, a disparity that progressively intensifies until day 70 [16]. This phenotypic variation correlates with distinct endocrine profiles—males maintain higher serum testosterone levels associated with superior growth metrics, while females show elevated estradiol concomitant with upregulated lipid metabolism markers (*CD36*, *CPT1A*) and suppressed AMPK phosphorylation [17]. Transcriptomic analyses reveal 1,118 DEGs, including core myogenic regulators (*MYLK4, KIT*) and sex-specific modulators (*TPM2*, *VCP*) that orchestrate muscle hypertrophy through AMPK signaling, focal adhesion, and calcium-dependent pathways [16]. Notably, nutritional interventions such as 2.5% earthworm hydrolysate supplementation demonstrate the plasticity of this system, significantly enhancing muscle yield independent of sexual dimorphism [18]. Our investigation systematically resolves this knowledge gap through transcriptomic profiling, revealing 1,683 DEGs and computationally prioritizing ten hub genes (*CD3E*, *ITK*, *COL4A6*, *HADHB*, *MCCC2*, *ACAA2*, *GNMT*, *PIPOX*, *SARDH*, and *IVD*) via integrated RNA-seq and protein-protein interaction network analyses. The functional convergence of these hub genes on L-leucine degradation and fatty acid β-oxidation pathways [19] underscores metabolic reprogramming as an evolutionary adaptation for rapid muscle growth - a finding that corroborates emerging paradigms in avian energy metabolism.

Our study reveals an unexpected role of *CD3E* and *ITK* in post-hatching muscle development, challenging existing paradigms in muscle biology. Three lines of evidence demonstrate their unconventional functions: First, *CD3E* appears to activate duck myoblasts through conserved inflammatory pathways similar to those documented in mammalian muscle repair [20–22]. Second, *ITK*’s well-known lymphocyte differentiation function [12] seems evolutionarily repurposed to regulate myofiber maturation during crucial developmental stages. Most notably, their persistent high expression in 80-day post-hatching muscles indicates these molecules may have adapted to sustain muscle hypertrophy under continuous metabolic stress. These findings significantly expand our understanding of myogenic regulation beyond traditional pathways. These findings fundamentally expand the conceptual framework of muscle development by demonstrating that immune-related genes constitute essential components of the growth regulatory network in commercial poultry breeds [23–25].

In addition, we identified several downregulated hub genes (*HADHB*, *ACAA2*, *GNMT*, *SARDH*, *IVD*, *MCCC2*, *PIPOX*, and *COL4A6*) that are functionally linked to critical metabolic pathways, including glycine/serine/threonine metabolism, branched-chain amino acid (BCAA) degradation, and folate-mediated one-carbon metabolism (Figure 7D). Notably, *HADHB* and *ACAA2*—key regulators of mitochondrial fatty acid β-oxidation—showed reduced expression in 80D muscles, consistent with a metabolic shift from lipid breakdown to anabolic processes during hypertrophy. This parallels observations in broiler chickens, where suppressed fatty acid oxidation accompanies rapid muscle growth [26]. Similarly, *GNMT* and *SARDH*, which modulate folate-dependent one-carbon metabolism, may influence epigenetic regulation of myogenic factors like *AKT* and *S6K1* to promote muscle development [27]. The decreased expression of *IVD* and *MCCC2*—enzymes involved in BCAA catabolism (Figures 3J, 3N)—further supports a preference for protein synthesis over amino acid degradation in mature muscle. Collectively, these metabolic adaptations align with the heightened anabolic demands of hypertrophic growth, facilitating sustained myofiber expansion [28].

The downregulation of *PIPOX* in 80D muscle (Figures 3J, 7D) was functionally linked to glycine, serine, and threonine metabolism. This aligns with established evidence that these pathways regulate skeletal muscle growth, fatty acid metabolism, and intramuscular fat deposition [29]. Concurrently, reduced *COL4A6* (collagen IV) expression in focal adhesion and ECM-receptor interaction pathways (Figures 3K, 3N) appears to regulate myoblast differentiation, muscle fiber formation, and muscle size [16,30–32]. Focal adhesion dynamics correlate with distinct developmental stages during myogenesis—including differentiation, fiber formation, and hypertrophic responses to mechanical loading—playing critical roles in muscle cell homeostasis [30]. Supporting this, Zhang et al [16] identified focal adhesion and ECM-receptor interaction pathway genes as determinants of breast muscle hypertrophy in Muscovy ducks, while Wu et al demonstrated their conserved role in driving skeletal muscle development across avian species (ducks and quails) [31,32]. These findings collectively highlight coordinated metabolic and structural adaptations underlying muscle development.

## CONCLUSION

While this study provides novel insights, several limitations warrant attention. First, the functional roles of prioritized hub genes (e.g., *CD3E*, *ITK*) in myogenesis remain hypothetical and require experimental validation through knockout or overexpression models. Second, the interplay between metabolic pathways needs mechanistic exploration to clarify their synergistic effects on myogenesis. Third, the study focused on transcriptomic changes, overlooking post-translational modifications or epigenetic regulation that may further modulate myogenesis. Future work should integrate multi-omics approaches (proteomics, metabolomics) and *in vivo* interventions to validate these targets and refine strategies for enhancing poultry meat quality.

## Figures and Tables

**Table 1:** Top 30 up-regulated genes in leg muscle in Muscovy duck at 80D compared to 1D

Gene symbol	Log_2_ FC	p-value	Description
*JCHAIN*	11.33	1.20×10^−5^	Joining chain of multimeric IgA and IgM
*METTL21C*	9.10	1.04×10^−25^	Methyltransferase 21C, AARS1 lysine
*ALDH1A1*	7.61	1.11×10^−2^	Aldehyde dehydrogenase 1 family member A1
*FAM3B*	6.89	1.61×10^−2^	FAM3 metabolism regulating signaling molecule B
*FLT3*	6.73	4.53×10^−5^	Fms related receptor tyrosine kinase 3
*CXCR6*	6.63	3.92×10^−7^	C-X-C motif chemokine receptor 6
*CD2*	6.58	1.49×10^−7^	CD2 molecule
*KCNG4*	6.42	1.65×10^−4^	Potassium voltage-gated channel modifier subfamily G member 4
*CD3E*	6.38	1.17×10^−6^	CD3e molecule
*TMEM273*	6.15	2.61×10^−4^	Transmembrane protein 273
*CCK*	5.95	3.33×10^−10^	Cholecystokinin
*LAG3*	5.89	6.39×10^−4^	Lymphocyte activating 3
*CD6*	5.77	3.21×10^−4^	CD6 molecule
*XCR1*	5.74	1.52×10^−3^	X-C motif chemokine receptor 1
*ACOD1*	5.71	2.39×10^−3^	Aconitate decarboxylase 1
*CD40LG*	5.67	8.48×10^−4^	CD40 ligand
*BCL2L15*	5.63	1.53×10^−3^	BCL2 like 15
*LIPI*	5.59	6.29×10^−3^	Lipase I
*PKIB*	5.42	1.56×10^−4^	CAMP-dependent protein kinase inhibitor beta
*GPR55*	5.28	1.62×10^−3^	G protein-coupled receptor 55
*CD8B*	5.28	3.27×10^−3^	CD8b molecule
*IRF4*	5.25	4.28×10^−3^	Interferon regulatory factor 4
*DHRS9*	5.24	2.90×10^−15^	Dehydrogenase/reductase 9
*TXK*	5.20	1.01×10^−3^	TXK tyrosine kinase
*CCL26*	5.06	6.62×10^−4^	C-C motif chemokine ligand 26
*CX3CR1*	4.97	1.36×10^−3^	C-X3-C motif chemokine receptor 1
*TFEC*	4.97	5.34×10^−4^	Transcription factor EC
*DEUP1*	4.94	1.14×10^−2^	Deuterosome assembly protein 1
*IL18*	4.78	2.49×10^−4^	Interleukin 18
*SLC2A6*	4.77	3.91×10^−4^	Solute carrier family 2 member 6

**Table 2:** Top 30 down-regulated genes in leg muscle in Muscovy duck at 80D compared to 1D

Gene symbol	Log_2_ FC	p-value	Description
*BD2*	−9.49	1.68×10^−3^	Antimicrobial peptide THP2
*CHRM2*	−7.84	1.35×10^−16^	Cholinergic receptor muscarinic 2
*DBX2*	−6.69	8.09×10^−5^	Developing brain homeobox 2
*GATM*	−6.23	1.89×10^−22^	Glycine amidinotransferase
*CALB1*	−6.02	6.07×10^−3^	Calbindin 1
*UROC1*	−6.00	1.19×10^−7^	Urocanate hydratase 1
*COL10A1*	−5.96	1.88×10^−5^	Collagen type X alpha 1 chain
*TLX1*	−5.88	1.81×10^−4^	T cell leukemia homeobox 1
*CRH*	−5.82	3.46×10^−3^	Corticotropin releasing hormone
*SRD5A2*	−5.80	1.38×10^−5^	Steroid 5 alpha-reductase 2
*GPR149*	−5.62	8.56×10^−5^	G protein-coupled receptor 149
*IYD*	−5.54	1.52×10^−3^	Iodotyrosine deiodinase
*ANGPTL7*	−5.53	4.37×10^−10^	Angiopoietin like 7
*CDCP2*	−5.50	1.21×10^−11^	CUB domain containing protein 2
*DYNLT4*	−5.40	3.65×10^−4^	Dynein light chain Tctex-type 4
*CLDN14*	−5.37	3.77×10^−5^	Claudin 14
*CLEC19A*	−5.28	4.18×10^−16^	C-type lectin domain containing 19A
*SMPD3*	−5.27	5.41×10^−3^	Sphingomyelin phosphodiesterase 3
*FBXO41*	−5.27	7.47×10^−4^	F-box protein 41
*FAM243A*	−5.23	7.23×10^−9^	Chromosome 1 C21orf140 homolog
*COL9A1*	−5.21	6.93×10^−7^	Collagen type IX alpha 1 chain
*SYT10*	−5.17	1.04×10^−3^	Synaptotagmin 10
*SGCZ*	−5.14	5.87×10^−14^	Sarcoglycan zeta
*IGF2BP1*	−5.05	6.15×10^−30^	Insulin like growth factor 2 mRNA binding protein 1
*TFAP2A*	−5.03	2.08×10^−2^	Transcription factor AP-2 alpha
*LEAP2*	−5.03	1.09×10^−3^	Liver enriched antimicrobial peptide 2
*MFSD2A*	−5.02	1.47×10^−14^	Major facilitator superfamily domain containing 2A
*AXDND1*	−4.97	3.86×10^−4^	Axonemal dynein light chain domain containing 1
*CHRNA10*	−4.97	6.27×10^−3^	Cholinergic receptor nicotinic alpha 10 subunit
*ACTC1*	−4.90	9.81×10^−9^	Actin alpha cardiac muscle 1

**Table 3:** Hub genes for myogenesis of Muscovy duck post-hatching

Genes symbol	Full name	Functions
*CD3E*	CD3e molecule	The protein encoded by this gene plays an important role in coupling antigen recognition to several intracellular signal-transduction pathways. The genes encoding the epsilon, gamma and delta polypeptides are located in the same cluster on chromosome 11.
*ITK*	Tyrosine-protein kinase	*ITK* plays an essential role in regulation of the adaptive immune response. It regulates the development, function and differentiation of conventional T-cells and nonconventional NKT-cells.
*COL4A6*	Collagen type IV alpha 6 chain	*COL4A6* encodes one of the six subunits of type IV collagen, the major structural component of basement membranes. *COL4A6* is organized in a head-to-head conformation with another type IV collagen gene, alpha 5 type IV collagen.
*HADHB*	Hydroxyacyl-CoA dehydrogenase trifunctional multienzyme complex subunit beta	This gene encodes the beta subunit of the mitochondrial trifunctional protein, which catalyzes the last three steps of mitochondrial beta-oxidation of long chain fatty acids. The mitochondrial membrane-bound heterocomplex is composed of four alpha and four beta subunits.
*MCCC2*	Methylcrotonoyl-CoA carboxylase 2	*PCCA* encodes the small subunit of 3-methylcrotonyl-CoA carboxylase. This enzyme functions as a heterodimer and catalyzes the carboxylation of 3-methylcrotonyl-CoA to form 3-methylglutaconyl-CoA.
*ACAA2*	Acetyl-CoA acyltransferase 2	The encoded protein catalyzes the last step of the mitochondrial fatty acid beta-oxidation spiral. Unlike most mitochondrial matrix proteins, it contains a non-cleavable amino-terminal targeting signal.
*GNMT*	Glycine N-methyltransferase	The protein encoded by *GNMT* located in the cytoplasm, acts as a homotetramer and an enzyme that catalyzes the conversion of S-adenosyl-L-methionine to S-adenosyl-L-homocysteine and sarcosine.
*PIPOX*	Pipecolic acid and sarcosine oxidase	*PIPOX* enables L-pipecolate oxidase activity and sarcosine oxidase activity. It is involved in L-lysine catabolic process to acetyl-CoA via L-pipecolate. Located in peroxisome.
*SARDH*	Sarcosine dehydrogenase	*SARDH* encodes an enzyme localized to the mitochondrial matrix which catalyzes the oxidative demethylation of sarcosine. This enzyme catalyzes a reaction resulting in the formation of sarcosine.
*IVD*	Isovaleryl-CoA dehydrogenase	Isovaleryl-CoA dehydrogenase (IVD) is a mitochondrial matrix enzyme that catalyzes the third step in leucine catabolism. The genetic deficiency of IVD results in an accumulation of isovaleric acid, which is toxic to the central nervous system and leads to isovaleric acidemia.

## SUPPLEMENTARY MATERIAL

**Supplement 1.** The DEGs identified in animal muscle at 1D and 80D.

**Supplement 2.** GO enrichment analysis for up-regulated genes in 80D.

**Supplement 3.**GO enrichment analysis for down-regulated genes in 80D.

**Supplement 4.** KEGG enrichment analysis for up-regulated genes in 80D.

**Supplement 5.** KEGG enrichment analysis for down-regulated genes in 80D.
