# Supplementary material for: Dynamic transcriptomic landscape of myogenesis in Muscovy ducks (Cairina moschata): integrative analysis of hub genes post-hatching
Source: Anim Biosci. 2025 Aug 12;39(1):250159. doi: 10.5713/ab.25.0159 (PMC12754469; doi:10.5713/ab.25.0159)
Supplement: Supplementary file 1 [file ab-25-0159-Supplementary-1.pdf]

Supplement 1. The DEGs identified in animal muscle at 1D and 80D

Gene symbol	log2FC	P Value	Full name
BD2	-9.49	1.68E-03	Antimicrobial peptide THP2
CHRM2	-7.84	1.35E-16	Cholinergic receptor muscarinic 2
DBX2	-6.69	8.09E-05	Developing brain homeobox 2
GATM	-6.23	1.89E-22	Glycine amidinotransferase
CALB1	-6.02	6.07E-03	Calbindin 1
UROC1	-6.00	1.19E-07	Urocanate hydratase 1
COL10A1	-5.96	1.88E-05	Collagen type X alpha 1 chain
TLX1	-5.88	1.81E-04	T cell leukemia homeobox 1
CRH	-5.82	3.46E-03	Corticotropin releasing hormone
SRD5A2	-5.80	1.38E-05	Steroid 5 alpha-reductase 2
GPR149	-5.62	8.56E-05	G protein-coupled receptor 149
IYD	-5.54	1.52E-03	Iodotyrosine deiodinase
ANGPTL7	-5.53	4.37E-10	Angiopoietin like 7
CDCP2	-5.50	1.21E-11	CUB domain containing protein 2
DYNLT4	-5.40	3.65E-04	Dynein light chain Tctex-type 4
CLDN14	-5.37	3.77E-05	Claudin 14
CLEC19A	-5.28	4.18E-16	C-type lectin domain containing 19A
SMPD3	-5.27	5.41E-03	Sphingomyelin phosphodiesterase 3
FBXO41	-5.27	7.47E-04	F-box protein 41
FAM243A	-5.23	7.23E-09	Chromosome 1 C21orf140 homolog
COL9A1	-5.21	6.93E-07	Collagen type IX alpha 1 chain
SYT10	-5.17	1.04E-03	Synaptotagmin 10
SGCZ	-5.14	5.87E-14	Sarcoglycan zeta
IGF2BP1	-5.05	6.15E-30	Insulin like growth factor 2 mRNA binding protein 1
TFAP2A	-5.03	2.08E-02	Transcription factor AP-2 alpha
LEAP2	-5.03	1.09E-03	Liver enriched antimicrobial peptide 2
MFSD2A	-5.02	1.47E-14	Major facilitator superfamily domain containing 2A
AXDND1	-4.97	3.86E-04	Axonemal dynein light chain domain containing 1
CHRNA10	-4.97	6.27E-03	Cholinergic receptor nicotinic alpha 10 subunit
ACTC1	-4.90	9.81E-09	Actin alpha cardiac muscle 1
GIPC3	-4.89	1.44E-02	GIPC PDZ domain containing family member 3
KCNIP2	-4.87	9.19E-03	Potassium voltage-gated channel interacting protein 2
BRS3	-4.70	2.84E-02	Bombesin receptor subtype 3
CCN3	-4.69	9.68E-19	Cellular communication network factor 3
ABHD8	-4.68	3.22E-03	Abhydrolase domain containing 8
LHFPL5	-4.67	5.42E-14	LHFPL tetraspan subfamily member 5
SOST	-4.65	3.78E-02	Sclerostin
GUCA1B	-4.60	3.18E-03	Guanylate cyclase activator 1B

DMBX1	-4.54	8.04E-03	Diencephalon/mesencephalon homeobox 1
CACNG5	-4.53	2.38E-02	Calcium voltage-gated channel auxiliary subunit gamma 5
SLC29A4	-4.52	1.10E-06	Solute carrier family 29 member 4
SYT3	-4.49	5.67E-03	Synaptotagmin 3
PPDPFL	-4.49	1.24E-07	Pancreatic progenitor cell differentiation and proliferation factor like
LRIT2	-4.48	6.66E-04	Leucine rich repeat, Ig-like and transmembrane domains 2
FAM222A	-4.42	8.16E-03	Family with sequence similarity 222 member A
EMX2	-4.38	2.27E-04	Empty spiracles homeobox 2
TMEM132D	-4.37	4.74E-02	Transmembrane protein 132D
IGF2BP3	-4.30	2.40E-22	Insulin like growth factor 2 mRNA binding protein 3
PIPOX	-4.26	7.85E-10	Pipecolic acid and sarcosine oxidase
SLC6A14	-4.25	4.19E-02	Solute carrier family 6 member 14
IGSF10	-4.24	2.70E-12	Immunoglobulin superfamily member 10
UBAP1L	-4.23	1.89E-02	Ubiquitin associated protein 1 like
MSH5	-4.23	4.02E-03	MutS homolog 5
TAS1R3	-4.19	1.98E-02	Taste 1 receptor member 3
POU3F3	-4.19	1.17E-02	POU class 3 homeobox 3
MAB21L2	-4.14	1.03E-03	Mab-21 like 2
CHADL	-4.13	1.79E-02	Chondroadherin like
GPR61	-4.12	1.40E-02	G protein-coupled receptor 61
BDNF	-4.11	1.21E-04	Brain derived neurotrophic factor
TNFRSF13B	-4.10	8.99E-07	TNF receptor superfamily member 13B
PPFIA2	-4.09	2.09E-02	PTPRF interacting protein alpha 2
KCNH6	-4.07	1.09E-11	Potassium voltage-gated channel subfamily H member 6
COL4A5	-4.03	2.89E-21	Collagen type IV alpha 5 chain
MYO15B	-3.99	2.81E-02	Myosin XVB
ARMC12	-3.94	2.61E-02	Armadillo repeat containing 12
MASP1	-3.92	2.71E-02	MBL associated serine protease 1
NKX2-3	-3.91	2.47E-02	NK2 homeobox 3
LRIT3	-3.89	7.10E-03	Leucine rich repeat, Ig-like and transmembrane domains 3
ERP27	-3.87	4.79E-03	Endoplasmic reticulum protein 27
PCDH10	-3.81	1.25E-08	Protocadherin 10
DLX2	-3.80	3.45E-02	Distal-less homeobox 2
FKBP5	-3.80	8.33E-14	FKBP prolyl isomerase 5
LAD1	-3.80	4.68E-02	Ladinin 1
FOXO6	-3.75	6.66E-37	Forkhead box O6
RTN4RL2	-3.70	2.53E-06	Reticulon 4 receptor like 2

FOXD3	-3.69	2.00E-06	Forkhead box D3
GNG4	-3.67	5.72E-08	G protein subunit gamma 4
FAXDC2	-3.67	2.23E-05	Fatty acid hydroxylase domain containing 2
PDZD7	-3.65	1.63E-09	PDZ domain containing 7
STC2	-3.65	8.51E-15	Stanniocalcin 2
HPX	-3.64	2.02E-03	Hemopexin
PEX5L	-3.58	2.80E-07	Peroxisomal biogenesis factor 5 like
HMGA2	-3.57	3.29E-05	High mobility group AT-hook 2
SPP1	-3.53	5.43E-05	Secreted phosphoprotein 1
PLPP4	-3.51	1.03E-04	Phospholipid phosphatase 4
STC1	-3.49	5.07E-07	Stanniocalcin 1
COL11A1	-3.48	3.98E-10	Collagen type XI alpha 1 chain
OPN3	-3.48	1.89E-05	Opsin 3
IGSF21	-3.47	3.19E-06	Immunoglobin superfamily member 21
MUC2	-3.46	6.00E-04	Mucin 2, oligomeric mucus/gel-forming
FMOD	-3.46	1.06E-05	Fibromodulin
SLC16A8	-3.44	2.10E-02	Solute carrier family 16 member 8
DGAT2	-3.44	2.94E-06	Diacylglycerol O-acyltransferase 2
RNF43	-3.44	6.00E-03	Ring finger protein 43
AMN	-3.42	1.03E-02	Amnion associated transmembrane protein
FUT9	-3.41	2.08E-03	Fucosyltransferase 9
TNS4	-3.41	4.07E-04	Tensin 4
LOXL4	-3.41	1.06E-02	Lysyl oxidase like 4
CILP2	-3.41	1.22E-09	Cartilage intermediate layer protein 2
LRRC73	-3.40	1.82E-03	Leucine rich repeat containing 73
COL4A6	-3.38	1.20E-10	Collagen type IV alpha 6 chain
GABRA4	-3.35	1.81E-14	Gamma-aminobutyric acid type A receptor subunit alpha4
WFIKKN2	-3.34	1.81E-04	WAP, follistatin/kazal, immunoglobulin, kunitz and netrin domain containing 2
CXCL14	-3.34	1.17E-04	C-X-C motif chemokine ligand 14
PLK3	-3.33	9.28E-08	Polo like kinase 3
NTRK3	-3.33	6.49E-17	Neurotrophic receptor tyrosine kinase 3
SHOX2	-3.31	1.58E-05	Short stature homeobox 2
GASK1A	-3.29	1.01E-05	Golgi associated kinase 1A
ELOVL3	-3.29	1.87E-02	ELOVL fatty acid elongase 3
C1H12orf75	-3.29	1.19E-04	Chromosome 1 C12orf75 homolog
ST8SIA2	-3.28	2.31E-05	ST8 alpha-N-acetyl-neuraminide alpha-2,8-sialyltransferase 2
SNAI3	-3.27	7.16E-06	Snail family transcriptional repressor 3
NEU4	-3.22	3.32E-02	Neuraminidase 4
EFNA3	-3.21	3.38E-02	Ephrin A3

RSAD2	-3.20	1.27E-03	Radical S-adenosyl methionine domain containing 2
FSCN2	-3.20	5.21E-07	Fascin actin-bundling protein 2, retinal
USH1G	-3.19	3.79E-03	USH1 protein network component sans
TNMD	-3.17	4.25E-05	Tenomodulin
GPR139	-3.16	7.01E-03	G protein-coupled receptor 139
ME3	-3.16	2.83E-23	Malic enzyme 3
ERBB3	-3.14	5.34E-05	Erb-b2 receptor tyrosine kinase 3
ATP7B	-3.14	7.53E-05	ATPase copper transporting beta
DOK5	-3.13	5.71E-16	Docking protein 5
SLC26A9	-3.13	3.29E-02	Solute carrier family 26 member 9
IGFBP1	-3.13	1.12E-03	Insulin like growth factor binding protein 1
NELL1	-3.10	2.96E-02	Neural EGFL like 1
PHF19	-3.10	4.26E-16	PHD finger protein 19
MAPK10	-3.09	2.95E-02	Mitogen-activated protein kinase 10
PNOC	-3.08	1.68E-04	Prepronociceptin
CASKIN1	-3.07	2.69E-03	CASK interacting protein 1
MAP7D2	-3.07	4.29E-02	MAP7 domain containing 2
NFASC	-3.06	6.21E-05	Neurofascin
RPS6KL1	-3.05	2.24E-05	Ribosomal protein S6 kinase like 1
TMEM151B	-3.03	2.51E-04	Transmembrane protein 151B
ZNF385C	-3.02	2.36E-05	Zinc finger protein 385C
CCDC194	-3.02	1.96E-02	Coiled-coil domain containing 194
PCDH17	-3.02	8.45E-07	Protocadherin 17
GRIA2	-3.01	4.79E-02	Glutamate ionotropic receptor AMPA type subunit 2
MTMR7	-3.01	1.84E-04	Myotubularin related protein 7
HCN1	-3.00	6.70E-03	Hyperpolarization activated cyclic nucleotide gated potassium channel 1
GAS2	-2.99	2.70E-03	Growth arrest specific 2
CYTL1	-2.98	8.83E-05	Cytokine like 1
PITX2	-2.98	6.35E-06	Paired like homeodomain 2
KLF9	-2.98	4.64E-38	Kruppel like factor 9
GRIN3A	-2.97	3.44E-11	Glutamate ionotropic receptor NMDA type subunit 3A
PRELID3A	-2.96	2.10E-10	PRELI domain containing 3A
ZNF469	-2.96	7.68E-06	Zinc finger protein 469
ELN	-2.95	3.27E-07	Elastin
SCRT2	-2.94	2.40E-02	Scratch family transcriptional repressor 2
IRX5	-2.92	3.00E-04	Iroquois homeobox 5
NKX6-3	-2.92	2.23E-02	NK6 homeobox 3
CNNM1	-2.92	3.88E-04	Cyclin and CBS domain divalent metal cation transport mediator 1

ADAMTS8	-2.91	1.53E-07	ADAM metallopeptidase with thrombospondin type 1 motif 8
PLPPR1	-2.89	3.91E-13	Phospholipid phosphatase related 1
MLN	-2.89	6.84E-05	Motilin
TENM1	-2.87	4.85E-03	Teneurin transmembrane protein 1
GRIN2B	-2.86	9.88E-07	Glutamate ionotropic receptor NMDA type subunit 2B
ACTG2	-2.85	2.25E-04	Actin gamma 2, smooth muscle
IGF2BP2	-2.84	2.08E-10	Insulin like growth factor 2 mRNA binding protein 2
CPZ	-2.83	2.85E-03	Carboxypeptidase Z
SLC5A11	-2.82	3.02E-02	Solute carrier family 5 member 11
CRTAC1	-2.81	2.50E-05	Cartilage acidic protein 1
APOA1	-2.79	1.30E-06	Apolipoprotein A1
RHOC	-2.79	9.62E-06	Ras homolog family member C
GREM2	-2.79	4.25E-08	Gremlin 2, DAN family BMP antagonist
GAS2L2	-2.78	3.34E-03	Growth arrest specific 2 like 2
MYO3B	-2.78	2.56E-02	Myosin IIIB
GAL3ST1	-2.77	4.47E-03	Galactose-3-O-sulfotransferase 1
GLUL	-2.77	7.48E-10	Glutamate-ammonia ligase
MTNR1A	-2.76	4.17E-03	Melatonin receptor 1A
ANKRD2	-2.75	2.89E-05	Ankyrin repeat domain 2
COL1A1	-2.75	9.77E-06	Collagen type I alpha 1 chain
CCDC69	-2.73	2.67E-05	Coiled-coil domain containing 69
PAQR9	-2.73	3.59E-14	Progestin and adipoQ receptor family member 9
TNN	-2.72	8.19E-03	Tenascin N
RAPGEF4	-2.72	1.39E-04	Rap guanine nucleotide exchange factor 4
NMNAT2	-2.71	2.01E-03	Nicotinamide nucleotide adenylyltransferase 2
IQCA1	-2.70	7.53E-06	IQ motif containing with AAA domain 1
IQSEC3	-2.70	5.41E-05	IQ motif and Sec7 domain ArfGEF 3
APOD	-2.69	2.59E-04	Apolipoprotein D
ARTN	-2.69	1.37E-04	Artemin
USH1C	-2.69	7.10E-05	USH1 protein network component harmonin
LRRTM4	-2.69	7.92E-04	Leucine rich repeat transmembrane neuronal 4
DNAH3	-2.68	3.57E-06	Dynein axonemal heavy chain 3
POU3F1	-2.68	7.77E-04	POU class 3 homeobox 1
CPS1	-2.67	1.32E-07	Carbamoyl-phosphate synthase 1
PGC	-2.66	3.50E-03	Progastricsin
SCX	-2.66	4.46E-03	Scleraxis bHLH transcription factor
RASSF7	-2.66	4.38E-04	Ras association domain family member 7
FGFRL1	-2.65	8.12E-11	Fibroblast growth factor receptor like 1
CRACD	-2.65	3.59E-03	Capping protein inhibiting regulator of actin dynamics

SLC4A7	-2.65	6.30E-06	Solute carrier family 4 member 7
MYBPH	-2.64	3.16E-03	Myosin binding protein H
CAMKV	-2.63	7.31E-03	CaM kinase like vesicle associated
ISM1	-2.63	1.90E-07	Isthmin 1
DYNC1I1	-2.63	2.01E-03	Dynein cytoplasmic 1 intermediate chain 1
GRM7	-2.63	2.35E-03	Glutamate metabotropic receptor 7
SHMT1	-2.63	4.63E-07	Serine hydroxymethyltransferase 1
CLCN2	-2.63	6.69E-03	Chloride voltage-gated channel 2
KDF1	-2.61	3.72E-05	Keratinocyte differentiation factor 1
ZNF488	-2.61	1.18E-02	Zinc finger protein 488
SORL1	-2.60	4.84E-27	Sortilin related receptor 1
CNTFR	-2.60	9.48E-04	Ciliary neurotrophic factor receptor
PLEKHH1	-2.58	1.04E-02	Pleckstrin homology, MyTH4 and FERM domain containing H1
SDK2	-2.58	1.27E-03	Sidekick cell adhesion molecule 2
OLFM3	-2.57	2.48E-02	Olfactomedin 3
RGS7	-2.55	2.78E-03	Regulator of G protein signaling 7
AMER3	-2.53	4.35E-02	APC membrane recruitment protein 3
KCNE1	-2.53	1.34E-02	Potassium voltage-gated channel subfamily E regulatory subunit 1
ADAMTSL5	-2.52	3.27E-06	ADAMTS like 5
NWD2	-2.50	2.27E-03	NACHT and WD repeat domain containing 2
GNAO1	-2.50	4.65E-05	G protein subunit alpha o1
WNT2B	-2.50	9.84E-04	Wnt family member 2B
LEPR	-2.49	3.09E-09	Leptin receptor
WNT2	-2.49	1.64E-05	Wnt family member 2
DLGAP2	-2.49	4.70E-02	DLG associated protein 2
FBN2	-2.48	2.86E-07	Fibrillin 2
SLIT1	-2.48	2.73E-03	Slit guidance ligand 1
ADM2	-2.47	2.30E-02	Adrenomedullin 2
ARL13A	-2.47	4.23E-02	ADP ribosylation factor like GTPase 13A
CEBPD	-2.46	4.72E-04	CCAAT enhancer binding protein delta
CACNA1E	-2.46	2.06E-04	Calcium voltage-gated channel subunit alpha1 E
SLC6A1	-2.46	3.23E-02	Solute carrier family 6 member 1
MCOLN3	-2.45	2.76E-05	Mucolipin TRP cation channel 3
SYT9	-2.44	5.77E-04	Synaptotagmin 9
SOX10	-2.43	3.38E-04	SRY-box transcription factor 10
GAMT	-2.43	2.56E-07	Guanidinoacetate N-methyltransferase
DMRT2	-2.43	4.98E-04	Doublesex and mab-3 related transcription factor 2
LAMA1	-2.42	4.61E-02	Laminin subunit alpha 1
TMEM74	-2.42	1.10E-04	Transmembrane protein 74
CLDN19	-2.41	4.87E-04	Claudin 19

AJM1	-2.41	9.11E-03	Apical junction component 1 homolog
AATK	-2.40	9.06E-04	Apoptosis associated tyrosine kinase
KAZALD1	-2.40	3.70E-04	Kazal type serine peptidase inhibitor domain 1
COL16A1	-2.40	7.02E-06	Collagen type XVI alpha 1 chain
PPP1R3B	-2.39	5.33E-10	Protein phosphatase 1 regulatory subunit 3B
FKBP1B	-2.38	1.78E-05	FKBP prolyl isomerase 1B
SCN3B	-2.36	7.34E-04	Sodium voltage-gated channel beta subunit 3
SLC12A5	-2.34	2.66E-04	Solute carrier family 12 member 5
HS6ST3	-2.34	1.27E-06	Heparan sulfate 6-O-sulfotransferase 3
XKR6	-2.34	2.48E-08	XK related 6
SLC16A12	-2.34	1.48E-07	Solute carrier family 16 member 12
RRAD	-2.34	3.78E-03	RRAD, Ras related glycolysis inhibitor and calcium channel regulator
PACSIN1	-2.33	9.34E-03	Protein kinase C and casein kinase substrate in neurons 1
GLDN	-2.32	2.50E-03	Gliomedin
PDK1	-2.31	6.57E-06	Pyruvate dehydrogenase kinase 1
CCN5	-2.31	4.19E-05	Cellular communication network factor 5
KCNE3	-2.30	8.34E-05	Potassium voltage-gated channel subfamily E regulatory subunit 3
GLIS3	-2.30	6.56E-06	GLIS family zinc finger 3
S100A1	-2.30	1.12E-05	S100 calcium binding protein A1
SHANK2	-2.29	8.41E-03	SH3 and multiple ankyrin repeat domains 2
MYCL	-2.29	1.51E-02	MYCL proto-oncogene, bHLH transcription factor
CORIN	-2.29	4.98E-07	Corin, serine peptidase
MIDN	-2.27	2.35E-03	Midnolin
PDE4C	-2.27	2.79E-03	Phosphodiesterase 4C
STOX1	-2.26	1.06E-04	Storkhead box 1
PPM1K	-2.26	1.04E-05	Protein phosphatase, Mg2+/Mn2+ dependent 1K
TG	-2.26	1.30E-05	Thyroglobulin
PAH	-2.25	3.53E-03	Phenylalanine hydroxylase
MYBPC3	-2.25	1.38E-02	Myosin binding protein C3
FABP3	-2.24	2.05E-04	Fatty acid binding protein 3
TMEM205	-2.24	7.90E-04	Transmembrane protein 205
TMIGD1	-2.24	4.91E-03	Transmembrane and immunoglobulin domain containing 1
RCAN1	-2.23	1.50E-04	Regulator of calcineurin 1
HAND2	-2.22	3.60E-03	Heart and neural crest derivatives expressed 2
ACOT12	-2.22	1.90E-02	Acyl-CoA thioesterase 12
RSPO3	-2.22	5.19E-03	R-spondin 3
REEP6	-2.21	2.00E-05	Receptor accessory protein 6
RASGEF1B	-2.20	2.20E-05	RasGEF domain family member 1B

GJD2	-2.20	6.58E-03	Gap junction protein delta 2
UBTD1	-2.19	5.24E-06	Ubiquitin domain containing 1
VAT1L	-2.19	8.53E-03	Vesicle amine transport 1 like
IGSF11	-2.19	4.70E-03	Immunoglobulin superfamily member 11
METTL27	-2.18	6.42E-04	Methyltransferase like 27
TMEM120A	-2.18	1.21E-05	Transmembrane protein 120A
SNCB	-2.17	7.07E-03	Synuclein beta
DOC2B	-2.17	2.59E-04	Double C2 domain beta
B3GNT7	-2.17	1.54E-02	UDP-GlcNAc:betaGal beta-1,3-N-acetylglucosaminyltransferase 7
TMEM98	-2.15	1.94E-09	Transmembrane protein 98
ASTN2	-2.15	5.97E-04	Astrotactin 2
COL2A1	-2.15	2.32E-02	Collagen type II alpha 1 chain
LRG1	-2.15	6.67E-03	Leucine rich alpha-2-glycoprotein 1
NLGN1	-2.14	1.74E-02	Neuroligin 1
PLP1	-2.14	1.51E-02	Proteolipid protein 1
ENAH	-2.14	1.91E-04	ENAH actin regulator
APOH	-2.13	4.74E-02	Apolipoprotein H
GRID2	-2.13	4.81E-02	Glutamate ionotropic receptor delta type subunit 2
DRP2	-2.12	3.80E-02	Dystrophin related protein 2
SOX11	-2.12	1.20E-07	SRY-box transcription factor 11
KLHL32	-2.10	4.54E-04	Kelch like family member 32
SFRP5	-2.10	4.36E-02	Secreted frizzled related protein 5
TEKT3	-2.09	5.44E-03	Tektin 3
SMTNL1	-2.09	4.71E-05	Smoothelin like 1
COL9A2	-2.08	2.69E-03	Collagen type IX alpha 2 chain
CFAP69	-2.08	4.35E-04	Cilia and flagella associated protein 69
CAV3	-2.08	8.92E-07	Caveolin 3
RNF157	-2.08	2.06E-03	Ring finger protein 157
CISH	-2.08	5.54E-09	Cytokine inducible SH2 containing protein
BAIAP2L2	-2.07	1.17E-02	BAR/IMD domain containing adaptor protein 2 like 2
KIAA0319	-2.07	4.75E-05	KIAA0319 ortholog
CDH7	-2.07	1.67E-02	Cadherin 7
PAPPA	-2.07	1.71E-10	Pappalysin 1
NECAB2	-2.06	1.72E-07	N-terminal EF-hand calcium binding protein 2
PCSK2	-2.06	1.43E-15	Proprotein convertase subtilisin/kexin type 2
PMM1	-2.06	4.99E-04	Phosphomannomutase 1
BDH1	-2.06	2.69E-03	3-hydroxybutyrate dehydrogenase 1
CNR1	-2.04	6.01E-04	Cannabinoid receptor 1
GDAP1L1	-2.04	1.81E-03	Ganglioside induced differentiation associated protein 1 like 1
COL4A4	-2.03	9.64E-03	Collagen type IV alpha 4 chain

CSPG5	-2.03	2.86E-02	Chondroitin sulfate proteoglycan 5
LINGO3	-2.03	6.90E-04	Leucine rich repeat and Ig domain containing 3
ACAN	-2.03	4.08E-02	Aggrecan
ICA1	-2.02	1.15E-07	Islet cell autoantigen 1
HK2	-2.02	7.71E-06	Hexokinase 2
THBS4	-2.02	9.23E-05	Thrombospondin 4
FBXL22	-2.02	2.17E-06	F-box and leucine rich repeat protein 22
HES1	-2.01	3.90E-03	Hes family bHLH transcription factor 1
DEPDC7	-2.01	2.31E-03	DEP domain containing 7
FSTL5	-2.01	1.27E-02	Follistatin like 5
TRMT9B	-2.01	2.60E-02	TRNA methyltransferase 9B (putative)
SCN5A	-2.00	4.15E-04	Sodium voltage-gated channel alpha subunit 5
SHROOM3	-2.00	2.03E-03	Shroom family member 3
HOGA1	-1.99	3.50E-03	4-hydroxy-2-oxoglutarate aldolase 1
QSOX1	-1.99	6.30E-10	Quiescin sulfhydryl oxidase 1
PPARGC1B	-1.99	1.27E-05	PPARG coactivator 1 beta
ANKRD9	-1.99	6.81E-04	Ankyrin repeat domain 9
GPR148	-1.99	3.38E-02	G protein-coupled receptor 148
NT5E	-1.99	2.21E-02	5'-nucleotidase ecto
UNC80	-1.98	1.34E-04	Unc-80 homolog, NALCN channel complex subunit
FOXJ1	-1.98	1.73E-03	Forkhead box J1
KCNG2	-1.98	5.59E-04	Potassium voltage-gated channel modifier subfamily G member 2
PSTPIP2	-1.98	1.13E-02	Proline-serine-threonine phosphatase interacting protein 2
NECTIN3	-1.98	9.05E-11	Nectin cell adhesion molecule 3
KCNMA1	-1.98	8.48E-07	Potassium calcium-activated channel subfamily M alpha 1
PCP4	-1.98	1.16E-02	Purkinje cell protein 4
FA2H	-1.97	2.35E-02	Fatty acid 2-hydroxylase
NR4A1	-1.97	1.59E-02	Nuclear receptor subfamily 4 group A member 1
SLC6A9	-1.97	2.27E-04	Solute carrier family 6 member 9
SLC2A9	-1.96	1.05E-02	Solute carrier family 2 member 9
SLC19A2	-1.96	3.90E-04	Solute carrier family 19 member 2
B4GALNT4	-1.96	5.16E-04	Beta-1,4-N-acetyl-galactosaminyltransferase 4
PARD6A	-1.95	6.26E-03	Par-6 family cell polarity regulator alpha
PRX	-1.95	4.49E-02	Periaxin
EML6	-1.95	1.97E-02	EMAP like 6
SYN3	-1.95	2.69E-02	Synapsin III
HEBP2	-1.95	3.19E-03	Heme binding protein 2
C28H17orf113	-1.94	3.48E-05	Chromosome 28 C17orf113 homolog
MLPH	-1.94	4.44E-03	Melanophilin

FRAS1	-1.93	2.88E-04	Fraser extracellular matrix complex subunit 1
LAMB3	-1.93	3.75E-04	Laminin subunit beta 3
SNCA	-1.93	1.12E-02	Synuclein alpha
LRRC38	-1.92	6.01E-03	Leucine rich repeat containing 38
MAP2K6	-1.92	1.09E-04	Mitogen-activated protein kinase kinase 6
CCNE2	-1.92	2.07E-07	Cyclin E2
CD320	-1.92	1.60E-04	CD320 molecule
ACOT11	-1.92	3.29E-05	Acyl-CoA thioesterase 11
GNMT	-1.91	1.18E-02	Glycine N-methyltransferase
SLC22A3	-1.90	1.37E-02	Solute carrier family 22 member 3
MATN4	-1.90	4.23E-02	Matrilin 4
GK	-1.90	7.87E-03	Glycerol kinase
FIBIN	-1.89	2.09E-03	Fin bud initiation factor homolog
PHF5A	-1.89	9.90E-03	PHD finger protein 5A
PPP1R1C	-1.89	6.09E-03	Protein phosphatase 1 regulatory inhibitor subunit 1C
ARHGAP19	-1.89	1.20E-04	Rho GTPase activating protein 19
PHACTR3	-1.89	5.35E-03	Phosphatase and actin regulator 3
KIF1A	-1.89	3.18E-02	Kinesin family member 1A
PER3	-1.89	4.58E-10	Period circadian regulator 3
WBP2	-1.88	2.16E-09	WW domain binding protein 2
MYL9	-1.88	1.47E-03	Myosin light chain 9
TAF4B	-1.88	1.23E-02	TATA-box binding protein associated factor 4b
COL22A1	-1.87	1.05E-03	Collagen type XXII alpha 1 chain
BCAS1	-1.87	8.66E-05	Brain enriched myelin associated protein 1
SNTB1	-1.87	1.00E-08	Syntrophin beta 1
EYS	-1.87	1.60E-06	Eyes shut homolog
DIO3	-1.86	2.13E-03	Iodothyronine deiodinase 3
SLC52A3	-1.86	1.24E-02	Solute carrier family 52 member 3
CHST9	-1.85	1.70E-05	Carbohydrate sulfotransferase 9
HJV	-1.85	3.44E-04	Hemojuvelin BMP co-receptor
ABCC2	-1.84	2.38E-02	ATP binding cassette subfamily C member 2
CHRNA5	-1.84	1.77E-06	Cholinergic receptor nicotinic alpha 5 subunit
CPNE9	-1.83	4.42E-02	Copine family member 9
SPTBN2	-1.83	1.21E-02	Spectrin beta, non-erythrocytic 2
NEXMIF	-1.83	7.08E-03	Neurite extension and migration factor
THY1	-1.83	1.25E-03	Thy-1 cell surface antigen
FGF1	-1.83	1.51E-06	Fibroblast growth factor 1
NEFL	-1.83	3.88E-02	Neurofilament light chain
MYOCD	-1.83	7.21E-04	Myocardin
EIF4EBP1	-1.83	5.25E-16	Eukaryotic translation initiation factor 4E binding protein 1

COLQ	-1.82	5.83E-04	Collagen like tail subunit of asymmetric acetylcholinesterase
CDKN1C	-1.82	2.15E-04	Cyclin dependent kinase inhibitor 1C
KCND2	-1.81	1.89E-02	Potassium voltage-gated channel subfamily D member 2
SCN4B	-1.81	3.04E-03	Sodium voltage-gated channel beta subunit 4
HIBADH	-1.81	1.18E-05	3-hydroxyisobutyrate dehydrogenase
HELZ2	-1.81	1.31E-03	Helicase with zinc finger 2
ACE	-1.81	4.60E-03	Angiotensin I converting enzyme
IFITM2	-1.81	7.93E-06	Interferon-induced transmembrane protein 2
VSTM2B	-1.81	1.02E-03	V-set and transmembrane domain containing 2B
DMGDH	-1.81	1.84E-07	Dimethylglycine dehydrogenase
SEC14L5	-1.80	6.35E-11	SEC14 like lipid binding 5
PLN	-1.80	2.52E-02	Phospholamban
MYF5	-1.80	1.28E-05	Myogenic factor 5
PMP22	-1.80	2.19E-03	Peripheral myelin protein 22
RASGRF1	-1.80	4.14E-03	Ras protein specific guanine nucleotide releasing factor 1
ANGPTL4	-1.80	1.57E-03	Angiopoietin like 4
CCDC3	-1.79	3.49E-03	Coiled-coil domain containing 3
SMYD1	-1.79	2.29E-04	SET and MYND domain containing 1
WASF1	-1.79	9.18E-03	WASP family member 1
ARHGAP23	-1.78	2.63E-02	Rho GTPase activating protein 23
SRL	-1.78	5.30E-06	Sarcalumenin
SEMA3B	-1.77	4.28E-03	Semaphorin 3B
MVD	-1.77	9.78E-04	Mevalonate diphosphate decarboxylase
EYA2	-1.76	2.37E-03	EYA transcriptional coactivator and phosphatase 2
FAM189A1	-1.76	1.69E-02	Family with sequence similarity 189 member A1
SLC6A6	-1.76	1.41E-06	Solute carrier family 6 member 6
MFAP2	-1.76	7.80E-03	Microfibril associated protein 2
TBX4	-1.76	7.58E-06	T-box transcription factor 4
SSUH2	-1.75	1.84E-02	Ssu-2 homolog
FAM110A	-1.75	3.99E-02	Family with sequence similarity 110 member A
PCDH7	-1.75	2.84E-02	Protocadherin 7
SFRP4	-1.75	1.52E-02	Secreted frizzled related protein 4
KLHL35	-1.75	2.85E-02	Kelch like family member 35
HORMAD2	-1.74	4.21E-02	HORMA domain containing 2
SLC25A47	-1.74	4.44E-05	Solute carrier family 25 member 47
LRP11	-1.74	1.77E-03	LDL receptor related protein 11
FGF5	-1.74	1.26E-02	Fibroblast growth factor 5
AMOTL1	-1.73	7.02E-04	Angiomotin like 1
SLC25A25	-1.73	8.30E-05	Solute carrier family 25 member 25

CTNND2	-1.73	6.12E-06	Catenin delta 2
TEAD3	-1.72	1.08E-02	TEA domain transcription factor 3
SPTB	-1.72	1.10E-02	Spectrin beta, erythrocytic
MAF	-1.72	2.79E-04	MAF bZIP transcription factor
ATP1A1	-1.72	1.21E-06	ATPase Na+/K+ transporting subunit alpha 1
OIT3	-1.72	3.37E-06	Oncoprotein induced transcript 3
SMOX	-1.72	6.73E-04	Spermine oxidase
SRF	-1.72	1.87E-02	Serum response factor
SLC25A34	-1.71	1.96E-03	Solute carrier family 25 member 34
COL1A2	-1.71	2.50E-05	Collagen type I alpha 2 chain
GDF10	-1.71	2.23E-02	Growth differentiation factor 10
SSTR2	-1.71	8.20E-03	Somatostatin receptor 2
TFRC	-1.70	5.24E-04	Transferrin receptor
CDH17	-1.70	2.89E-02	Cadherin 17
SNTA1	-1.69	2.33E-04	Syntrophin alpha 1
LZTS2	-1.69	1.51E-02	Leucine zipper tumor suppressor 2
CFAP206	-1.69	3.48E-02	Cilia and flagella associated protein 206
FBLIM1	-1.69	2.17E-02	Filamin binding LIM protein 1
TACR1	-1.69	4.70E-03	Tachykinin receptor 1
ADAMTS7	-1.68	3.45E-03	ADAM metallopeptidase with thrombospondin type 1 motif 7
MAP2K3	-1.68	3.30E-04	Mitogen-activated protein kinase kinase 3
UPB1	-1.68	1.83E-03	Beta-ureidopropionase 1
NINJ2	-1.68	1.69E-04	Ninjurin 2
COL24A1	-1.68	6.08E-03	Collagen type XXIV alpha 1 chain
FHAD1	-1.68	1.55E-02	Forkhead associated phosphopeptide binding domain 1
ACACB	-1.68	2.41E-02	Acetyl-CoA carboxylase beta
VIL1	-1.68	2.30E-02	Villin 1
PI15	-1.67	2.94E-02	Peptidase inhibitor 15
SRCIN1	-1.67	2.52E-02	SRC kinase signaling inhibitor 1
GRM8	-1.67	4.69E-03	Glutamate metabotropic receptor 8
GPR179	-1.67	2.27E-02	G protein-coupled receptor 179
PLCH2	-1.67	2.31E-02	Phospholipase C eta 2
CS	-1.67	3.53E-02	Citrate synthase
PDSS1	-1.66	2.96E-05	Decaprenyl diphosphate synthase subunit 1
P4HA3	-1.66	1.17E-03	Prolyl 4-hydroxylase subunit alpha 3
SYPL2	-1.66	7.42E-05	Synaptophysin like 2
ALDH6A1	-1.66	1.05E-07	Aldehyde dehydrogenase 6 family member A1
NRP2	-1.66	2.10E-05	Neuropilin 2
RYR2	-1.66	3.30E-02	Ryanodine receptor 2
FMN2	-1.66	3.33E-02	Formin 2
LRRC4C	-1.66	3.22E-02	Leucine rich repeat containing 4C

MCCC2	-1.65	9.46E-06	Methylcrotonyl-CoA carboxylase subunit 2
GSC	-1.65	4.05E-03	Goosecoid homeobox
GRK1	-1.64	2.70E-02	G protein-coupled receptor kinase 1
RGS16	-1.64	6.08E-03	Regulator of G protein signaling 16
ATP1B1	-1.64	1.10E-03	ATPase Na+/K+ transporting subunit beta 1
KCNK10	-1.64	2.18E-02	Potassium two pore domain channel subfamily K member 10
EVA1A	-1.63	1.22E-04	Eva-1 homolog A, regulator of programmed cell death
ADAM19	-1.63	1.52E-05	ADAM metallopeptidase domain 19
BCKDHB	-1.63	8.80E-07	Branched chain keto acid dehydrogenase E1 subunit beta
TRPM8	-1.63	4.27E-02	Transient receptor potential cation channel subfamily M member 8
ABCB5	-1.63	1.79E-03	ATP binding cassette subfamily B member 5
STAT2	-1.62	4.32E-04	Signal transducer and activator of transcription 2
PHYHD1	-1.62	1.17E-02	Phytanoyl-CoA dioxygenase domain containing 1
GRTP1	-1.62	7.83E-05	Growth hormone regulated TBC protein 1
FBXW7	-1.61	9.71E-05	F-box and WD repeat domain containing 7
FIGNL2	-1.61	7.21E-03	Fidgetin like 2
R3HCC1L	-1.61	1.02E-03	R3H domain and coiled-coil containing 1 like
PTGS1	-1.61	8.30E-03	Prostaglandin-endoperoxide synthase 1
CACNA2D2	-1.60	4.32E-03	Calcium voltage-gated channel auxiliary subunit alpha2delta 2
EXD1	-1.60	3.89E-02	Exonuclease 3'-5' domain containing 1
EIF4EBP2	-1.60	1.10E-03	Eukaryotic translation initiation factor 4E binding protein 2
FSTL4	-1.60	4.80E-02	Follistatin like 4
SLC41A3	-1.59	2.23E-04	Solute carrier family 41 member 3
NMUR1	-1.59	5.92E-04	Neuromedin U receptor 1
RIMS2	-1.59	1.27E-02	Regulating synaptic membrane exocytosis 2
TRAF4	-1.59	2.78E-02	TNF receptor associated factor 4
GLIS2	-1.59	1.21E-02	GLIS family zinc finger 2
FBN3	-1.59	5.23E-04	Fibrillin 3
NFKBIA	-1.58	4.71E-03	NFKB inhibitor alpha
BRINP3	-1.58	1.50E-02	BMP/retinoic acid inducible neural specific 3
ZMAT4	-1.57	3.82E-04	Zinc finger matrin-type 4
TIMELESS	-1.57	2.21E-02	Timeless circadian regulator
PTPRF	-1.56	5.81E-04	Protein tyrosine phosphatase receptor type F
DNER	-1.56	4.83E-02	Delta/notch like EGF repeat containing
USP2	-1.56	3.66E-02	Ubiquitin specific peptidase 2
OSBP2	-1.56	8.18E-03	Oxysterol binding protein 2
AKAP8L	-1.56	1.10E-03	A-kinase anchoring protein 8 like

KLHDC3	-1.56	5.15E-03	Kelch domain containing 3
ADAMTS19	-1.56	1.43E-04	ADAM metallopeptidase with thrombospondin type 1 motif 19
KLHDC8A	-1.55	5.33E-04	Kelch domain containing 8A
C3H2orf50	-1.55	3.95E-03	Chromosome 3 C2orf50 homolog
FOXO3	-1.55	2.09E-03	Forkhead box O3
NPM2	-1.55	2.94E-02	Nucleophosmin/nucleoplasmin 2
DHX58	-1.55	1.11E-02	DExH-box helicase 58
CACNA1G	-1.54	4.75E-04	Calcium voltage-gated channel subunit alpha1 G
MSANTD1	-1.54	2.05E-02	Myb/SANT DNA binding domain containing 1
APC2	-1.54	3.41E-02	APC regulator of WNT signaling pathway 2
PER2	-1.54	1.65E-04	Period circadian regulator 2
AGTR2	-1.54	2.28E-05	Angiotensin II receptor type 2
MST1R	-1.54	1.71E-04	Macrophage stimulating 1 receptor
TTPA	-1.53	8.36E-04	Alpha tocopherol transfer protein
SOCS3	-1.53	5.21E-06	Suppressor of cytokine signaling 3
ABHD12B	-1.52	1.27E-03	Abhydrolase domain containing 12B
ACVR1C	-1.52	8.61E-03	Activin A receptor type 1C
KLF11	-1.52	8.35E-04	Kruppel like factor 11
TOGARAM2	-1.51	2.64E-02	TOG array regulator of axonemal microtubules 2
ADHFE1	-1.51	8.35E-04	Alcohol dehydrogenase iron containing 1
IGFBP4	-1.51	1.65E-03	Insulin like growth factor binding protein 4
EPHX2	-1.51	8.83E-04	Epoxide hydrolase 2
ST3GAL1	-1.51	2.09E-03	ST3 beta-galactoside alpha-2,3-sialyltransferase 1
MCCC1	-1.50	9.61E-05	Methylcrotonyl-CoA carboxylase subunit 1
MUSK	-1.50	1.37E-03	Muscle associated receptor tyrosine kinase
KCNK9	-1.50	5.71E-03	Potassium two pore domain channel subfamily K member 9
SLC15A2	-1.49	3.00E-06	Solute carrier family 15 member 2
UTS2R	-1.48	2.13E-03	Urotensin 2 receptor
PTGER3	-1.48	1.70E-02	Prostaglandin E receptor 3
LZTS1	-1.48	1.02E-04	Leucine zipper tumor suppressor 1
LARGE2	-1.47	8.85E-03	LARGE xylosyl- and glucuronyltransferase 2
NMRK2	-1.47	1.63E-02	Nicotinamide riboside kinase 2
CHP1	-1.47	5.28E-05	Calcineurin like EF-hand protein 1
TRABD2B	-1.47	7.98E-03	TraB domain containing 2B
PLCXD2	-1.47	2.00E-05	Phosphatidylinositol specific phospholipase C X domain containing 2
LGALSL	-1.46	6.80E-03	Galectin like
AIMP1	-1.46	8.57E-04	Aminoacyl tRNA synthetase complex interacting multifunctional protein 1
OLFM1	-1.46	1.34E-02	Olfactomedin 1

GRIA3	-1.45	8.37E-04	Glutamate ionotropic receptor AMPA type subunit 3
TMEM82	-1.45	3.53E-03	Transmembrane protein 82
ACOT7	-1.45	2.96E-04	Acyl-CoA thioesterase 7
C8G	-1.44	1.41E-04	Complement C8 gamma chain
OLFML3	-1.44	1.17E-04	Olfactomedin like 3
PROX1	-1.43	5.99E-03	Prospero homeobox 1
MYC	-1.43	1.31E-02	MYC proto-oncogene, bHLH transcription factor
HADHA	-1.43	9.07E-03	Hydroxyacyl-CoA dehydrogenase trifunctional multienzyme complex subunit alpha
SERINC2	-1.43	5.45E-03	Serine incorporator 2
MYL6	-1.43	8.20E-04	Myosin light chain 6
FLVCR1	-1.42	4.23E-03	FLVCR heme transporter 1
NCOR2	-1.42	2.55E-02	Nuclear receptor corepressor 2
ISYNA1	-1.42	1.11E-03	Inositol-3-phosphate synthase 1
GPX2	-1.42	8.95E-04	Glutathione peroxidase 2
ADGRL3	-1.42	3.25E-04	Adhesion G protein-coupled receptor L3
IRS1	-1.42	2.03E-02	Insulin receptor substrate 1
BCL11A	-1.41	8.86E-04	BAF chromatin remodeling complex subunit BCL11A
NOCT	-1.41	3.12E-03	Nocturnin
NSMF	-1.40	1.76E-02	NMDA receptor synaptonuclear signaling and neuronal migration factor
LYSMD4	-1.40	4.91E-03	LysM domain containing 4
AKAP1	-1.40	5.26E-04	A-kinase anchoring protein 1
MOV10	-1.40	1.79E-02	Mov10 RISC complex RNA helicase
IVD	-1.40	1.13E-03	Isovaleryl-CoA dehydrogenase
MUTYH	-1.40	3.07E-03	MutY DNA glycosylase
PCCA	-1.40	1.16E-08	Propionyl-CoA carboxylase subunit alpha
LRRN1	-1.40	6.09E-04	Leucine rich repeat neuronal 1
GRAMD2A	-1.39	3.43E-03	GRAM domain containing 2A
GPR20	-1.39	1.84E-02	G protein-coupled receptor 20
PNPLA2	-1.39	7.86E-03	Patatin like phospholipase domain containing 2
IRF7	-1.39	2.14E-02	Interferon regulatory factor 7
KLF15	-1.39	3.22E-03	Kruppel like factor 15
RGMA	-1.38	4.88E-04	Repulsive guidance molecule BMP co-receptor a
KCNN3	-1.38	8.34E-03	Potassium calcium-activated channel subfamily N member 3
UVRAG	-1.37	1.26E-03	UV radiation resistance associated
SLC6A13	-1.37	4.04E-02	Solute carrier family 6 member 13
NRG1	-1.37	2.06E-02	Neuregulin 1
GAS2L3	-1.37	7.15E-03	Growth arrest specific 2 like 3
RAMP1	-1.37	8.17E-04	Receptor activity modifying protein 1

ESRRG	-1.37	2.21E-03	Estrogen related receptor gamma
CAVIN4	-1.37	3.12E-08	Caveolae associated protein 4
MPP6	-1.36	5.24E-03	Protein associated with LIN7 2, MAGUK p55 family member
MYO16	-1.36	4.27E-03	Myosin XVI
CDHR1	-1.35	6.64E-04	Cadherin related family member 1
ACAD9	-1.35	4.09E-08	Acyl-CoA dehydrogenase family member 9
THOP1	-1.35	1.69E-02	Thimet oligopeptidase 1
GIPC1	-1.34	1.05E-02	GIPC PDZ domain containing family member 1
CSRNP1	-1.34	9.39E-04	Cysteine and serine rich nuclear protein 1
IGF2	-1.34	1.84E-03	Insulin like growth factor 2
SSTR4	-1.34	2.88E-03	Somatostatin receptor 4
AQP4	-1.34	2.12E-02	Aquaporin 4
RPS27L	-1.34	1.54E-03	Ribosomal protein S27 like
EGR2	-1.33	3.05E-02	Early growth response 2
TMEM158	-1.33	2.44E-02	Transmembrane protein 158
PALM2AKAP2	-1.33	2.25E-04	PALM2 and AKAP2 fusion
MCM5	-1.33	2.99E-04	Minichromosome maintenance complex component 5
CCSER1	-1.32	2.50E-03	Coiled-coil serine rich protein 1
PLEC	-1.32	2.83E-02	Plectin
OSGIN1	-1.32	8.73E-04	Oxidative stress induced growth inhibitor 1
RETSAT	-1.32	1.55E-02	Retinol saturase
PLPP2	-1.32	1.30E-02	Phospholipid phosphatase 2
PLAGL2	-1.32	5.56E-05	PLAG1 like zinc finger 2
ATRNL1	-1.32	4.79E-03	Attractin like 1
GOT1	-1.32	2.11E-03	Glutamic-oxaloacetic transaminase 1
ME2	-1.32	2.25E-02	Malic enzyme 2
IL13RA2	-1.32	1.73E-02	Interleukin 13 receptor subunit alpha 2
HACD1	-1.31	1.42E-04	3-hydroxyacyl-CoA dehydratase 1
P3H2	-1.31	8.98E-03	Prolyl 3-hydroxylase 2
MED9	-1.31	8.69E-03	Mediator complex subunit 9
NR4A2	-1.31	1.75E-02	Nuclear receptor subfamily 4 group A member 2
MTMR4	-1.31	5.13E-03	Myotubularin related protein 4
CXXC4	-1.31	1.49E-02	CXXC finger protein 4
DOT1L	-1.31	2.09E-04	DOT1 like histone lysine methyltransferase
IGDCC4	-1.30	1.31E-02	Immunoglobulin superfamily DCC subclass member 4
MYRF	-1.30	2.52E-03	Myelin regulatory factor
RHPN1	-1.30	8.54E-03	Rhophilin Rho GTPase binding protein 1
ADD3	-1.30	4.96E-04	Adducin 3
BMP2	-1.30	2.42E-03	Bone morphogenetic protein 2
FERMT2	-1.30	1.57E-02	FERM domain containing kindlin 2

FAM184B	-1.30	4.90E-03	Family with sequence similarity 184 member B
SLC9A1	-1.30	4.20E-02	Solute carrier family 9 member A1
FST	-1.29	4.75E-03	Follistatin
HVCN1	-1.29	3.90E-03	Hydrogen voltage gated channel 1
PGGHG	-1.29	4.07E-04	Protein-glucosylgalactosylhydroxylysine glucosidase
ETFA	-1.29	2.15E-08	Electron transfer flavoprotein subunit alpha
AEBP1	-1.29	1.92E-02	AE binding protein 1
GFRA1	-1.29	1.59E-02	GDNF family receptor alpha 1
CTBP2	-1.29	2.40E-02	C-terminal binding protein 2
WFS1	-1.29	1.16E-02	Wolframin ER transmembrane glycoprotein
HS6ST2	-1.29	1.58E-02	Heparan sulfate 6-O-sulfotransferase 2
DVL3	-1.29	1.70E-02	Dishevelled segment polarity protein 3
CHGB	-1.29	1.87E-02	Chromogranin B
AMPD1	-1.28	1.30E-02	Adenosine monophosphate deaminase 1
PURB	-1.28	3.59E-02	Purine rich element binding protein B
KCNC1	-1.28	1.34E-02	Potassium voltage-gated channel subfamily C member 1
TMEM266	-1.28	7.53E-04	Transmembrane protein 266
SLC19A1	-1.28	2.32E-03	Solute carrier family 19 member 1
CENPQ	-1.27	1.83E-02	Centromere protein Q
ST6GALNAC4	-1.27	2.38E-03	ST6 N-acetylgalactosaminide alpha-2,6-sialyltransferase 4
FGFR1	-1.27	1.46E-02	Fibroblast growth factor receptor 1
E2F1	-1.27	1.37E-02	E2F transcription factor 1
RPL3	-1.26	8.26E-06	Ribosomal protein L3
COL12A1	-1.26	3.98E-02	Collagen type XII alpha 1 chain
CCDC137	-1.26	2.15E-02	Coiled-coil domain containing 137
ABCD4	-1.26	4.98E-04	ATP binding cassette subfamily D member 4
DNAJC12	-1.26	7.58E-03	DnaJ heat shock protein family (Hsp40) member C12
KALRN	-1.26	8.99E-05	Kalirin RhoGEF kinase
NT5C2	-1.25	5.84E-03	5'-nucleotidase, cytosolic II
PTDSS1	-1.25	8.40E-04	Phosphatidylserine synthase 1
PPP1R12B	-1.25	4.86E-03	Protein phosphatase 1 regulatory subunit 12B
ADAMTS1	-1.25	2.31E-02	ADAM metallopeptidase with thrombospondin type 1 motif 1
CDC20	-1.24	1.62E-02	Cell division cycle 20
SLC16A6	-1.24	7.65E-04	Solute carrier family 16 member 6
ADGRB3	-1.24	2.92E-02	Adhesion G protein-coupled receptor B3
FTH1	-1.24	3.19E-06	Ferritin heavy chain 1
CADM1	-1.24	5.30E-04	Cell adhesion molecule 1
MCF2	-1.23	2.22E-02	MCF.2 cell line derived transforming sequence

DBF4B	-1.23	5.03E-04	DBF4 zinc finger B
FGF12	-1.23	7.29E-04	Fibroblast growth factor 12
GTF2IRD1	-1.23	9.22E-03	GTF2I repeat domain containing 1
PPM1D	-1.23	9.89E-05	Protein phosphatase, Mg2+/Mn2+ dependent 1D
ABCA2	-1.22	1.57E-02	ATP binding cassette subfamily A member 2
CHAMP1	-1.22	5.88E-03	Chromosome alignment maintaining phosphoprotein 1
ADCY3	-1.22	3.18E-02	Adenylate cyclase 3
PTMA	-1.22	1.67E-02	Prothymosin alpha
SARDH	-1.22	3.64E-02	Sarcosine dehydrogenase
EPHB2	-1.22	1.49E-05	EPH receptor B2
MOS	-1.21	2.94E-02	MOS proto-oncogene, serine/threonine kinase
ARX	-1.21	4.41E-02	Aristaless related homeobox
SORT1	-1.21	4.73E-02	Sortilin 1
OXTR	-1.21	4.26E-02	Oxytocin receptor
DDHD1	-1.21	3.30E-04	DDHD domain containing 1
LIG1	-1.20	2.85E-02	DNA ligase 1
SIK1	-1.20	7.54E-04	Salt inducible kinase 1
RNF207	-1.19	1.63E-02	Ring finger protein 207
TEF	-1.19	1.78E-04	TEF transcription factor, PAR bZIP family member
SFXN2	-1.19	1.20E-02	Sideroflexin 2
FOXP4	-1.19	1.68E-02	Forkhead box P4
POLR3GL	-1.19	1.55E-02	RNA polymerase III subunit GL
LPCAT3	-1.19	4.51E-04	Lysophosphatidylcholine acyltransferase 3
ACADL	-1.19	4.48E-08	Acyl-CoA dehydrogenase long chain
NPNT	-1.18	2.29E-02	Nephronectin
SNX19	-1.18	9.66E-05	Sorting nexin 19
GABBR1	-1.18	1.13E-03	Gamma-aminobutyric acid type B receptor subunit 1
P2RY2	-1.18	2.25E-03	Purinergic receptor P2Y2
COL13A1	-1.18	3.35E-03	Collagen type XIII alpha 1 chain
POGLUT1	-1.18	3.95E-04	Protein O-glucosyltransferase 1
TSSC4	-1.18	1.01E-03	Tumor suppressing subtransferable candidate 4
VAV2	-1.18	1.14E-02	Vav guanine nucleotide exchange factor 2
SLC25A33	-1.17	5.66E-03	Solute carrier family 25 member 33
KLF4	-1.17	4.92E-03	Kruppel like factor 4
PTPN23	-1.17	4.69E-02	Protein tyrosine phosphatase non-receptor type 23
MBD3	-1.17	1.38E-05	Methyl-CpG binding domain protein 3
PANX2	-1.17	4.70E-02	Pannexin 2
EYA1	-1.17	7.78E-03	EYA transcriptional coactivator and phosphatase 1
TJP3	-1.17	4.53E-02	Tight junction protein 3

ADAM33	-1.17	4.31E-02	ADAM metallopeptidase domain 33
HLF	-1.17	5.46E-04	HLF transcription factor, PAR bZIP family member
FAM20A	-1.16	5.18E-03	FAM20A golgi associated secretory pathway pseudokinase
PYCR3	-1.16	1.07E-02	Pyrroline-5-carboxylate reductase 3
ST6GALNAC6	-1.16	8.11E-03	ST6 N-acetylgalactosaminide alpha-2,6-sialyltransferase 6
XRCC6	-1.16	3.93E-03	X-ray repair cross complementing 6
ABAT	-1.16	1.58E-03	4-aminobutyrate aminotransferase
TMEM11	-1.16	7.95E-03	Transmembrane protein 11
GALNTL6	-1.16	2.93E-02	Polypeptide N-acetylgalactosaminyltransferase like 6
NYX	-1.16	4.82E-02	Nyctalopin
KLF5	-1.16	2.45E-03	Kruppel like factor 5
STK32C	-1.15	2.88E-02	Serine/threonine kinase 32C
MTHFD2	-1.15	7.13E-03	Methylenetetrahydrofolate dehydrogenase (NADP+ dependent) 2, methenyltetrahydrofolate cyclohydrolase
SPATA18	-1.15	5.85E-03	Spermatogenesis associated 18
TOR3A	-1.15	5.60E-03	Torsin family 3 member A
PARM1	-1.14	1.76E-02	Prostate androgen-regulated mucin-like protein 1
ABCB8	-1.14	2.72E-02	ATP binding cassette subfamily B member 8
DNAJC5G	-1.14	1.55E-02	DnaJ heat shock protein family (Hsp40) member C5 gamma
PLCD3	-1.14	1.43E-02	Phospholipase C delta 3
SLC25A4	-1.14	3.60E-04	Solute carrier family 25 member 4
PLPP3	-1.13	2.88E-04	Phospholipid phosphatase 3
PLXNA1	-1.13	2.53E-02	Plexin A1
PSMA5	-1.13	2.62E-02	Proteasome 20S subunit alpha 5
MEOX1	-1.13	1.76E-02	Mesenchyme homeobox 1
RHBDL3	-1.13	4.34E-02	Rhomboid like 3
DDIT3	-1.13	1.89E-02	DNA damage inducible transcript 3
ARAP1	-1.13	2.59E-02	ArfGAP with RhoGAP domain, ankyrin repeat and PH domain 1
TENT5B	-1.13	1.42E-02	Terminal nucleotidyltransferase 5B
SSPN	-1.12	1.03E-03	Sarcospan
NDUFAF8	-1.12	5.54E-03	NADH:ubiquinone oxidoreductase complex assembly factor 8
BCL2	-1.12	3.38E-05	BCL2 apoptosis regulator
FTSJ3	-1.12	3.68E-02	FtsJ RNA 2'-O-methyltransferase 3
TNIK	-1.12	2.31E-02	TRAF2 and NCK interacting kinase
RBFOX2	-1.11	2.06E-02	RNA binding fox-1 homolog 2

RPL15	-1.11	2.90E-02	Ribosomal protein L15
NDUFS2	-1.11	7.77E-03	NADH:ubiquinone oxidoreductase core subunit S2
WDR18	-1.11	3.04E-02	WD repeat domain 18
DUSP1	-1.11	3.88E-02	Dual specificity phosphatase 1
MN1	-1.11	1.31E-02	MN1 proto-oncogene, transcriptional regulator
MATN2	-1.11	7.88E-04	Matrilin 2
SEMA7A	-1.10	3.28E-02	Semaphorin 7A (John Milton Hagen blood group)
SETBP1	-1.10	5.24E-05	SET binding protein 1
WHAMM	-1.10	1.26E-03	WASP homolog associated with actin, golgi membranes and microtubules
NACA	-1.10	2.81E-03	Nascent polypeptide associated complex subunit alpha
BCAR3	-1.10	2.52E-02	BCAR3 adaptor protein, NSP family member
ID2	-1.10	3.63E-05	Inhibitor of DNA binding 2
ZNF536	-1.10	4.85E-02	Zinc finger protein 536
ZBTB37	-1.10	5.36E-05	Zinc finger and BTB domain containing 37
LRRC42	-1.09	4.10E-04	Leucine rich repeat containing 42
FRMD3	-1.09	2.10E-02	FERM domain containing 3
DENND2B	-1.09	3.27E-03	DENN domain containing 2B
BRD3OS	-1.09	2.92E-04	BRD3 opposite strand
AATF	-1.09	1.57E-02	Apoptosis antagonizing transcription factor
ELMO3	-1.09	4.25E-02	Engulfment and cell motility 3
FITM2	-1.08	3.01E-03	Fat storage inducing transmembrane protein 2
PRR16	-1.08	8.44E-03	Proline rich 16
ADAMTS15	-1.08	6.52E-04	ADAM metallopeptidase with thrombospondin type 1 motif 15
GPR146	-1.08	5.65E-03	G protein-coupled receptor 146
RANBP10	-1.08	3.40E-03	RAN binding protein 10
ABLIM2	-1.08	2.04E-06	Actin binding LIM protein family member 2
UBAP2	-1.08	1.73E-02	Ubiquitin associated protein 2
LAMB1	-1.07	9.44E-03	Laminin subunit beta 1
JARID2	-1.07	7.18E-03	Jumonji and AT-rich interaction domain containing 2
ANKRD52	-1.07	1.07E-02	Ankyrin repeat domain 52
MAT1A	-1.07	2.68E-02	Methionine adenosyltransferase 1A
YAP1	-1.07	1.53E-04	Yes1 associated transcriptional regulator
ASH2L	-1.07	6.38E-06	ASH2 like, histone lysine methyltransferase complex subunit
TIMM10	-1.07	2.30E-04	Translocase of inner mitochondrial membrane 10
FOXRED1	-1.06	4.25E-02	FAD dependent oxidoreductase domain containing 1
CHRNG	-1.06	4.75E-02	Cholinergic receptor nicotinic gamma subunit

HADHB	-1.06	4.03E-04	Hydroxyacyl-CoA dehydrogenase trifunctional multienzyme complex subunit beta
ADAMTS20	-1.06	8.32E-03	ADAM metallopeptidase with thrombospondin type 1 motif 20
THUMPD2	-1.06	3.63E-03	THUMP domain containing 2
MAFF	-1.06	4.77E-02	MAF bZIP transcription factor F
HYKK	-1.06	1.81E-04	Hydroxylysine kinase
ZNF804A	-1.06	3.69E-02	Zinc finger protein 804A
DUS2	-1.06	3.74E-04	Dihydrouridine synthase 2
HHATL	-1.06	1.47E-03	Hedgehog acyltransferase like
IGFBP3	-1.06	1.81E-03	Insulin like growth factor binding protein 3
RAD54L2	-1.05	1.56E-02	RAD54 like 2
MYO7A	-1.05	8.38E-03	Myosin VIIA
BZW2	-1.05	2.44E-03	Basic leucine zipper and W2 domains 2
TBC1D8	-1.05	2.25E-04	TBC1 domain family member 8
ANKRD60	-1.05	2.70E-03	Ankyrin repeat domain 60
SMAD7	-1.05	4.86E-02	SMAD family member 7
GRPEL2	-1.04	2.01E-04	GrpE like 2, mitochondrial
GDPD5	-1.04	2.13E-04	Glycerophosphodiester phosphodiesterase domain containing 5
POLH	-1.04	7.98E-04	DNA polymerase eta
ASPHD2	-1.04	4.68E-02	Aspartate beta-hydroxylase domain containing 2
PIN1	-1.04	1.51E-02	Peptidylprolyl cis/trans isomerase, NIMA-interacting 1
DGKZ	-1.03	1.59E-02	Diacylglycerol kinase zeta
JPH2	-1.02	2.25E-02	Junctophilin 2
CITED2	-1.02	1.63E-03	Cbp/p300 interacting transactivator with Glu/Asp rich carboxy-terminal domain 2
PSIP1	-1.02	1.38E-02	PC4 and SFRS1 interacting protein 1
FZD7	-1.02	2.37E-02	Frizzled class receptor 7
RHOBTB2	-1.02	2.31E-02	Rho related BTB domain containing 2
PARP6	-1.02	3.10E-02	Poly(ADP-ribose) polymerase family member 6
SPR	-1.02	3.53E-02	Sepiapterin reductase
MMS19	-1.02	4.99E-02	MMS19 homolog, cytosolic iron-sulfur assembly component
ACAA2	-1.02	2.21E-04	Acetyl-CoA acyltransferase 2
TRPC6	-1.02	4.10E-02	Transient receptor potential cation channel subfamily C member 6
ZNF512B	-1.02	3.72E-02	Zinc finger protein 512B
SLC25A10	-1.02	2.55E-02	Solute carrier family 25 member 10
HDAC4	-1.02	1.43E-02	Histone deacetylase 4
AXIN2	-1.02	2.43E-02	Axin 2
PLCE1	-1.02	1.03E-04	Phospholipase C epsilon 1

PDGFRL	-1.01	1.59E-02	Platelet derived growth factor receptor like
LSM7	-1.01	5.69E-04	LSM7 homolog, U6 small nuclear RNA and mRNA degradation associated
CASQ2	-1.01	1.98E-02	Calsequestrin 2
NDUFV1	-1.01	3.43E-02	NADH:ubiquinone oxidoreductase core subunit V1
NOP58	-1.01	3.30E-04	NOP58 ribonucleoprotein
PKD1	-1.01	2.38E-02	Polycystin 1, transient receptor potential channel interacting
HIGD1A	-1.00	4.19E-02	HIG1 hypoxia inducible domain family member 1A
MICU1	-1.00	2.67E-02	Mitochondrial calcium uptake 1
SGCA	-1.00	9.82E-03	Sarcoglycan alpha
KCNAB1	-1.00	1.47E-02	Potassium voltage-gated channel subfamily A regulatory beta subunit 1
MYH11	-1.00	3.23E-02	Myosin heavy chain 11
DPF3	-1.00	1.42E-02	Double PHD fingers 3
TRIM55	-1.00	4.32E-02	Tripartite motif containing 55
SDC4	-1.00	1.12E-02	Syndecan 4
OSER1	1.00	5.43E-03	Oxidative stress responsive serine rich 1
CCT8	1.00	1.75E-04	Chaperonin containing TCP1 subunit 8
HS3ST5	1.00	9.91E-03	Heparan sulfate-glucosamine 3-sulfotransferase 5
GALNT10	1.00	1.06E-03	Polypeptide N-acetylgalactosaminyltransferase 10
ERAP1	1.00	6.52E-03	Endoplasmic reticulum aminopeptidase 1
RHAG	1.00	4.17E-02	Rh associated glycoprotein
POFUT2	1.01	1.42E-03	Protein O-fucosyltransferase 2
OFD1	1.01	7.99E-03	OFD1 centriole and centriolar satellite protein
NRK	1.01	9.71E-04	Nik related kinase
GLMN	1.01	3.46E-02	Glomulin, FKBP associated protein
TARBP1	1.01	9.39E-03	TAR (HIV-1) RNA binding protein 1
GOLT1B	1.01	4.99E-02	Golgi transport 1B
C1H12orf4	1.01	3.33E-03	Chromosome 1 C12orf4 homolog
LCMT1	1.01	6.49E-03	Leucine carboxyl methyltransferase 1
ALDH2	1.01	3.96E-03	Aldehyde dehydrogenase 2 family member
IQGAP1	1.01	6.82E-03	IQ motif containing GTPase activating protein 1
PYGO1	1.01	1.20E-02	Pygopus family PHD finger 1
B3GNTL1	1.01	1.81E-02	UDP-GlcNAc:betaGal beta-1,3-N-acetylglucosaminyltransferase like 1
SERPINC1	1.01	1.25E-02	Serpin family C member 1
SAMD8	1.02	2.92E-02	Sterile alpha motif domain containing 8
NARF	1.02	1.62E-03	Nuclear prelamin A recognition factor
AP4S1	1.02	2.54E-02	Adaptor related protein complex 4 subunit sigma 1

TMEM200A	1.03	1.26E-02	Transmembrane protein 200A
TBCE	1.03	1.60E-03	Tubulin folding cofactor E
TMEM64	1.03	2.58E-02	Transmembrane protein 64
SLC9A8	1.03	2.47E-02	Solute carrier family 9 member A8
FAM181B	1.03	2.81E-02	Family with sequence similarity 181 member B
SPON2	1.03	7.12E-04	Spondin 2
ISCA1	1.03	7.89E-03	Iron-sulfur cluster assembly 1
LTBP1	1.03	3.80E-02	Latent transforming growth factor beta binding protein 1
C22H1orf159	1.03	2.10E-02	Chromosome 22 C1orf159 homolog
PNPLA4	1.03	8.02E-03	Patatin like phospholipase domain containing 4
ADGRG6	1.04	3.38E-02	Adhesion G protein-coupled receptor G6
TRADD	1.04	5.55E-03	TNFRSF1A associated via death domain
ITGA1	1.04	2.41E-04	Integrin subunit alpha 1
TPK1	1.04	4.37E-02	Thiamin pyrophosphokinase 1
BLVRB	1.04	2.72E-03	Biliverdin reductase B
RP9	1.04	8.36E-03	RP9 pre-mRNA splicing factor
TSPAN13	1.04	7.25E-05	Tetraspanin 13
CNDP2	1.04	1.58E-02	Carnosine dipeptidase 2
CAPS2	1.04	2.10E-02	Calcyphosine 2
PPP1R3A	1.04	4.01E-04	Protein phosphatase 1 regulatory subunit 3A
LBHD2	1.04	3.85E-03	LBH domain containing 2
CUL5	1.04	4.82E-03	Cullin 5
TMTC2	1.04	3.65E-03	Transmembrane O-mannosyltransferase targeting cadherins 2
MSRB1	1.05	1.03E-02	Methionine sulfoxide reductase B1
SCARB1	1.05	2.81E-03	Scavenger receptor class B member 1
PIK3CB	1.05	3.92E-02	Phosphatidylinositol-4,5-bisphosphate 3-kinase catalytic subunit beta
TSPAN9	1.05	1.49E-03	Tetraspanin 9
FAM180B	1.05	2.61E-02	Family with sequence similarity 180 member B
CPED1	1.05	1.30E-05	Cadherin like and PC-esterase domain containing 1
TANK	1.05	2.95E-02	TRAF family member associated NFKB activator
TYRO3	1.05	3.17E-03	TYRO3 protein tyrosine kinase
PLEKHB2	1.06	1.92E-03	Pleckstrin homology domain containing B2
CZH9orf85	1.06	1.82E-02	Chromosome Z C9orf85 homolog
TM4SF18	1.06	2.10E-02	Transmembrane 4 L six family member 18
DST	1.06	6.00E-05	Dystonin
HEXA	1.06	6.78E-04	Hexosaminidase subunit alpha
HDAC10	1.06	6.85E-03	Histone deacetylase 10
CLIC4	1.06	4.17E-03	Chloride intracellular channel 4
SLC25A43	1.06	3.27E-02	Solute carrier family 25 member 43

MDFIC	1.06	2.60E-02	MyoD family inhibitor domain containing
DLEC1	1.06	4.62E-02	DLEC1 cilia and flagella associated protein
ARHGEF28	1.07	1.58E-02	Rho guanine nucleotide exchange factor 28
CNTRL	1.07	4.36E-03	Centriolin
TNFSF10	1.07	1.85E-03	TNF superfamily member 10
WDR35	1.07	3.40E-02	WD repeat domain 35
MDM1	1.07	3.07E-02	Mdm1 nuclear protein
TMEM42	1.07	4.92E-03	Transmembrane protein 42
RGS14	1.07	2.49E-02	Regulator of G protein signaling 14
LSP1	1.07	3.50E-03	Lymphocyte specific protein 1
TUBB4B	1.07	1.45E-03	Tubulin beta 4B class IVb
TRNT1	1.07	1.06E-02	TRNA nucleotidyl transferase 1
FN1	1.08	1.60E-02	Fibronectin 1
PODXL2	1.08	1.05E-02	Podocalyxin like 2
KITLG	1.08	1.27E-03	KIT ligand
ATPAF1	1.08	4.15E-03	ATP synthase mitochondrial F1 complex assembly factor 1
CNIH4	1.08	4.93E-03	Cornichon family AMPA receptor auxiliary protein 4
PLBD2	1.08	2.58E-02	Phospholipase B domain containing 2
USP25	1.08	1.64E-05	Ubiquitin specific peptidase 25
HOOK3	1.08	7.67E-05	Hook microtubule tethering protein 3
ARHGDIG	1.08	4.79E-02	Rho GDP dissociation inhibitor gamma
FBXL2	1.08	7.39E-03	F-box and leucine rich repeat protein 2
PIP4P2	1.08	3.96E-03	Phosphatidylinositol-4,5-bisphosphate 4-phosphatase 2
LRMDA	1.08	4.04E-02	Leucine rich melanocyte differentiation associated
SNX5	1.09	1.26E-02	Sorting nexin 5
PGD	1.09	1.02E-02	Phosphogluconate dehydrogenase
MCFD2	1.09	1.14E-02	Multiple coagulation factor deficiency 2, ER cargo receptor complex subunit
GNPDA1	1.09	2.75E-03	Glucosamine-6-phosphate deaminase 1
GSDME	1.09	7.54E-03	Gasdermin E
BRCA1	1.09	9.53E-03	BRCA1 DNA repair associated
ARMC10	1.09	2.13E-02	Armadillo repeat containing 10
IL17RE	1.09	3.19E-02	Interleukin 17 receptor E
RAB5A	1.09	1.53E-02	RAB5A, member RAS oncogene family
DPY19L3	1.09	3.94E-02	Dpy-19 like C-mannosyltransferase 3
DGKH	1.09	3.92E-03	Diacylglycerol kinase eta
FMNL1	1.10	3.20E-03	Formin like 1
NRROS	1.10	3.48E-02	Negative regulator of reactive oxygen species
FKBP9	1.10	4.51E-06	FKBP prolyl isomerase 9
CPNE2	1.10	1.48E-02	Copine 2

PPP1R3G	1.10	8.80E-04	Protein phosphatase 1 regulatory subunit 3G
NADK2	1.10	1.34E-02	NAD kinase 2, mitochondrial
CD59	1.11	2.13E-03	CD59 molecule (CD59 blood group)
IDUA	1.11	3.66E-03	Alpha-L-iduronidase
SAMHD1	1.11	3.73E-02	SAM and HD domain containing deoxynucleoside triphosphate triphosphohydrolase 1
MMP2	1.11	6.12E-03	Matrix metallopeptidase 2
DBF4	1.11	4.90E-02	DBF4 zinc finger
STAB1	1.11	2.28E-03	Stabilin 1
BIN2	1.11	1.84E-02	Bridging integrator 2
PM20D1	1.12	2.75E-04	Peptidase M20 domain containing 1
EDN1	1.12	2.67E-02	Endothelin 1
GANC	1.12	8.57E-05	Glucosidase alpha, neutral C
HTD2	1.12	4.55E-02	Hydroxyacyl-thioester dehydratase type 2
EML1	1.12	3.28E-03	EMAP like 1
GALM	1.12	5.76E-03	Galactose mutarotase
PDZRN3	1.12	7.24E-03	PDZ domain containing ring finger 3
PDLIM3	1.13	2.79E-02	PDZ and LIM domain 3
ORC3	1.13	2.13E-03	Origin recognition complex subunit 3
PLXNB2	1.13	2.63E-03	Plexin B2
IPCEF1	1.13	1.47E-02	Interaction protein for cytohesin exchange factors 1
UBE3C	1.13	1.54E-03	Ubiquitin protein ligase E3C
PIBF1	1.13	4.01E-02	Progesterone immunomodulatory binding factor 1
UROS	1.13	9.15E-03	Uroporphyrinogen III synthase
DNAJC10	1.13	1.96E-02	DnaJ heat shock protein family (Hsp40) member C10
PTTG1	1.14	2.37E-02	PTTG1 regulator of sister chromatid separation, securin
ARL6IP1	1.14	5.05E-03	ADP ribosylation factor like GTPase 6 interacting protein 1
STK24	1.14	4.95E-02	Serine/threonine kinase 24
KPNA3	1.14	6.93E-07	Karyopherin subunit alpha 3
OSTM1	1.14	9.63E-03	Osteoclastogenesis associated transmembrane protein 1
RNF2	1.14	3.77E-03	Ring finger protein 2
CAPNS2	1.14	3.19E-02	Calpain small subunit 2
EDEM1	1.14	1.32E-02	ER degradation enhancing alpha-mannosidase like protein 1
MMEL1	1.14	2.67E-03	Membrane metalloendopeptidase like 1
CNTNAP5	1.14	2.27E-03	Contactin associated protein family member 5
ACSS3	1.15	4.68E-03	Acyl-CoA synthetase short chain family member 3

SHLD3	1.15	3.30E-02	Shieldin complex subunit 3
DOCK4	1.15	6.00E-04	Dedicator of cytokinesis 4
CARNMT1	1.15	1.36E-02	Carnosine N-methyltransferase 1
VPS13C	1.15	3.86E-03	Vacuolar protein sorting 13 homolog C
BCAP29	1.15	6.37E-03	B cell receptor associated protein 29
CCDC9B	1.15	2.93E-02	Coiled-coil domain containing 9B
FAM189A2	1.15	8.65E-03	Family with sequence similarity 189 member A2
TMSB4X	1.16	3.32E-03	Thymosin beta 4 X-linked
LRRC30	1.16	9.71E-03	Leucine rich repeat containing 30
TCTN2	1.16	3.20E-02	Tectonic family member 2
HABP4	1.16	2.72E-03	Hyaluronan binding protein 4
IFT80	1.16	7.41E-03	Intraflagellar transport 80
TACC2	1.16	1.37E-03	Transforming acidic coiled-coil containing protein 2
MAMDC2	1.17	9.06E-03	MAM domain containing 2
ANKRD6	1.17	1.39E-02	Ankyrin repeat domain 6
CCDC107	1.17	1.38E-02	Coiled-coil domain containing 107
SPNS3	1.17	4.51E-02	Sphingolipid transporter 3 (putative)
WDR76	1.17	7.88E-03	WD repeat domain 76
ARPC5L	1.18	6.76E-03	Actin related protein 2/3 complex subunit 5 like
SRD5A3	1.18	1.11E-02	Steroid 5 alpha-reductase 3
KIFAP3	1.18	1.35E-03	Kinesin associated protein 3
WIPF1	1.18	6.31E-04	WAS/WASL interacting protein family member 1
PTAR1	1.18	5.48E-03	Protein prenyltransferase alpha subunit repeat containing 1
FAM3C	1.18	2.58E-02	FAM3 metabolism regulating signaling molecule C
TTC19	1.18	1.01E-02	Tetratricopeptide repeat domain 19
SPDL1	1.18	4.37E-03	Spindle apparatus coiled-coil protein 1
AQP3	1.19	1.43E-02	Aquaporin 3 (Gill blood group)
MARCKS	1.19	4.49E-03	Myristoylated alanine rich protein kinase C substrate
COPS2	1.19	1.13E-02	COP9 signalosome subunit 2
SMARCA1	1.19	1.10E-02	SWI/SNF related, matrix associated, actin dependent regulator of chromatin, subfamily a, member 1
FAM174B	1.19	7.46E-05	Family with sequence similarity 174 member B
INPP5D	1.19	4.19E-03	Inositol polyphosphate-5-phosphatase D
KRIT1	1.19	2.93E-03	KRIT1 ankyrin repeat containing
ITGAV	1.19	8.37E-03	Integrin subunit alpha V
SGPP1	1.19	1.39E-02	Sphingosine-1-phosphate phosphatase 1
RNFT2	1.19	1.13E-05	Ring finger protein, transmembrane 2
FASTKD1	1.19	1.25E-02	FAST kinase domains 1

CPXM2	1.19	3.31E-02	Carboxypeptidase X, M14 family member 2
LZIC	1.20	9.38E-04	Leucine zipper and CTNNBIP1 domain containing
TMSB15B	1.20	4.27E-05	Thymosin beta 15B
SLC43A2	1.20	6.28E-03	Solute carrier family 43 member 2
RRAGD	1.20	4.82E-03	Ras related GTP binding D
RBBP8	1.20	2.73E-03	RB binding protein 8, endonuclease
SMC4	1.21	8.73E-03	Structural maintenance of chromosomes 4
CETN3	1.21	2.69E-02	Centrin 3
MGST1	1.21	4.33E-02	Microsomal glutathione S-transferase 1
BLOC1S2	1.21	4.01E-02	Biogenesis of lysosomal organelles complex 1 subunit 2
PLCD4	1.21	1.30E-02	Phospholipase C delta 4
AFF2	1.21	9.64E-04	AF4/FMR2 family member 2
TAMM41	1.21	7.37E-03	TAM41 mitochondrial translocator assembly and maintenance homolog
GNE	1.21	1.15E-02	Glucosamine (UDP-N-acetyl)-2-epimerase/N-acetylmannosamine kinase
MPND	1.22	2.98E-02	MPN domain containing
ATP1B4	1.22	2.37E-02	ATPase Na+/K+ transporting family member beta 4
KAT2B	1.22	8.31E-04	Lysine acetyltransferase 2B
MEGF6	1.22	4.71E-02	Multiple EGF like domains 6
GTPBP10	1.22	2.62E-04	GTP binding protein 10
TMEM37	1.22	1.25E-02	Transmembrane protein 37
PLAT	1.22	2.57E-04	Plasminogen activator, tissue type
KCNF1	1.22	1.37E-02	Potassium voltage-gated channel modifier subfamily F member 1
MTPN	1.22	1.37E-03	Myotrophin
RIPK1	1.22	1.76E-02	Receptor interacting serine/threonine kinase 1
PEX2	1.23	1.82E-02	Peroxisomal biogenesis factor 2
IFNAR1	1.23	2.51E-02	Interferon alpha and beta receptor subunit 1
PIK3AP1	1.23	3.58E-02	Phosphoinositide-3-kinase adaptor protein 1
MYPN	1.23	3.79E-05	Myopalladin
WASHC4	1.23	1.75E-03	WASH complex subunit 4
KIAA1210	1.23	2.00E-03	KIAA1210 ortholog
NCOA7	1.24	5.61E-03	Nuclear receptor coactivator 7
SPART	1.24	2.83E-03	Spartin
ECRG4	1.24	1.55E-03	ECRG4 augurin precursor
MNS1	1.24	2.07E-02	Meiosis specific nuclear structural 1
ARMC2	1.24	1.82E-03	Armadillo repeat containing 2
LZTFL1	1.24	1.14E-02	Leucine zipper transcription factor like 1
MAPK9	1.24	1.04E-03	Mitogen-activated protein kinase 9

NDUFAF1	1.24	1.90E-05	NADH:ubiquinone oxidoreductase complex assembly factor 1
HAUS8	1.24	5.26E-03	HAUS augmin like complex subunit 8
SLC66A3	1.24	2.30E-02	Solute carrier family 66 member 3
SMIM14	1.24	1.76E-03	Small integral membrane protein 14
SLC38A1	1.24	2.21E-02	Solute carrier family 38 member 1
LRRC49	1.24	8.86E-03	Leucine rich repeat containing 49
SRP14	1.24	1.39E-03	Signal recognition particle 14
TMEM144	1.24	2.96E-02	Transmembrane protein 144
CCNC	1.25	4.53E-02	Cyclin C
CENPX	1.25	1.48E-03	Centromere protein X
RUNX1	1.25	6.21E-03	RUNX family transcription factor 1
ANGPTL1	1.25	1.77E-04	Angiopoietin like 1
SULF2	1.25	1.73E-02	Sulfatase 2
RNF150	1.25	9.09E-05	Ring finger protein 150
FAM53A	1.26	7.47E-05	Family with sequence similarity 53 member A
JAK2	1.26	5.93E-03	Janus kinase 2
ANKRD44	1.26	2.66E-03	Ankyrin repeat domain 44
ATP8A1	1.26	1.81E-04	ATPase phospholipid transporting 8A1
CALHM2	1.26	3.28E-02	Calcium homeostasis modulator family member 2
LYRM2	1.26	2.52E-04	LYR motif containing 2
TUBGCP4	1.26	2.22E-05	Tubulin gamma complex associated protein 4
CLIP1	1.27	2.91E-03	CAP-Gly domain containing linker protein 1
NSMAF	1.27	6.14E-03	Neutral sphingomyelinase activation associated factor
PRXL2C	1.27	1.04E-03	Peroxiredoxin like 2C
RFFL	1.27	1.47E-03	Ring finger and FYVE like domain containing E3 ubiquitin protein ligase
TMEM120B	1.27	1.09E-04	Transmembrane protein 120B
EIF2B1	1.27	1.86E-06	Eukaryotic translation initiation factor 2B subunit alpha
EHD4	1.27	7.20E-07	EH domain containing 4
CASP7	1.27	1.16E-02	Caspase 7
TIMP2	1.27	2.24E-02	TIMP metallopeptidase inhibitor 2
ALKBH4	1.28	1.02E-03	AlkB homolog 4, lysine demethylase
LINS1	1.28	1.46E-02	Lines homolog 1
PTGS2	1.28	3.34E-02	Prostaglandin-endoperoxide synthase 2
SLC37A1	1.28	1.04E-02	Solute carrier family 37 member 1
RNF141	1.28	1.73E-02	Ring finger protein 141
C1QTNF3	1.28	1.08E-03	C1q and TNF related 3
GAS6	1.28	9.47E-04	Growth arrest specific 6
NINJ1	1.28	3.09E-02	Ninjurin 1
LIMA1	1.28	1.30E-03	LIM domain and actin binding 1

CMC4	1.28	3.15E-04	C-X9-C motif containing 4
HSP90B1	1.29	2.28E-05	Heat shock protein 90 beta family member 1
CCNB2	1.29	7.52E-03	Cyclin B2
CD274	1.29	2.23E-02	CD274 molecule
ITGB6	1.29	1.68E-02	Integrin subunit beta 6
ARHGAP29	1.29	8.61E-08	Rho GTPase activating protein 29
DHRS12	1.29	1.77E-03	Dehydrogenase/reductase 12
ANO5	1.29	3.57E-04	Anoctamin 5
BICD1	1.29	3.81E-02	BICD cargo adaptor 1
LCA5L	1.29	6.20E-03	Lebercilin LCA5 like
P2RX7	1.29	2.85E-02	Purinergic receptor P2X 7
ENPP4	1.30	3.36E-04	Ectonucleotide pyrophosphatase/phosphodiesterase 4
TBC1D16	1.30	1.11E-03	TBC1 domain family member 16
EPDR1	1.30	7.93E-04	Ependymin related 1
PPM1E	1.30	3.27E-04	Protein phosphatase, Mg2+/Mn2+ dependent 1E
SIGLEC1	1.30	1.26E-02	Sialic acid binding Ig like lectin 1
NEDD4	1.30	1.29E-05	NEDD4 E3 ubiquitin protein ligase
ST8SIA4	1.30	2.04E-04	ST8 alpha-N-acetyl-neuraminide alpha-2,8-sialyltransferase 4
SLC35D2	1.30	2.39E-02	Solute carrier family 35 member D2
ELOVL5	1.31	4.79E-02	ELOVL fatty acid elongase 5
IMPACT	1.31	2.40E-02	Impact RWD domain protein
ASPM	1.31	2.36E-02	Assembly factor for spindle microtubules
ACTR6	1.31	2.62E-02	Actin related protein 6
PTGR1	1.31	6.06E-05	Prostaglandin reductase 1
VAV1	1.31	5.04E-03	Vav guanine nucleotide exchange factor 1
XYLB	1.32	1.10E-03	Xylulokinase
IFT43	1.32	3.97E-05	Intraflagellar transport 43
TRAF1	1.32	1.47E-02	TNF receptor associated factor 1
DENND1B	1.32	1.32E-03	DENN domain containing 1B
BST1	1.32	4.07E-03	Bone marrow stromal cell antigen 1
ABCC4	1.33	3.49E-06	ATP binding cassette subfamily C member 4
ALDH1A2	1.33	3.24E-04	Aldehyde dehydrogenase 1 family member A2
WLS	1.33	1.62E-05	Wnt ligand secretion mediator
ST6GAL1	1.33	6.69E-03	ST6 beta-galactoside alpha-2,6-sialyltransferase 1
CHST15	1.33	1.49E-02	Carbohydrate sulfotransferase 15
MARCHF1	1.33	1.28E-02	Membrane associated ring-CH-type finger 1
CLEC3B	1.33	2.74E-03	C-type lectin domain family 3 member B
KRTCAP3	1.33	3.84E-02	Keratinocyte associated protein 3
SUGT1	1.34	2.22E-04	SGT1 homolog, MIS12 kinetochore complex assembly cochaperone
MRPL42	1.34	6.70E-06	Mitochondrial ribosomal protein L42

GKAP1	1.34	1.54E-03	G kinase anchoring protein 1
ENC1	1.35	3.23E-04	Ectodermal-neural cortex 1
AK3	1.35	1.47E-04	Adenylate kinase 3
TK1	1.35	5.23E-03	Thymidine kinase 1
KEL	1.36	4.85E-03	Kell metallo-endopeptidase (Kell blood group)
JMJD7	1.36	1.03E-02	Jumonji domain containing 7
ENTPD1	1.36	2.21E-02	Ectonucleoside triphosphate diphosphohydrolase 1
FBXO25	1.36	1.20E-02	F-box protein 25
RPS6KA1	1.36	5.35E-05	Ribosomal protein S6 kinase A1
MEST	1.36	2.99E-03	Mesoderm specific transcript
TMEM14A	1.36	2.13E-02	Transmembrane protein 14A
LSMEM1	1.37	1.40E-02	Leucine rich single-pass membrane protein 1
PGRMC1	1.37	2.59E-02	Progesterone receptor membrane component 1
CD82	1.37	1.33E-02	CD82 molecule
SFRP2	1.37	8.70E-03	Secreted frizzled related protein 2
FBXL21P	1.37	4.86E-04	F-box and leucine rich repeat protein 21
NUDCD2	1.37	1.18E-03	NudC domain containing 2
WDSUB1	1.37	1.96E-03	WD repeat, sterile alpha motif and U-box domain containing 1
TMEM164	1.38	3.64E-04	Transmembrane protein 164
GPR137B	1.39	1.08E-02	G protein-coupled receptor 137B
ANXA2	1.39	1.77E-02	Annexin A2
PPP4R4	1.39	2.12E-02	Protein phosphatase 4 regulatory subunit 4
SEMA4A	1.39	1.56E-03	Semaphorin 4A
DDO	1.39	2.11E-02	D-aspartate oxidase
KRT23	1.39	3.90E-03	Keratin 23
NOC3L	1.39	8.93E-04	NOC3 like DNA replication regulator
FMN1	1.39	1.22E-03	Formin 1
HAUS3	1.40	3.96E-03	HAUS augmin like complex subunit 3
BPHL	1.40	3.34E-02	Biphenyl hydrolase like
PLA1A	1.41	4.31E-02	Phospholipase A1 member A
FN3K	1.41	7.25E-04	Fructosamine 3 kinase
TMEM17	1.41	1.85E-02	Transmembrane protein 17
GPR50	1.41	3.39E-03	G protein-coupled receptor 50
USP46	1.41	6.96E-04	Ubiquitin specific peptidase 46
PRSS35	1.41	3.00E-03	Serine protease 35
ST3GAL5	1.41	2.23E-03	ST3 beta-galactoside alpha-2,3-sialyltransferase 5
FBXL4	1.42	1.56E-03	F-box and leucine rich repeat protein 4
SH3BP1	1.42	8.05E-04	SH3 domain binding protein 1
AIFM2	1.42	2.51E-05	Apoptosis inducing factor mitochondria associated 2
ANXA7	1.42	9.27E-03	Annexin A7

EYA4	1.42	6.45E-04	EYA transcriptional coactivator and phosphatase 4
SNAPC5	1.42	4.20E-03	Small nuclear RNA activating complex polypeptide 5
PDIA2	1.42	2.88E-02	Protein disulfide isomerase family A member 2
MEIKIN	1.42	5.64E-03	Meiotic kinetochore factor
IL10RA	1.43	3.99E-02	Interleukin 10 receptor subunit alpha
LAPTM4A	1.43	9.44E-04	Lysosomal protein transmembrane 4 alpha
C25H11orf52	1.43	3.55E-05	Chromosome 25 C11orf52 homolog
KLHL34	1.43	2.41E-02	Kelch like family member 34
UNC13C	1.43	3.53E-04	Unc-13 homolog C
DNAJC6	1.43	7.83E-03	DnaJ heat shock protein family (Hsp40) member C6
TMEM63A	1.43	1.45E-03	Transmembrane protein 63A
SLC24A2	1.43	3.22E-02	Solute carrier family 24 member 2
TEX45	1.43	4.60E-02	Testis expressed 45
VPS37D	1.43	5.32E-04	VPS37D subunit of ESCRT-I
LPCAT2	1.44	8.88E-05	Lysophosphatidylcholine acyltransferase 2
EIF3J	1.44	8.91E-07	Eukaryotic translation initiation factor 3 subunit J
CDIN1	1.44	1.09E-04	CDAN1 interacting nuclease 1
P2RY8	1.44	5.52E-05	P2Y receptor family member 8
CA4	1.44	6.26E-03	Carbonic anhydrase 4
TVP23A	1.45	4.17E-03	Trans-golgi network vesicle protein 23 homolog A
CLHC1	1.45	6.22E-03	Clathrin heavy chain linker domain containing 1
RNF138	1.45	2.32E-02	Ring finger protein 138
CHN2	1.46	5.47E-03	Chimerin 2
PHGDH	1.46	3.03E-02	Phosphoglycerate dehydrogenase
NLN	1.46	1.34E-04	Neurolysin
NPY2R	1.46	4.60E-04	Neuropeptide Y receptor Y2
PCMTD1	1.47	2.69E-04	Protein-L-isoaspartate (D-aspartate) O-methyltransferase domain containing 1
SGTB	1.47	1.26E-02	Small glutamine rich tetratricopeptide repeat co-chaperone beta
GALNT2	1.47	5.19E-06	Polypeptide N-acetylgalactosaminyltransferase 2
IL7R	1.47	1.29E-02	Interleukin 7 receptor
EXOC5	1.47	5.76E-04	Exocyst complex component 5
SHISA5	1.48	2.32E-03	Shisa family member 5
ASB5	1.48	2.21E-04	Ankyrin repeat and SOCS box containing 5
MRPS18C	1.48	1.89E-02	Mitochondrial ribosomal protein S18C
F13A1	1.48	2.27E-03	Coagulation factor XIII A chain
PTGER4	1.48	7.78E-04	Prostaglandin E receptor 4
VAV3	1.49	3.01E-02	Vav guanine nucleotide exchange factor 3

PLA2G4A	1.50	7.07E-03	Phospholipase A2 group IVA
SUSD4	1.50	4.83E-02	Sushi domain containing 4
PDE10A	1.50	1.17E-03	Phosphodiesterase 10A
POLI	1.50	2.19E-05	DNA polymerase iota
KCNE4	1.50	2.04E-02	Potassium voltage-gated channel subfamily E regulatory subunit 4
MET	1.50	2.47E-03	MET proto-oncogene, receptor tyrosine kinase
PRR5	1.50	9.83E-05	Proline rich 5
OGFRL1	1.50	7.69E-03	Opioid growth factor receptor like 1
ASPN	1.51	1.79E-02	Asporin
SYCE3	1.51	2.07E-02	Synaptonemal complex central element protein 3
ANTXR1	1.51	1.27E-03	ANTXR cell adhesion molecule 1
SASH3	1.51	6.98E-03	SAM and SH3 domain containing 3
ADAL	1.51	3.40E-04	Adenosine deaminase like
TMEM106A	1.51	4.83E-02	Transmembrane protein 106A
STKLD1	1.51	1.07E-04	Serine/threonine kinase like domain containing 1
NUDT12	1.51	1.78E-03	Nudix hydrolase 12
PSMG4	1.51	1.53E-03	Proteasome assembly chaperone 4
PPL	1.51	2.57E-02	Periplakin
ADCY7	1.52	1.79E-02	Adenylate cyclase 7
SLC9A9	1.52	8.26E-04	Solute carrier family 9 member A9
SLC22A4	1.52	2.21E-03	Solute carrier family 22 member 4
MORN3	1.52	9.77E-03	MORN repeat containing 3
SLC2A12	1.52	2.26E-04	Solute carrier family 2 member 12
MKRN2OS	1.52	3.38E-03	MKRN2 opposite strand
PRKAG2	1.53	9.13E-04	Protein kinase AMP-activated non-catalytic subunit gamma 2
TMEM25	1.53	1.94E-02	Transmembrane protein 25
NDC80	1.53	2.58E-02	NDC80 kinetochore complex component
CRISPLD2	1.53	1.07E-02	Cysteine rich secretory protein LCCL domain containing 2
TECRL	1.54	1.39E-03	Trans-2,3-enoyl-CoA reductase like
TMEM97	1.54	7.39E-03	Transmembrane protein 97
ECM2	1.54	9.46E-03	Extracellular matrix protein 2
DLK1	1.54	7.28E-05	Delta like non-canonical Notch ligand 1
AGPAT4	1.54	2.62E-07	1-acylglycerol-3-phosphate O-acyltransferase 4
IGF1	1.54	1.41E-02	Insulin like growth factor 1
SYNM	1.55	3.60E-05	Synemin
SAMD12	1.55	6.56E-04	Sterile alpha motif domain containing 12
CTNNBIP1	1.55	2.75E-09	Catenin beta interacting protein 1
NMNAT1	1.55	4.54E-05	Nicotinamide nucleotide adenylyltransferase 1
TMOD2	1.55	1.94E-02	Tropomodulin 2
IQGAP2	1.55	1.96E-02	IQ motif containing GTPase activating protein 2

SCUBE2	1.55	1.20E-03	Signal peptide, CUB domain and EGF like domain containing 2
RP2	1.56	1.52E-02	RP2 activator of ARL3 GTPase
TMEM241	1.56	6.91E-04	Transmembrane protein 241
TMEM233	1.56	1.11E-03	Transmembrane protein 233
LY75	1.56	1.26E-03	Lymphocyte antigen 75
F5	1.56	3.27E-03	Coagulation factor V
SMOC2	1.57	9.45E-04	SPARC related modular calcium binding 2
PEX11A	1.57	6.91E-04	Peroxisomal biogenesis factor 11 alpha
ARL11	1.58	3.51E-03	ADP ribosylation factor like GTPase 11
CMTM6	1.58	2.19E-04	CKLF like MARVEL transmembrane domain containing 6
SLC7A10	1.58	2.65E-02	Solute carrier family 7 member 10
PGAP4	1.58	1.73E-02	Post-GPI attachment to proteins GalNAc transferase 4
C1R	1.58	9.03E-03	Complement C1r
MYOZ3	1.58	1.16E-02	Myozenin 3
ASB2	1.58	1.28E-04	Ankyrin repeat and SOCS box containing 2
NCF1	1.59	2.26E-02	Neutrophil cytosolic factor 1
SPON1	1.59	2.70E-02	Spondin 1
ARFGEF3	1.59	7.31E-05	ARFGEF family member 3
CACNG4	1.60	1.51E-02	Calcium voltage-gated channel auxiliary subunit gamma 4
OSMR	1.60	4.37E-07	Oncostatin M receptor
GSTT2B	1.61	5.16E-05	Glutathione S-transferase theta 2B
CH25H	1.61	3.18E-02	Cholesterol 25-hydroxylase
TAF1B	1.61	5.19E-03	TATA-box binding protein associated factor, RNA polymerase I subunit B
BLVRA	1.61	5.37E-03	Biliverdin reductase A
SNAI2	1.61	1.81E-03	Snail family transcriptional repressor 2
GNS	1.62	8.52E-04	Glucosamine (N-acetyl)-6-sulfatase
MCRIP1	1.62	2.10E-04	MAPK regulated corepressor interacting protein 1
CMTM3	1.62	1.00E-02	CKLF like MARVEL transmembrane domain containing 3
ZWILCH	1.62	2.58E-02	Zwilch kinetochore protein
MYOF	1.63	1.81E-02	Myoferlin
JAM2	1.63	3.71E-04	Junctional adhesion molecule 2
ARL6	1.63	4.00E-02	ADP ribosylation factor like GTPase 6
FHL5	1.63	2.14E-03	Four and a half LIM domains 5
DACT2	1.63	1.27E-02	Dishevelled binding antagonist of beta catenin 2
VCAN	1.64	2.31E-02	Versican
S100A11	1.64	9.07E-03	S100 calcium binding protein A11
COL21A1	1.64	4.98E-03	Collagen type XXI alpha 1 chain

DIPK1C	1.64	4.18E-03	Divergent protein kinase domain 1C
RGS9	1.64	2.78E-02	Regulator of G protein signaling 9
HELB	1.65	2.68E-02	DNA helicase B
CFTR	1.65	6.35E-05	CF transmembrane conductance regulator
DCLK2	1.65	8.42E-04	Doublecortin like kinase 2
CPQ	1.65	2.51E-05	Carboxypeptidase Q
TMEM123	1.65	1.82E-02	Transmembrane protein 123
CNTNAP1	1.65	5.43E-03	Contactin associated protein 1
RAC2	1.65	1.59E-03	Rac family small GTPase 2
ASNS	1.66	2.29E-03	Asparagine synthetase (glutamine-hydrolyzing)
MINAR1	1.66	1.38E-02	Membrane integral NOTCH2 associated receptor 1
IFIH1	1.66	1.87E-02	Interferon induced with helicase C domain 1
SALL4	1.66	1.33E-02	Spalt like transcription factor 4
E2F3	1.67	1.60E-04	E2F transcription factor 3
DNASE1L3	1.67	5.99E-06	Deoxyribonuclease 1 like 3
STXBP6	1.67	2.30E-02	Syntaxin binding protein 6
COPZ2	1.67	8.57E-03	COPI coat complex subunit zeta 2
PTPN22	1.67	1.10E-03	Protein tyrosine phosphatase non-receptor type 22
PLB1	1.67	2.99E-02	Phospholipase B1
TMEM150A	1.68	2.82E-03	Transmembrane protein 150A
PLEKHA2	1.68	1.28E-05	Pleckstrin homology domain containing A2
C1D	1.68	2.12E-02	C1D nuclear receptor corepressor
DAPP1	1.69	4.05E-03	Dual adaptor of phosphotyrosine and 3-phosphoinositides 1
SELE	1.69	3.59E-02	Selectin E
PIK3CD	1.69	2.02E-04	Phosphatidylinositol-4,5-bisphosphate 3-kinase catalytic subunit delta
TTC32	1.69	9.80E-05	Tetratricopeptide repeat domain 32
HACD4	1.69	1.74E-03	3-hydroxyacyl-CoA dehydratase 4
FAM131B	1.70	1.05E-03	Family with sequence similarity 131 member B
IL15	1.70	3.64E-02	Interleukin 15
SACS	1.70	3.99E-04	Sacsin molecular chaperone
CCBE1	1.70	2.57E-02	Collagen and calcium binding EGF domains 1
ITM2C	1.71	7.32E-03	Integral membrane protein 2C
MEAK7	1.72	4.31E-07	MTOR associated protein, eak-7 homolog
PTPN5	1.72	6.03E-03	Protein tyrosine phosphatase non-receptor type 5
NLRC5	1.72	2.09E-03	NLR family CARD domain containing 5
ARSB	1.72	9.73E-03	Arylsulfatase B
HPSE	1.73	3.31E-03	Heparanase
VEGFD	1.73	3.76E-08	Vascular endothelial growth factor D
BCO1	1.73	1.05E-03	Beta-carotene oxygenase 1
THEMIS2	1.74	7.48E-04	Thymocyte selection associated family member 2

KCNK13	1.74	1.05E-02	Potassium two pore domain channel subfamily K member 13
EPB41L4A	1.74	3.69E-10	Erythrocyte membrane protein band 4.1 like 4A
ANKDD1A	1.74	7.72E-03	Ankyrin repeat and death domain containing 1A
C4H4orf33	1.75	3.78E-03	Chromosome 4 C4orf33 homolog
RETREG1	1.76	5.55E-04	Reticulophagy regulator 1
PGAP1	1.76	1.32E-02	Post-GPI attachment to proteins inositol deacylase 1
DAPK2	1.76	2.59E-03	Death associated protein kinase 2
STAT4	1.76	9.36E-03	Signal transducer and activator of transcription 4
TRAPPC2	1.76	2.76E-03	Trafficking protein particle complex subunit 2
ZFAND2A	1.77	2.04E-02	Zinc finger AN1-type containing 2A
ANKRD1	1.77	2.01E-02	Ankyrin repeat domain 1
LIPA	1.77	8.86E-03	Lipase A, lysosomal acid type
ASB10	1.78	1.42E-07	Ankyrin repeat and SOCS box containing 10
COTL1	1.78	1.43E-03	Coactosin like F-actin binding protein 1
PSD2	1.78	4.69E-02	Pleckstrin and Sec7 domain containing 2
ARL9	1.78	2.45E-04	ADP ribosylation factor like GTPase 9
UPP1	1.79	4.67E-03	Uridine phosphorylase 1
DHRS13	1.79	1.50E-02	Dehydrogenase/reductase 13
RGS10	1.80	5.08E-03	Regulator of G protein signaling 10
CENPW	1.80	1.90E-03	Centromere protein W
GRAP2	1.80	4.39E-02	GRB2 related adaptor protein 2
CA8	1.81	4.40E-05	Carbonic anhydrase 8
STK17B	1.81	1.24E-03	Serine/threonine kinase 17b
BTC	1.82	1.88E-09	Betacellulin
LYPLAL1	1.83	6.46E-03	Lysophospholipase like 1
GJA3	1.83	2.54E-03	Gap junction protein alpha 3
FBLN1	1.84	7.51E-04	Fibulin 1
DUSP28	1.84	1.54E-03	Dual specificity phosphatase 28
ARHGAP22	1.84	7.52E-04	Rho GTPase activating protein 22
IHO1	1.84	1.70E-02	Interactor of HORMAD1 1
DRAXIN	1.85	6.17E-03	Dorsal inhibitory axon guidance protein
ZNF367	1.85	2.81E-05	Zinc finger protein 367
TNFAIP6	1.85	8.90E-03	TNF alpha induced protein 6
RUBCNL	1.85	7.22E-04	Rubicon like autophagy enhancer
SFMBT2	1.86	4.08E-06	Scm like with four mbt domains 2
ITGA4	1.86	9.07E-03	Integrin subunit alpha 4
TNFAIP8	1.86	1.96E-03	TNF alpha induced protein 8
OGN	1.86	5.87E-03	Osteoglycin
BEND6	1.86	1.86E-02	BEN domain containing 6
CEND1	1.86	2.43E-03	Cell cycle exit and neuronal differentiation 1
GCG	1.87	4.74E-02	Glucagon

TTC7A	1.87	7.31E-04	Tetratricopeptide repeat domain 7A
TMEM38B	1.88	6.89E-05	Transmembrane protein 38B
FGL2	1.88	4.19E-04	Fibrinogen like 2
PTPN6	1.88	1.31E-05	Protein tyrosine phosphatase non-receptor type 6
ZP1	1.88	3.43E-02	Zona pellucida glycoprotein 1
PLEK	1.88	3.33E-02	Pleckstrin
PRKCB	1.89	2.02E-05	Protein kinase C beta
LOXHD1	1.89	1.59E-02	Lipoxygenase homology PLAT domains 1
FAM149A	1.89	9.68E-03	Family with sequence similarity 149 member A
SS18L1	1.90	6.41E-04	SS18L1 subunit of BAF chromatin remodeling complex
NIBAN1	1.90	1.52E-05	Niban apoptosis regulator 1
BMP6	1.91	2.75E-06	Bone morphogenetic protein 6
PI16	1.92	1.13E-03	Peptidase inhibitor 16
ASB14	1.92	3.07E-04	Ankyrin repeat and SOCS box containing 14
SERPING1	1.93	8.42E-09	Serpin family G member 1
BCAT1	1.93	1.55E-02	Branched chain amino acid transaminase 1
B3GALT5	1.94	7.02E-04	Beta-1,3-galactosyltransferase 5
ANKDD1B	1.94	2.97E-02	Ankyrin repeat and death domain containing 1B
CR1	1.94	3.52E-04	Complement C3b/C4b receptor 1 (Knops blood group)
COL8A1	1.94	5.42E-07	Collagen type VIII alpha 1 chain
CALM2	1.95	1.26E-04	Calmodulin 2
CRACR2A	1.95	4.88E-03	Calcium release activated channel regulator 2A
PPARG	1.97	5.83E-03	Peroxisome proliferator activated receptor gamma
PCOLCE2	1.97	7.75E-04	Procollagen C-endopeptidase enhancer 2
PLCB2	1.98	8.73E-07	Phospholipase C beta 2
YPEL1	1.99	1.25E-05	Yippee like 1
EFCC1	1.99	5.28E-09	EF-hand and coiled-coil domain containing 1
RBM47	2.00	3.20E-03	RNA binding motif protein 47
RASSF5	2.00	6.60E-04	Ras association domain family member 5
SNX20	2.01	8.31E-03	Sorting nexin 20
MXRA5	2.01	5.37E-03	Matrix remodeling associated 5
IL13RA1	2.01	5.35E-03	Interleukin 13 receptor subunit alpha 1
STUM	2.01	7.05E-05	Stum, mechanosensory transduction mediator homolog
KNTC1	2.02	3.33E-02	Kinetochore associated 1
MAP3K21	2.02	2.57E-02	Mitogen-activated protein kinase kinase kinase 21
CHAC1	2.02	6.43E-12	ChaC glutathione specific gamma-glutamylcyclotransferase 1
CDC14B	2.02	4.53E-04	Cell division cycle 14B
PGPEP1L	2.02	2.70E-04	Pyroglutamyl-peptidase I like
LHFPL2	2.03	8.07E-09	LHFPL tetraspan subfamily member 2

TBX20	2.03	2.84E-05	T-box transcription factor 20
GCNT4	2.04	1.61E-02	Glucosaminyl (N-acetyl) transferase 4
RIMS1	2.04	3.73E-04	Regulating synaptic membrane exocytosis 1
MYLK4	2.04	5.69E-09	Myosin light chain kinase family member 4
POF1B	2.04	3.13E-03	POF1B actin binding protein
ANKRD33B	2.04	1.66E-02	Ankyrin repeat domain 33B
ARNTL	2.06	3.32E-06	Aryl hydrocarbon receptor nuclear translocator like
FGF14	2.06	3.69E-03	Fibroblast growth factor 14
CYTH4	2.07	1.40E-03	Cytohesin 4
B2M	2.07	1.15E-09	Beta-2-microglobulin
RYR3	2.08	1.69E-05	Ryanodine receptor 3
LITAF	2.08	5.88E-03	Lipopolysaccharide induced TNF factor
SLC41A2	2.08	9.54E-03	Solute carrier family 41 member 2
PEAK3	2.08	1.70E-03	PEAK family member 3
TPD52	2.09	3.56E-03	Tumor protein D52
PIP5K1B	2.09	9.86E-05	Phosphatidylinositol-4-phosphate 5-kinase type 1 beta
NETO2	2.09	3.51E-03	Neuropilin and tolloid like 2
CYSLTR1	2.10	2.23E-02	Cysteinyl leukotriene receptor 1
RBP7	2.10	3.78E-03	Retinol binding protein 7
FLVCR2	2.10	1.40E-02	FLVCR heme transporter 2
SYNGR3	2.11	7.82E-04	Synaptogyrin 3
PRR5L	2.11	1.21E-03	Proline rich 5 like
GABRB2	2.11	1.45E-02	Gamma-aminobutyric acid type A receptor subunit beta2
CFAP92	2.12	8.18E-04	Cilia and flagella associated protein 92 (putative)
PODN	2.12	6.49E-08	Podocan
ARHGAP9	2.12	4.65E-03	Rho GTPase activating protein 9
MFSD4B	2.14	6.67E-08	Major facilitator superfamily domain containing 4B
TMEM132C	2.14	1.92E-05	Transmembrane protein 132C
LAPTM5	2.14	1.59E-05	Lysosomal protein transmembrane 5
CARD9	2.14	2.90E-04	Caspase recruitment domain family member 9
ACKR4	2.14	5.64E-06	Atypical chemokine receptor 4
DCLK1	2.14	1.86E-05	Doublecortin like kinase 1
P2RY13	2.15	2.29E-02	Purinergic receptor P2Y13
LRRC2	2.15	3.04E-06	Leucine rich repeat containing 2
CSF1R	2.15	1.20E-06	Colony stimulating factor 1 receptor
GPD2	2.16	2.75E-04	Glycerol-3-phosphate dehydrogenase 2
SLC16A9	2.16	9.21E-09	Solute carrier family 16 member 9
TLR1-A	2.17	2.41E-02	Toll-like receptor 1
GSDMA	2.18	7.56E-03	Gasdermin A

TLR3	2.19	2.15E-02	Toll like receptor 3
TBX18	2.20	4.64E-04	T-box transcription factor 18
PPP1R27	2.21	1.48E-05	Protein phosphatase 1 regulatory subunit 27
NXPH2	2.21	1.76E-06	Neurexophilin 2
APELA	2.21	1.12E-03	Apelin receptor early endogenous ligand
PTAFR	2.22	1.25E-03	Platelet activating factor receptor
GRIN3B	2.22	4.89E-03	Glutamate ionotropic receptor NMDA type subunit 3B
HEBP1	2.23	3.19E-03	Heme binding protein 1
ARHGAP25	2.24	3.37E-05	Rho GTPase activating protein 25
MZB1	2.24	2.20E-02	Marginal zone B and B1 cell specific protein
MB	2.24	7.34E-11	Myoglobin
DCHS2	2.25	2.89E-02	Dachsous cadherin-related 2
MKNK1	2.26	2.55E-14	MAPK interacting serine/threonine kinase 1
MILR1	2.26	5.72E-03	Mast cell immunoglobulin like receptor 1
MBOAT2	2.26	1.36E-06	Membrane bound O-acyltransferase domain containing 2
AKAP6	2.26	2.33E-10	A-kinase anchoring protein 6
STARD5	2.26	2.57E-03	StAR related lipid transfer domain containing 5
PDE4D	2.27	1.30E-08	Phosphodiesterase 4D
CCDC59	2.27	2.31E-02	Coiled-coil domain containing 59
WSCD1	2.27	4.18E-05	WSC domain containing 1
MTERF2	2.28	7.64E-06	Mitochondrial transcription termination factor 2
SHOC1	2.28	7.16E-03	Shortage in chiasmata 1
CNTN6	2.29	6.58E-05	Contactin 6
PTPN7	2.29	9.17E-04	Protein tyrosine phosphatase non-receptor type 7
CA5A	2.30	1.83E-03	Carbonic anhydrase 5A
CHRFAM7A	2.31	3.20E-02	CHRNA7 (exons 5-10) and FAM7A (exons A-E) fusion
SLC16A7	2.32	5.81E-03	Solute carrier family 16 member 7
LRRC8B	2.32	4.96E-04	Leucine rich repeat containing 8 VRAC subunit B
PNPLA1	2.34	1.04E-02	Patatin like phospholipase domain containing 1
RASSF2	2.35	2.92E-06	Ras association domain family member 2
LYN	2.35	7.13E-04	LYN proto-oncogene, Src family tyrosine kinase
FAR2	2.35	3.92E-06	Fatty acyl-CoA reductase 2
ANGPT1	2.36	2.77E-04	Angiopoietin 1
SCIN	2.37	1.77E-03	Scinderin
CDC42EP3	2.39	2.61E-06	CDC42 effector protein 3
TMEM26	2.39	1.15E-06	Transmembrane protein 26
IL2RG	2.39	1.47E-03	Interleukin 2 receptor subunit gamma
MFNG	2.40	2.68E-03	MFNG O-fucosylpeptide 3-beta-N-acetylglucosaminyltransferase
C1QB	2.40	1.91E-08	Complement C1q B chain

SH2D1A	2.41	3.94E-02	SH2 domain containing 1A
MAP1LC3C	2.42	5.85E-05	Microtubule associated protein 1 light chain 3 gamma
CSMD3	2.42	3.93E-03	CUB and Sushi multiple domains 3
CDKN2C	2.44	2.47E-16	Cyclin dependent kinase inhibitor 2C
PTPRO	2.44	3.84E-03	Protein tyrosine phosphatase receptor type O
TRIM14	2.45	6.25E-03	Tripartite motif containing 14
PTK2B	2.46	1.41E-04	Protein tyrosine kinase 2 beta
CD79B	2.46	3.75E-03	CD79b molecule
SLA	2.49	2.33E-02	Src like adaptor
PRIMPOL	2.49	1.49E-05	Primase and DNA directed polymerase
MTHFD1L	2.49	6.47E-12	Methylenetetrahydrofolate dehydrogenase (NADP+ dependent) 1 like
PSPH	2.49	7.64E-07	Phosphoserine phosphatase
PSTPIP1	2.50	7.60E-04	Proline-serine-threonine phosphatase interacting protein 1
DCX	2.50	2.86E-06	Doublecortin
GLIPR2	2.50	2.99E-03	GLI pathogenesis related 2
DOCK8	2.51	2.79E-04	Dedicator of cytokinesis 8
C1QTNF8	2.51	2.97E-02	C1q and TNF related 8
GABRA1	2.52	2.35E-06	Gamma-aminobutyric acid type A receptor subunit alpha1
PP2D1	2.52	3.92E-03	Protein phosphatase 2C like domain containing 1
SLC24A4	2.53	2.91E-02	Solute carrier family 24 member 4
RNASET2	2.53	3.67E-04	Ribonuclease T2
DRAM1	2.53	3.28E-02	DNA damage regulated autophagy modulator 1
C5H14orf180	2.53	4.65E-05	Chromosome 5 C14orf180 homolog
LPAR6	2.55	1.14E-03	Lysophosphatidic acid receptor 6
BLK	2.55	2.76E-02	BLK proto-oncogene, Src family tyrosine kinase
PKD1L2	2.56	4.49E-02	Polycystin 1 like 2
NECAB1	2.58	1.96E-04	N-terminal EF-hand calcium binding protein 1
B3GNT5	2.59	3.69E-04	UDP-GlcNAc:betaGal beta-1,3-N-acetylglucosaminyltransferase 5
FSD1L	2.59	1.86E-07	Fibronectin type III and SPRY domain containing 1 like
NOX4	2.61	6.57E-16	NADPH oxidase 4
TBC1D9	2.61	8.89E-08	TBC1 domain family member 9
PIK3R5	2.62	8.55E-07	Phosphoinositide-3-kinase regulatory subunit 5
ATG4A	2.62	1.49E-02	Autophagy related 4A cysteine peptidase
TNFRSF9	2.64	2.61E-02	TNF receptor superfamily member 9
C11H15orf48	2.66	1.62E-03	Chromosome 11 C15orf48 homolog
CAPRIN2	2.66	8.52E-10	Caprin family member 2

HMGCLL1	2.66	2.37E-06	3-hydroxymethyl-3-methylglutaryl-CoA lyase like 1
C1QC	2.66	1.41E-05	Complement C1q C chain
GRIA4	2.67	3.69E-02	Glutamate ionotropic receptor AMPA type subunit 4
NT5DC1	2.67	1.44E-08	5'-nucleotidase domain containing 1
TTC29	2.67	7.96E-03	Tetratricopeptide repeat domain 29
ARHGAP15	2.67	2.85E-03	Rho GTPase activating protein 15
LY86	2.68	1.79E-05	Lymphocyte antigen 86
PPM1H	2.69	2.42E-15	Protein phosphatase, Mg2+/Mn2+ dependent 1H
RHOH	2.69	3.64E-03	Ras homolog family member H
PMAIP1	2.70	1.03E-02	Phorbol-12-myristate-13-acetate-induced protein 1
ANXA1	2.72	2.04E-03	Annexin A1
MARCO	2.72	2.23E-03	Macrophage receptor with collagenous structure
GPR174	2.73	4.63E-04	G protein-coupled receptor 174
IKZF1	2.73	6.07E-05	IKAROS family zinc finger 1
LAMP3	2.73	2.10E-03	Lysosomal associated membrane protein 3
TRAF5	2.73	2.63E-03	TNF receptor associated factor 5
MCTP1	2.75	1.25E-03	Multiple C2 and transmembrane domain containing 1
PTPN3	2.75	2.05E-08	Protein tyrosine phosphatase non-receptor type 3
RAB32	2.76	5.01E-04	RAB32, member RAS oncogene family
LACC1	2.76	5.92E-03	Laccase domain containing 1
CARD11	2.77	5.03E-06	Caspase recruitment domain family member 11
TBXAS1	2.77	3.76E-04	Thromboxane A synthase 1
LACTBL1	2.77	1.90E-03	Lactamase beta like 1
CD44	2.78	6.03E-04	CD44 molecule (Indian blood group)
TNFRSF8	2.79	3.84E-02	TNF receptor superfamily member 8
CORO2A	2.79	6.29E-05	Coronin 2A
MMP23B	2.80	1.52E-05	Matrix metallopeptidase 23B
TSPO2	2.81	9.45E-03	Translocator protein 2
OMD	2.81	2.49E-02	Osteomodulin
GALNT6	2.82	1.13E-03	Polypeptide N-acetylgalactosaminyltransferase 6
IL2RB	2.83	3.52E-05	Interleukin 2 receptor subunit beta
PLXNC1	2.83	1.44E-04	Plexin C1
COL28A1	2.83	3.40E-27	Collagen type XXVIII alpha 1 chain
TLR2	2.83	1.76E-02	Toll like receptor 2
ARAP2	2.83	7.19E-04	ArfGAP with RhoGAP domain, ankyrin repeat and PH domain 2
CTXN1	2.84	9.12E-06	Cortexin 1
HSPB2	2.85	1.21E-04	Heat shock protein family B (small) member 2
EMB	2.86	8.12E-03	Embigin

TLR4	2.86	3.90E-04	Toll like receptor 4
CD80	2.89	3.39E-03	CD80 molecule
PRDM1	2.89	3.72E-04	PR/SET domain 1
PRG4	2.91	3.44E-03	Proteoglycan 4
ABCB1	2.92	2.57E-09	ATP binding cassette subfamily B member 1
LCP2	2.93	1.62E-04	Lymphocyte cytosolic protein 2
RGS1	2.94	9.28E-03	Regulator of G protein signaling 1
ABI3	2.95	6.30E-03	ABI family member 3
PAK1	2.97	1.55E-05	P21 (RAC1) activated kinase 1
PRKCQ	3.00	1.34E-09	Protein kinase C theta
IKZF3	3.00	1.05E-03	IKAROS family zinc finger 3
GPNMB	3.01	1.97E-02	Glycoprotein nmb
EVI2B	3.02	2.30E-04	Ecotropic viral integration site 2B
PLCXD1	3.05	1.67E-02	Phosphatidylinositol specific phospholipase C X domain containing 1
GPR160	3.05	4.05E-02	G protein-coupled receptor 160
DSCAML1	3.06	7.21E-04	DS cell adhesion molecule like 1
ACSL5	3.06	2.53E-02	Acyl-CoA synthetase long chain family member 5
NCF4	3.06	3.97E-03	Neutrophil cytosolic factor 4
EOMES	3.07	2.65E-02	Eomesodermin
LPAR5	3.07	4.23E-04	Lysophosphatidic acid receptor 5
CD247	3.11	8.39E-07	CD247 molecule
FYB1	3.12	3.36E-04	FYN binding protein 1
BANK1	3.12	5.60E-03	B cell scaffold protein with ankyrin repeats 1
CCL22	3.12	3.59E-02	C-C motif chemokine ligand 22
DNASE2B	3.13	8.19E-06	Deoxyribonuclease 2 beta
LRRC17	3.14	1.90E-03	Leucine rich repeat containing 17
CHRNB4	3.20	9.19E-04	Cholinergic receptor nicotinic beta 4 subunit
SFRP1	3.21	3.83E-05	Secreted frizzled related protein 1
LRRN3	3.22	7.93E-03	Leucine rich repeat neuronal 3
ESM1	3.22	2.16E-04	Endothelial cell specific molecule 1
TMEM156	3.24	5.30E-03	Transmembrane protein 156
C15H16orf89	3.24	2.02E-02	Chromosome 15 C16orf89 homolog
FOXI1	3.24	8.52E-04	Forkhead box I1
CASR	3.25	7.80E-06	Calcium sensing receptor
KCNH7	3.25	4.77E-06	Potassium voltage-gated channel subfamily H member 7
C1QA	3.26	8.87E-07	Complement C1q A chain
CD74	3.26	3.13E-23	CD74 molecule
FGF6	3.26	4.92E-09	Fibroblast growth factor 6
TLR7	3.27	3.29E-04	Toll like receptor 7
LCP1	3.27	2.69E-07	Lymphocyte cytosolic protein 1
MAP1B	3.28	1.87E-17	Microtubule associated protein 1B

NQO2	3.30	1.10E-10	N-ribosyldihydronicotinamide:quinone reductase 2
BLNK	3.30	2.82E-05	B cell linker
MGAT4D	3.30	4.30E-02	MGAT4 family member D
MPEG1	3.31	7.37E-10	Macrophage expressed 1
CD4	3.32	4.64E-03	CD4 molecule
C3AR1	3.33	1.36E-04	Complement C3a receptor 1
C1S	3.34	7.17E-06	Complement C1s
ITGB2	3.35	3.47E-06	Integrin subunit beta 2
GPR34	3.36	7.18E-05	G protein-coupled receptor 34
KCNQ3	3.36	7.46E-12	Potassium voltage-gated channel subfamily Q member 3
CD5	3.36	1.09E-02	CD5 molecule
FASLG	3.37	7.04E-03	Fas ligand
GDF6	3.39	4.62E-03	Growth differentiation factor 6
CFH	3.44	1.64E-15	Complement factor H
IL18R1	3.47	4.24E-04	Interleukin 18 receptor 1
EVI2A	3.49	6.01E-03	Ecotropic viral integration site 2A
TEX30	3.50	1.97E-07	Testis expressed 30
SAMSN1	3.51	1.70E-03	SAM domain, SH3 domain and nuclear localization signals 1
LAPTM4B	3.53	4.71E-03	Lysosomal protein transmembrane 4 beta
GREM1	3.54	1.30E-05	Gremlin 1, DAN family BMP antagonist
IGFN1	3.54	2.18E-04	Immunoglobulin like and fibronectin type III domain containing 1
CST7	3.55	8.81E-03	Cystatin F
TSHR	3.55	3.61E-03	Thyroid stimulating hormone receptor
NLRC3	3.56	6.85E-07	NLR family CARD domain containing 3
CCL19	3.56	1.60E-02	C-C motif chemokine ligand 19
CDKN2B	3.58	8.67E-03	Cyclin dependent kinase inhibitor 2B
ADGRG5	3.58	4.43E-04	Adhesion G protein-coupled receptor G5
TNFSF13B	3.59	1.83E-03	TNF superfamily member 13b
ACSL6	3.60	4.27E-10	Acyl-CoA synthetase long chain family member 6
LCK	3.62	1.01E-04	LCK proto-oncogene, Src family tyrosine kinase
NFAM1	3.63	5.80E-05	NFAT activating protein with ITAM motif 1
SLCO4C1	3.64	2.85E-02	Solute carrier organic anion transporter family member 4C1
RUNX3	3.65	1.60E-04	RUNX family transcription factor 3
STING1	3.66	7.65E-06	Stimulator of interferon response cGAMP interactor 1
WDFY4	3.69	1.19E-06	WDFY family member 4
CD8A	3.71	1.73E-04	CD8a molecule

OTULINL	3.72	5.31E-06	OTU deubiquitinase with linear linkage specificity like
BCL2L14	3.73	3.12E-02	BCL2 like 14
CTSS	3.74	5.69E-07	Cathepsin S
PTPRC	3.75	1.20E-05	Protein tyrosine phosphatase receptor type C
AOAH	3.76	7.85E-04	Acyloxyacyl hydrolase
TNFSF8	3.77	2.31E-02	TNF superfamily member 8
RPL3L	3.78	9.21E-11	Ribosomal protein L3 like
BCL11B	3.78	6.14E-08	BAF chromatin remodeling complex subunit BCL11B
ALDH1L2	3.79	2.45E-16	Aldehyde dehydrogenase 1 family member L2
POU2AF1	3.81	2.67E-05	POU class 2 homeobox associating factor 1
SPI1	3.82	7.19E-06	Spi-1 proto-oncogene
CD180	3.82	3.20E-02	CD180 molecule
PRTG	3.83	5.76E-06	Protogenin
ATP6V1C2	3.88	1.28E-02	ATPase H+ transporting V1 subunit C2
PDCD1	3.88	8.35E-04	Programmed cell death 1
EPHA1	3.89	3.33E-02	EPH receptor A1
GUCY2F	4.07	1.20E-02	Guanylate cyclase 2F, retinal
THEMIS	4.08	6.25E-03	Thymocyte selection associated
CD83	4.10	2.09E-06	CD83 molecule
TMEM271	4.11	1.83E-02	Transmembrane protein 271
GPR65	4.11	3.49E-05	G protein-coupled receptor 65
LCAT	4.13	2.97E-06	Lecithin-cholesterol acyltransferase
TSPAN8	4.17	1.59E-02	Tetraspanin 8
SCEL	4.18	7.75E-06	Sciellin
IGSF6	4.23	1.92E-03	Immunoglobulin superfamily member 6
TFCP2L1	4.27	5.91E-13	Transcription factor CP2 like 1
TTC34	4.27	2.09E-04	Tetratricopeptide repeat domain 34
SUCNR1	4.31	7.38E-03	Succinate receptor 1
CNPY1	4.32	1.40E-02	Canopy FGF signaling regulator 1
DOCK2	4.32	1.33E-06	Dedicator of cytokinesis 2
ZNF831	4.33	1.80E-04	Zinc finger protein 831
KLHDC8B	4.37	4.18E-09	Kelch domain containing 8B
LY96	4.38	2.01E-03	Lymphocyte antigen 96
CIITA	4.40	2.70E-06	Class II major histocompatibility complex transactivator
CCR7	4.45	9.30E-05	C-C motif chemokine receptor 7
TNFRSF18	4.47	6.49E-05	TNF receptor superfamily member 18
CNRIP1	4.51	7.24E-04	Cannabinoid receptor interacting protein 1
SRMS	4.53	2.86E-05	Src-related kinase lacking C-terminal regulatory tyrosine and N-terminal myristylation sites

UBASH3A	4.54	5.36E-05	Ubiquitin associated and SH3 domain containing A
ITK	4.57	1.06E-04	IL2 inducible T cell kinase
PLD4	4.65	2.06E-03	Phospholipase D family member 4
BCL2A1	4.68	1.01E-03	BCL2 related protein A1
SLC7A9	4.72	9.72E-03	Solute carrier family 7 member 9
CCN6	4.75	2.05E-03	Cellular communication network factor 6
SLC2A6	4.77	3.91E-04	Solute carrier family 2 member 6
IL18	4.78	2.49E-04	Interleukin 18
DEUP1	4.94	1.14E-02	Deuterosome assembly protein 1
TFEC	4.97	5.34E-04	Transcription factor EC
CX3CR1	4.97	1.36E-03	C-X3-C motif chemokine receptor 1
CCL26	5.06	6.62E-04	C-C motif chemokine ligand 26
TXK	5.20	1.01E-03	TXK tyrosine kinase
DHRS9	5.24	2.90E-15	Dehydrogenase/reductase 9
IRF4	5.25	4.28E-03	Interferon regulatory factor 4
CD8B	5.28	3.27E-03	CD8b molecule
GPR55	5.28	1.62E-03	G protein-coupled receptor 55
PKIB	5.42	1.56E-04	CAMP-dependent protein kinase inhibitor beta
LIPI	5.59	6.29E-03	Lipase I
BCL2L15	5.63	1.53E-03	BCL2 like 15
CD40LG	5.67	8.48E-04	CD40 ligand
ACOD1	5.71	2.39E-03	Aconitate decarboxylase 1
XCR1	5.74	1.52E-03	X-C motif chemokine receptor 1
CD6	5.77	3.21E-04	CD6 molecule
LAG3	5.89	6.39E-04	Lymphocyte activating 3
CCK	5.95	3.33E-10	Cholecystokinin
TMEM273	6.15	2.61E-04	Transmembrane protein 273
CD3E	6.38	1.17E-06	CD3e molecule
KCNG4	6.42	1.65E-04	Potassium voltage-gated channel modifier subfamily G member 4
CD2	6.58	1.49E-07	CD2 molecule
CXCR6	6.63	3.92E-07	C-X-C motif chemokine receptor 6
FLT3	6.73	4.53E-05	Fms related receptor tyrosine kinase 3
FAM3B	6.89	1.61E-02	FAM3 metabolism regulating signaling molecule B
ALDH1A1	7.61	1.11E-02	Aldehyde dehydrogenase 1 family member A1
METTL21C	9.10	1.04E-25	Methyltransferase 21C, AARS1 lysine
JCHAIN	11.33	1.20E-05	Joining chain of multimeric IgA and IgM
