# Supplementary material for: Dynamic transcriptomic landscape of myogenesis in Muscovy ducks (Cairina moschata): integrative analysis of hub genes post-hatching
Source: Anim Biosci. 2025 Aug 12;39(1):250159. doi: 10.5713/ab.25.0159 (PMC12754469; doi:10.5713/ab.25.0159)
Supplement: Supplementary file 2 [file ab-25-0159-Supplementary-2.pdf]

Supplemental file 2. GO enrichment analysis for up-regulated genes in 80D					
Term	Enrichment	Count	P Value	Group	Genes
Immune response	5.180068	27	1.61E-12	BP	LOC101800757, LOC106015872, FASLG, CXCR6, CST7, TNFSF13B, LOC101791385, TNFSF10, CCR7, LOC101791569, CCL19, ITGB6, B2M, CD74, CCL22, LOC101800429, LOC101802825, IL15, CD180, IL18, LY86, PRG4, LOC101802909, CD40LG, LOC101798888, TNFSF8, CCL26
Intracellular signal transduction	1.961178	23	2.90E-03	BP	VAV3, ITK, UNC13C, PLEK, PRKAG2, DCLK2, ARHGAP29, ADCY7, GUCY2F, LOC119714503, LOC101795007, ASB10, CHN2, LOC101798888, RPS6KA1, DCX, BLNK, PRKCQ, ASB5, ASB2, JAK2, RGS9, DGKH
Inflammatory response	5.273954	17	3.70E-08	BP	LOC110351185, ANXA1, LOC101800429, LOC101800757, PTAFR, TLR1-A, IL18, CXCR6, PTGS2, CD40LG, TLR7, CCR7, LOC101791359, ITGB6, TLR4, TLR3, TLR2
Cell adhesion	2.209232	15	7.10E-03	BP	SPON2, SPON1, TNFAIP6, ITGA4, CNTN6, FN1, PRTG, KITLG, GPNMB, LOC101799623, STAB1, NINJ1, ITGAV, EMB, CD44
Innate immune response	5.744003	13	8.61E-07	BP	LOC110351185, SPON2, TLR1-A, LY96, LOC101792171, IFIH1, LOC119714503, LOC101804009, TRIM14, TLR7, TLR4, TLR3, TLR2
Chemotaxis	6.168856	11	4.00E-06	BP	CX3CR1, CYSLTR1, XCR1, LOC101800429, LOC101800757, PTAFR, ACKR4, RAC2, LOC101800940, CCR7, CXCR6
Regulation of apoptotic process	2.67813	9	1.70E-02	BP	TNFAIP8, RASSF2, CARD9, TRAF5, LOC101797616, TRAF1, BCL2L14, CARD11, BCL2L15

MyD88-dependent toll-like receptor signaling pathway	7.290466	6	7.77E-04	BP	LOC110351185, LOC101804009, TLR1-A, TLR7, TLR4, TLR2
Positive regulation of NF-kappaB transcription factor activity	3.36483	6	2.92E-02	BP	GREM1, TRAF5, TLR1-A, TRAF1, TLR4, CARD11
Regulation of cytokine production	7.290466	5	3.26E-03	BP	LOC110351185, LOC101804009, TLR1-A, UBASH3A, TLR2
Positive regulation of interferon-gamma production	6.075388	5	6.88E-03	BP	LOC101794055, TXK, IL18, TLR7, TLR4
Positive regulation of inflammatory response	6.075388	5	6.88E-03	BP	PTGER4, SUCNR1, TLR1-A, TLR4, TLR3
Adaptive immune response	4.556541	5	2.04E-02	BP	ITK, LOC101789604, CD4, CTSS, LOC101792606
Phagocytosis	5.302157	4	3.48E-02	BP	ITGB2, NCF4, PLD4, TLR4
Positive regulation of interleukin-6 production	5.302157	4	3.48E-02	BP	SPON2, LOC119714503, TLR7, TLR4
Cellular response to mechanical stimulus	4.86031	4	4.40E-02	BP	PTGER4, ANKRD1, TLR7, TLR4
Positive regulation of neutrophil apoptotic process	14.58093	3	1.34E-02	BP	ANXA1, PIK3CD, PIK3CB

Detection of lipopolysaccharide	14.58093	3	1.34E-02	BP	SCARB1, LY96, TLR4
Positive regulation of platelet activation	14.58093	3	1.34E-02	BP	PLA2G4A, JAK2, TLR4
Toll-like receptor 2 signaling pathway	10.9357	3	2.56E-02	BP	LOC110351185, LOC101804009, TLR2
Response to molecule of bacterial origin	10.9357	3	2.56E-02	BP	LOC110351185, LOC101804009, TLR2
Immune system process	10.9357	3	2.56E-02	BP	LOC101795007, INPP5D, LOC101798888
Positive regulation of receptor internalization	8.748559	3	4.07E-02	BP	GREM1, LOC119714503, ANGPT1
Positive regulation of monocyte chemotaxis	8.748559	3	4.07E-02	BP	LOC101800429, LOC101800757, CXCR6

Integral component of membrane	1.243267	257	5.34E-06	CC	ARL6IP1, PLEKHB2, TMEM200A, PGAP4, TMEM97, AQP3, ANTXR1, GPR174, KRTCAP3, RASSF5, LOC101801566, WLS, ENTPD1, ST6GAL1, KCNK13, ACSL5, BTC, CH25H, LOC101796588, KEL, NINJ1, GPR160, MXRA5, OTULINL, SLC22A4, TMEM63A, ABCB1, SLC35D2, MGST1, LOC101797680, CEND1, TMTC2, PPP1R3A, HACD4, PIK3R5, CD79B, LOC101794055, TSPAN8, TSPAN9, ST8SIA4, ST3GAL5, LOC101789696, STXBP6, XCR1, LRRN3, NETO2, IGF1, GPR137B, MARCO, LOC101799758, LPAR5, LPAR6, NOX4, CHRFAM7A, TNFSF8, LOC101791359, ITM2C, LOC101791918, LOC101803429, PTPRO, CD3E, SLC7A10, LOC101791362, PIP4P2, FLVCR2, CCR7, EMB, RNF150, STUM, GPR34, ELOVL5, NFAM1, MYOF, SLC2A12, MMEL1, ANO5, LOC106015064, TMEM37, SLC7A9, B3GNT5, LOC101800940, TLR7, TLR4, CD44, TLR3, TLR2, PTGER4, SAMD8, MARCHF1, PTAFR, CCDC107, CXCR6, ADCY7, GUCY2F, TMEM164, LOC101794893, GPNMB, SUSD4, TM4SF18, CD74, IL10RA, GPR55, LAPTM5, GPR50, LOC101799795, LHFPL2, LOC101799791, TMEM156, KITLG, CD40LG, DPY19L3, OSTM1, SLCO4C1, LPCAT2, LOC101798906, PNPLA4, LOC101801378, IL18R1, SCARB1, LOC101792808, NRROS, LOC101802936, CD83, ATP8A1, CD82, GOLT1B, LOC101800757, CD80, GPR65, NPY2R, LY75, GALNT10, TMEM144, LAPTM4B, LAPTM4A, FAM174B, LAMP3, TNFRSF8, ENPP4, SLC25A43, SLC16A9, PLXNC1, TMEM38B, TMEM14A, IL13RA1, MINAR1, LOC101800628, SLC38A1, LAG3, CHRNB4, LOC101789505, CASR, KCNH7, SPNS3, CD180, TLR1-A, SHISA5, MPEG1, LOC101793233, LOC101804544, CD8B, CD8A, SLC9A8, SLC9A9, LOC101803217, LOC101790642, CD274, CSF1R, DIPK1C, SUCNR1, SLC41A2, LSMEM1, PLD4, TMEM123, TMEM26, LOC101796079, CALHM2, ADGRG5,

					ADGRG6, LOC101789730, GRIA4, TRAPPC2, LOC101789606, TMEM120B, TMEM132C, LOC101789604, SLC37A1, SRD5A3, TVP23A, B3GALT5, TECRL, LOC101796089, TMEM17, LOC101795552, LOC101791991, TBXAS1, LOC101790424, LOC101793812, IL7R, CNIH4, LOC101792606, LOC110351185, MCTP1, PODXL2, FLT3, LOC101791081, LOC101792171, SLC2A6, TNFSF13B, LOC119718657, LOC101792733, LOC101800311, LOC101803387, ABCC4, LOC101800429, TNFRSF18, SYNGR3, LOC101799374, LOC101801190, C22H1ORF159, RP9, LOC101800783, MET, CNTNAP5, SLC24A2, CX3CR1, RNFT2, FGL2, IL2RG, AGPAT4, PRTG, P2RY8, LOC113840251, DRAM1, LOC101802995, LOC101800696, LOC101799949, LOC101801783, CMTM3, GALNT2, MBOAT2, HS3ST5, TSHR, DLK1, SGPP1, CD2, TSPO2, LOC101797774, CD4, TSPAN13, IL2RB, LOC101804009, ACKR4, ASB5, CD247, PDCD1, PTPN5
Extracellular space	2.386095	33	6.01E-06	CC	SPON2, SERPINC1, PLAT, CTSS, CLEC3B, LOC101791385, LOC101799623, LOC101791569, CCL19, LOC101796956, EDN1, CCL22, ANGPT1, LOC101802825, IL15, MMP2, IL18, VEGFD, GCG, MMEL1, IGF1, LOC101802909, GREM1, SFRP1, KITLG, CD40LG, LOC101795437, LOC101799613, TNFSF8, LOC101795637, LOC101801552, ANGPTL1, CCL26
Lysosome	2.914243	9	1.05E-02	CC	SCARB1, DRAM1, RRAGD, HEXA, LAPTM5, TLR7, TMEM97, GNS, CTSS

T cell receptor complex	14.89502	3	1.29E-02	CC	LOC119714503, LOC101798888, LOC101792606
Extracellular region	1.424741	44	1.56E-02	CC	DRAXIN, C1QA, SPON2, SPON1, NXPH2, CPXM2, LOC101800358, LOC106015872, FBLN1, PLAT, C1QTNF3, ESM1, C1QTNF8, GLIPR2, LIPI, CCN6, TNFSF10, FAM3B, FAM3C, B2M, EDN1, ANXA2, MMP2, FN1, EPDR1, PRG4, GCG, CCK, LOC101798784, LOC101798586, ECRG4, BMP6, ASPN, SMOC2, SFRP1, KITLG, FGF14, SFRP2, LOC101791991, CRISPLD2, SLCO4C1, COL8A1, ZP1, PLA1A
Membrane	1.508972	31	2.24E-02	CC	KCNE4, FASLG, TFCP2L1, IL2RG, OGFRL1, RIMS1, MAP1LC3C, INPP5D, TNFSF10, KRIT1, RAC2, AP4S1, JAK2, HMGCLL1, ANXA2, DST, EDEM1, MAMDC2, VEGFD, LHFPL2, LOC101798586, OSMR, RAB32, CD40LG, LOC101793135, LOC101793812, LOC101793878, HPSE, FBXL2, CTXN1, PTPN3
Cell surface	2.256821	10	3.10E-02	CC	GREM1, SCARB1, SFRP1, CD40LG, ANXA2, LOC101799623, IL2RB, PLAT, IQGAP2, SULF2
Lipopolysaccharide binding	15.49189	6	6.20E-06	MF	SCARB1, SPON2, PTAFR, LY96, LOC101798586, TLR4
Transmembrane signaling receptor activity	4.912063	13	6.38E-06	MF	LOC110351185, CHRNB4, NFAM1, TLR1-A, CD180, LOC101804009, TLR7, CHRFAM7A, CD247, TLR4, TLR3, LOC101792606, TLR2
Transmembrane receptor protein tyrosine kinase activity	3.963042	11	3.14E-04	MF	LYN, BLK, CSF1R, ITK, LOC119714503, LOC101795007, FLT3, TXK, LOC101798888, JAK2, MET

Tumor necrosis factor receptor binding	6.777703	7	3.19E-04	MF	CD40LG, TRAF5, TNFSF10, TNFSF8, FASLG, TRAF1, TNFSF13B
Non-membrane spanning protein tyrosine kinase activity	4.957405	8	7.68E-04	MF	LYN, BLK, ITK, LOC119714503, LOC101795007, TXK, LOC101798888, JAK2
Lipopolysaccharide receptor activity	15.49189	4	1.01E-03	MF	SCARB1, PTAFR, LY96, TLR4
Chemokine activity	4.714924	7	2.73E-03	MF	CCL22, LOC101791385, LOC101802825, LOC101791569, CCL19, LOC101802909, CCL26
C-C chemokine receptor activity	10.32793	4	4.59E-03	MF	LOC101800429, LOC101800757, CCR7, CXCR6
Cytokine receptor activity	5.163964	6	4.63E-03	MF	IL2RB, LOC101793812, IL2RG, IL7R, OSMR, IL13RA1
Heparin binding	4.016416	7	6.43E-03	MF	CLEC3B, SFRP1, ECM2, GPNMB, SERPINC1, CRISPLD2, FN1
Carbonate dehydratase activity	5.95842	5	7.63E-03	MF	LOC113840251, CA5A, LOC101801612, CA4, CA8
Signaling receptor activity	3.498169	7	1.29E-02	MF	OGFRL1, LOC101797774, TNFRSF9, ITGB2, LOC101789696, ITGB6, ANTXR1
Calcium-dependent protein binding	5.163964	5	1.32E-02	MF	ANXA1, ANXA2, ANXA7, CALM2, S100A11
Integrin binding	4.041363	6	1.39E-02	MF	LOC119714503, GPNMB, LOC101799623, ANXA7, IGF1, ITGB6

Calcium ion binding	1.510352	35	1.48E-02	MF	NECAB1, MCFD2, CETN3, FBLN1, IQGAP1, AOAH, LOC101794571, CRACR2A, CLEC3B, ANXA7, LOC101802995, S100A11, UNC13C, CASR, ANXA1, ANXA2, DST, EDEM1, EPDR1, TBC1D9, PLA2G4A, LOC101797893, LOC101796441, DLK1, ASPN, SULF2, SMOC2, EHD4, DCHS2, GPD2, LPCAT2, LCP1, FKBP9, CALM2, CAPS2
Cytokine activity	3.098378	7	2.28E-02	MF	GREM1, KITLG, EDN1, CD40LG, IL15, IL18, TNFSF8
Carbohydrate binding	2.278219	10	2.95E-02	MF	GALNT2, LOC101801190, LOC101794236, LOC101803217, LOC101792733, LY75, GALM, LOC101801378, GANC, GALNT10
Chemokine receptor activity	5.633415	4	2.97E-02	MF	CX3CR1, XCR1, ACKR4, LOC101800940
