# Supplementary material for: Dynamic transcriptomic landscape of myogenesis in Muscovy ducks (Cairina moschata): integrative analysis of hub genes post-hatching
Source: Anim Biosci. 2025 Aug 12;39(1):250159. doi: 10.5713/ab.25.0159 (PMC12754469; doi:10.5713/ab.25.0159)
Supplement: Supplementary file 3 [file ab-25-0159-Supplementary-3.pdf]

**Supplemental file 3.** GO enrichment analysis for down-regulated genes in 80D					
Term	Enrichment	PValue	Count	Class	Genes
Regulation of ion transmembrane transport	2.552399	0.015313	10	BP	KCNG2, KCNH6, KCND2, KCNC1, LOC101796710, LOC101802711, LOC101803973, SCN5A, SCN3B, CACNA1E
Regulation of blood vessel diameter	7.759292	0.012326	4	BP	UTS2R, P2RY2, LOC101797717, AGTR2
Skeletal system development	7.759292	0.012326	4	BP	HDAC4, COL1A2, FST, WFIKKN2
Collagen metabolic process	14.54867	0.01475	3	BP	SMPD3, COL1A2, P3H2
Negative regulation of osteoblast proliferation	9.699115	0.034431	3	BP	NELL1, TNN, BCL2
Positive regulation of osteoblast differentiation	3.879646	0.036879	5	BP	FBN2, NELL1, BMP2, CEBPD, SOX11
Ventricular septum morphogenesis	8.313527	0.04659	3	BP	CITED2, SOX11, PROX1
Neuronal stem cell population maintenance	8.313527	0.04659	3	BP	IGF2BP1, PROX1, FOXO3
Collagen trimer	6.119152	2.02E-06	12	CC	COL1A2, COL13A1, COL24A1, COL22A1, COL11A1, COL4A4, LOC101797613, COL12A1, COL9A1, COL4A6, COL10A1, COL4A5

Integral component of plasma membrane	2.312066	6.95E-04	21	CC	CHRM2, NLGN1, ABCC2, TNFRSF13B, TFRC, NTRK3, GPR20, SLC6A1, SLC9A1, TMEM266, SLC6A6, LOC101796234, ADGRB3, IYD, SSPN, SLC16A8, EPHB2, LOC101802145, TRABD2B, LOC119713904, SLC19A1
Extracellular region	1.853482	9.08E-05	44	CC	FBN2, WNT2B, LEAP2, COL12A1, FSTL4, MLN, SMPD3, ACAN, LOC101794476, FSTL5, C8G, OLFML3, APOH, STC2, COL10A1, CCN3, APOD, WNT2, SNCA, GDF10, IGFBP1, LOC101789646, BDNF, IGFBP4, FST, IGFBP3, NRG1, LOC101798382, SORL1, GREM2, BD2, BMP2, TG, OLFM3, ANGPTL7, LOC101798108, MFAP2, PNOC, FIBIN, PI15, CRH, LOC101794528, LOC101802664, FMOD
Membrane	1.709763	0.008042	27	CC	PCSK2, SEMA7A, AMER3, KCNE3, RRAD, LOC101802338, ADD3, MTMR4, SRL, MTMR7, LOC101805201, PARD6A, ACVR1C, SNCA, LOC101802900, LAMB1, NPNT, DGKZ, PEX5L, COL4A4, LOC101797613, COL4A6, COL4A5, LOC101802664, LOC101800880, FERMT2, SNTB1
Glutamatergic synapse	2.583642	0.033497	8	CC	NRP2, NLGN1, CALB1, ACTC1, CTBP2, SYN3, LRRC4C, PCDH17
Integral component of postsynaptic membrane	9.688657	0.034557	3	CC	SLC6A6, NRP2, PCDH17
Plasma membrane	1.312826	0.036933	50	CC	TENM1, OXTR, TFRC, KCNK9, GPR61, GLDN, BRS3, CDH7, SMPD3, PANX2, GRM7, LOC101793345, IYD, DNER, GRM8, CHP1, UTS2R, RNF43, AMN, SOX11, GFRA1, TACR1, SSTR2, RGMA, ENAH, MTNR1A, CLDN14, ADGRB3, CDHR1, AGTR2, PLPP3, CDH17, SLC41A3, THY1, PCDH17, SLC9A1, CNR1, P2RY2, KCNN3, KIAA0319, TAS1R3, CORIN, MCOLN3, OPN3, FZD7, SORL1, LOC101805135, PLCH2, TJP3, ADGRL3

Extracellular matrix structural constituent	9.794942	1.93E-07	10	MF	FBN2, FBN3, COL1A2, COL24A1, COL11A1, COL4A4, LOC101797613, COL4A6, COL4A5, LAMB1
Lipid phosphatase activity	8.271284	0.010092	4	MF	PLPPR1, PLPP4, PLPP3, PLPP2
Calcium ion binding	1.607025	0.010146	31	MF	NECAB2, FBN2, FBN3, GUCA1B, LOC101798272, CACNA1E, FSTL4, PCDH17, CDH7, ACAN, FSTL5, CALB1, DNER, CHP1, OIT3, RHBDL3, SLC25A25, KCNIP2, NPNT, NELL1, CDHR1, LOC101794588, CASQ2, LOC119713856, PLCH2, LOC101803972, LOC101794806, MATN4, MYL9, CDH17, MATN2
Voltage-gated potassium channel activity	2.863137	0.020118	8	MF	KCNG2, KCNE1, KCNH6, KCND2, KCNE3, KCNC1, KCNK9, LOC101796710
Growth factor activity	2.355746	0.024885	10	MF	GDF10, BMP2, LOC101789646, BDNF, LOC101798108, OSGIN1, IGF2, NRG1, FGF1, FGF12
Integrin binding	4.045737	0.032096	5	MF	TNN, IGF2, THY1, FGF1, PLPP3
SH3 domain binding	4.962771	0.043052	4	MF	ADAM19, ENAH, UVRAG, ELMO3
