# Supplementary material for: Dynamic transcriptomic landscape of myogenesis in Muscovy ducks (Cairina moschata): integrative analysis of hub genes post-hatching
Source: Anim Biosci. 2025 Aug 12;39(1):250159. doi: 10.5713/ab.25.0159 (PMC12754469; doi:10.5713/ab.25.0159)
Supplement: Supplementary file 4 [file ab-25-0159-Supplementary-4.pdf]

**Supplemental file 4.** KEGG enrichment analysis for up-regulated genes in 80D				
Term	Generatio	P Value	Count	Input
ECM-receptor interaction	0.190476	3.93E-08	16	LAMA1, FRAS1, SDC4, THBS4, COL9A2, COL9A1, NPNT, COL4A6, COL4A5, COL4A4, SPP1, LAMB1, COL1A2, LAMB3, TNN, COL2A1
Valine, leucine and isoleucine degradation	0.244444	6.27E-07	11	IVD, PCCA, HADHB, HADHA, ACAA2, ALDH6A1, BCKDHB, ABAT, HIBADH, MCCC2, MCCC1
Focal adhesion	0.106383	4.13E-06	20	LAMA1, TNN, RASGRF1, MAPK10, THBS4, VAV2, COL9A2, COL9A1, COL4A6, COL4A5, COL4A4, SPP1, PPP1R12B, LAMB1, COL1A2, LAMB3, BCL2, COL2A1, CAV3, MYL9
Metabolic pathways	0.05456	6.64E-06	67	B4GALNT4, ST3GAL1, EPHX2, HADHA, P4HA3, ME3, UPB1, PTDSS1, GALNTL6, PCCA, PTGS1, PYCR3, GLUL, GAMT, PLCE1, DMGDH, ABAT, HIBADH, PMM1, ACADL, DGKZ, NDUFV1, GATM, SMOX, ACAA2, HADHB, SPR, DGAT2, NT5E, PLCH2, PNPLA2, GAL3ST1, SARDH, SHMT1, MTMR7, ACOT12, MTMR4, NT5C2, HACD1, PLPP3, PLPP2, ISYNA1, AMPD1, LARGE2, MAT1A, CHST9, NMNAT2, ALDH6A1, NMRK2, HYKK, ST6GALNAC6, ST6GALNAC4, GK, MCCC2, MCCC1, UROC1, IVD, GOT1, ADCY3, PIPOX, SMPD3, GPX2, BCKDHB, PAH, CPS1, FUT9, PLCD3
Neuroactive ligand-receptor interaction	0.077882	4.23E-05	25	GRIA2, GRIA3, CHRNA10, GABRA4, CRH, UTS2R, CNR1, PTGER3, GRIN3A, GRM8, BRS3, GRM7, CHRNG, GRID2, MLN, CHRM2, LEPR, CHRNA5, P2RY2, MTNR1A, NMUR1, GRIN2B, SSTR4, OXTR, AGTR2
Wnt signaling pathway	0.104167	8.01E-05	15	RSPO3, WNT2B, SOST, AXIN2, SFRP4, FZD7, WNT2, RNF43, DVL3, MAPK10, CTNND2, CXXC4, APC2, CTBP2, MYC
Adrenergic signaling in cardiomyocytes	0.101695	4.93E-04	12	KCNE1, ADCY3, ACTC1, RYR2, ATP1B1, RAPGEF4, ATP1A1, AGTR2, PLN, SCN4B, CACNG5, BCL2
TGF-beta signaling pathway	0.114943	6.02E-04	10	GREM2, BMP2, ACVR1C, SMAD7, ID2, FST, FMOD, PITX2, MYC, RGMA

Arginine and proline metabolism	0.171429	1.22E-03	6	GAMT, SMOX, GATM, P4HA3, PYCR3, GOT1
Glycine, serine and threonine metabolism	0.162162	1.58E-03	6	GAMT, PIPOX, GATM, SARDH, DMGDH, SHMT1
Fatty acid elongation	0.185185	2.33E-03	5	HACD1, HADHB, HADHA, ACAA2, ACOT7
Cell adhesion molecules (CAMs)	0.092593	2.69E-03	10	LRRC4C, CLDN19, CLDN14, PTPRF, NLGN1, SDC4, IGSF11, NECTIN3, NFASC, CADM1
Glycerolipid metabolism	0.12069	3.04E-03	7	DGKZ, PLPP3, PLPP2, PLPP4, DGAT2, PNPLA2, GK
Propanoate metabolism	0.166667	3.49E-03	5	PCCA, ALDH6A1, HADHA, BCKDHB, ABAT
Biosynthesis of amino acids	0.114754	3.92E-03	7	MAT1A, PAH, GLUL, PYCR3, GOT1, CPS1, SHMT1
beta-Alanine metabolism	0.16129	3.95E-03	5	UPB1, ALDH6A1, HADHA, SMOX, ABAT
Inositol phosphate metabolism	0.101449	7.23E-03	7	ISYNA1, PLCH2, ALDH6A1, PLCE1, PLCD3, MTMR7, MTMR4
Calcium signaling pathway	0.068571	1.00E-02	12	ADCY3, PTGER3, RYR2, CHRM2, CACNA1G, CACNA1E, PLCE1, CASQ2, OXTR, PLN, SLC25A4, PLCD3
Nicotinate and nicotinamide metabolism	0.153846	1.13E-02	4	NMNAT2, NT5E, NT5C2, NMRK2
AGE-RAGE signaling pathway in diabetic complications	0.084211	1.15E-02	8	COL4A6, COL4A5, COL4A4, PLCE1, PLCD3, MAPK10, COL1A2, BCL2
Glycosphingolipid biosynthesis - ganglio series	0.214286	1.29E-02	3	ST6GALNAC6, ST3GAL1, ST6GALNAC4
Carbon metabolism	0.081633	1.35E-02	8	PCCA, HADHA, ALDH6A1, ME3, ME2, GOT1, CPS1, SHMT1
MAPK signaling pathway	0.059289	1.42E-02	15	IGF2, DDIT3, SRF, RASGRF1, FGF5, NR4A1, CACNA1E, CACNA1G, MAP2K3, MAPK10, MYC, BDNF, DUSP1, CACNG5, FGF1
PPAR signaling pathway	0.096774	1.53E-02	6	ACADL, ANGPTL4, FABP3, ME3, GK, APOA1

Cardiac muscle contraction	0.095238	1.63E-02	6	CASQ2, ACTC1, RYR2, ATP1B1, ATP1A1, CACNG5
Phenylalanine, tyrosine and tryptophan biosynthesis	0.4	1.66E-02	2	PAH, GOT1
Fatty acid degradation	0.133333	1.75E-02	4	HADHB, ACAA2, HADHA, ACADL
Sphingolipid metabolism	0.104167	2.00E-02	5	NEU4, GAL3ST1, PLPP3, PLPP2, SMPD3
Arginine biosynthesis	0.176471	2.02E-02	3	GLUL, GOT1, CPS1
Alanine, aspartate and glutamate metabolism	0.121212	2.32E-02	4	GLUL, GOT1, CPS1, ABAT
RIG-I-like receptor signaling pathway	0.096154	2.65E-02	5	MAPK10, PIN1, NFKBIA, IRF7, DHX58
Fatty acid metabolism	0.09434	2.83E-02	5	HACD1, HADHB, HADHA, ACADL, ACAA2
Adipocytokine signaling pathway	0.086207	3.86E-02	5	NFKBIA, MAPK10, IRS1, SOCS3, LEPR
Notch signaling pathway	0.093023	4.99E-02	4	HES1, CTBP2, NCOR2, DVL3
